# Supplementary figures and images for: The Lon protease temporally restricts polar cell differentiation events during the Caulobacter cell cycle
Source: eLife. 2021 Oct 25;10:e73875. doi: 10.7554/eLife.73875 (PMC8545394; doi:10.7554/eLife.73875)

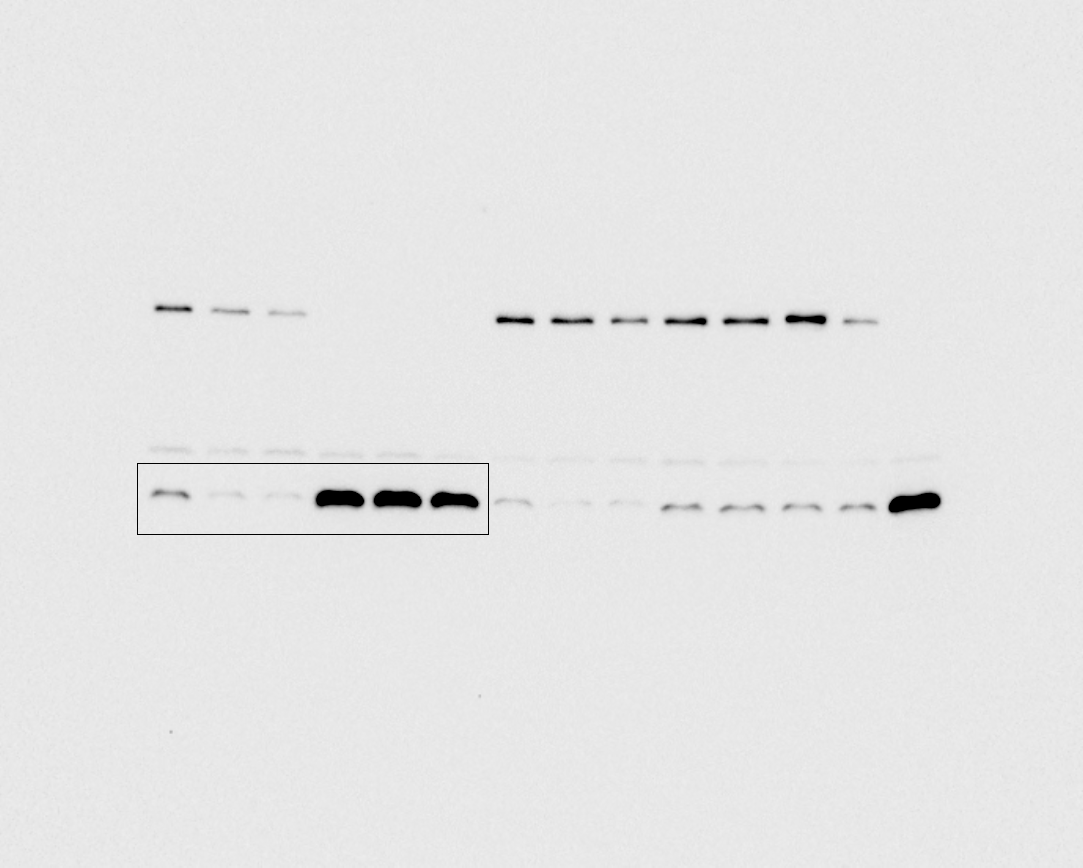

Supplement: Figure 1—source data 2. [file elife-73875-fig1-data2.zip › Figure 1 - source data/Figure 1 panel A/anti-Lon and anti-CcrM/anti-Lon and anti-CcrM - labelled CcrM crop out.tif]

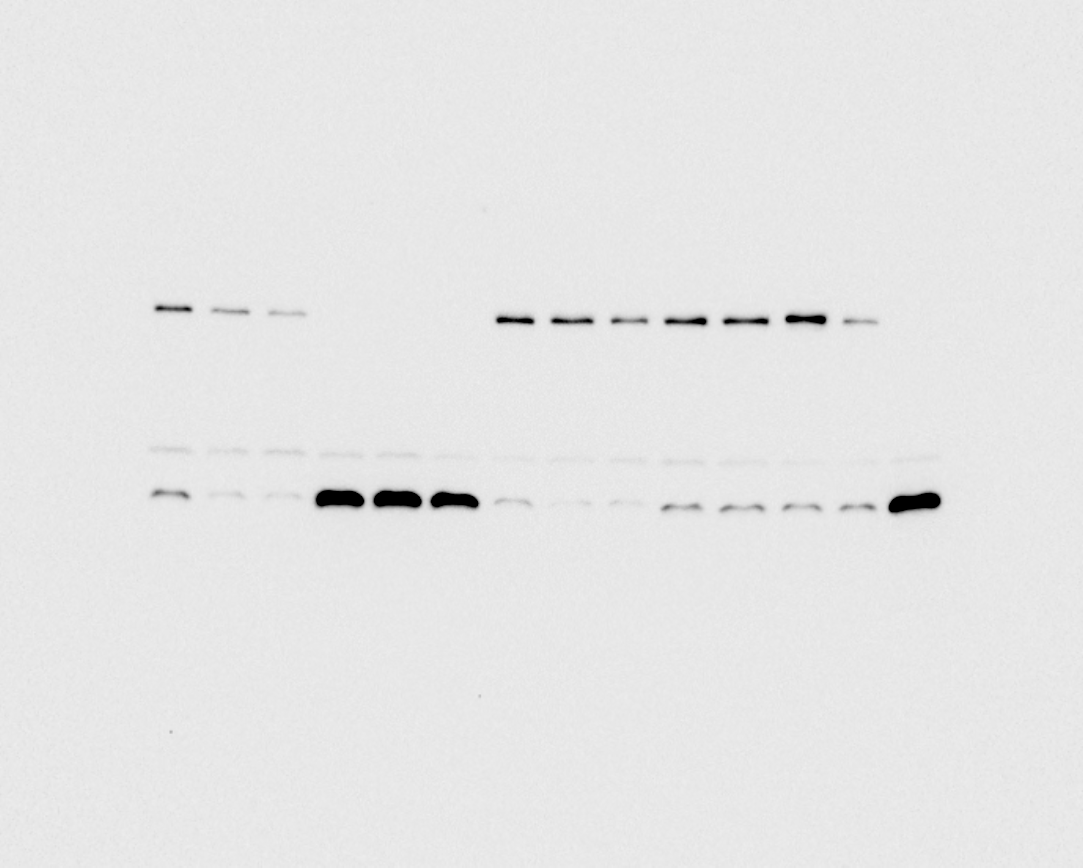

Supplement: Figure 1—source data 2. [file elife-73875-fig1-data2.zip › Figure 1 - source data/Figure 1 panel A/anti-Lon and anti-CcrM/anti-Lon and anti-CcrM.tif]

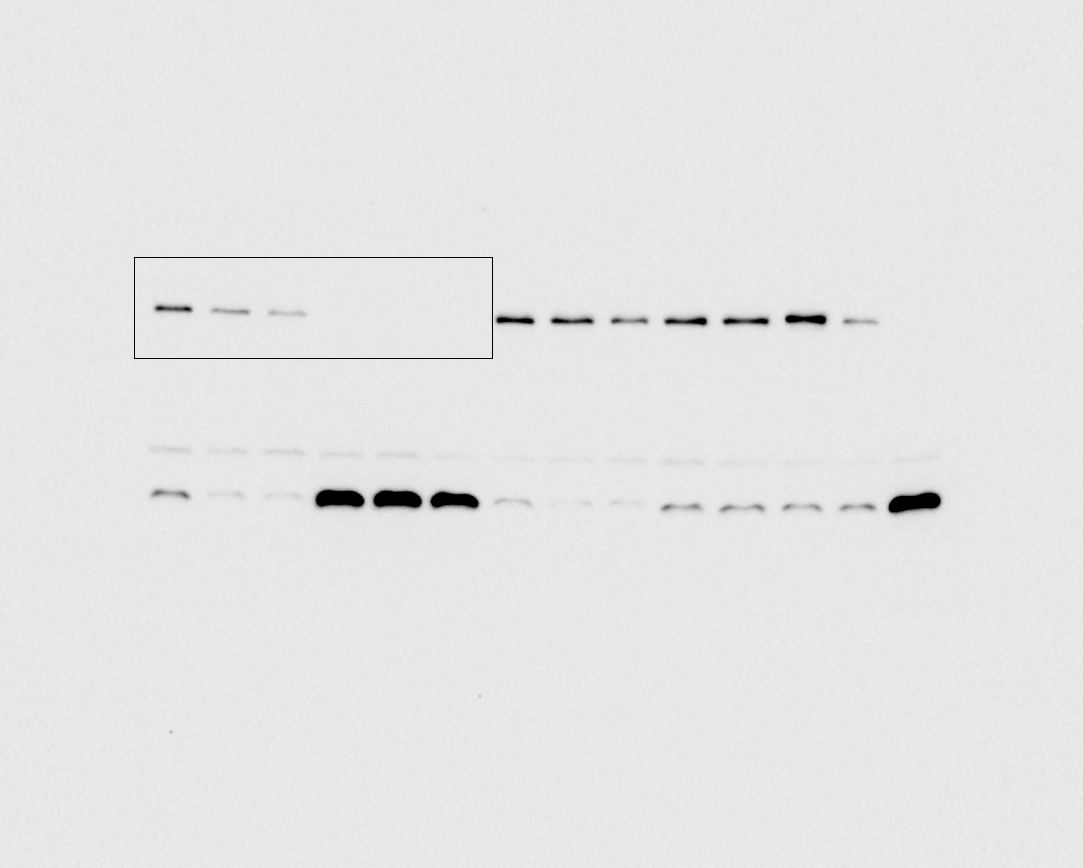

Supplement: Figure 1—source data 2. [file elife-73875-fig1-data2.zip › Figure 1 - source data/Figure 1 panel A/anti-Lon and anti-CcrM/anti-Lon and anti-CcrM - labelled Lon crop out.tif]

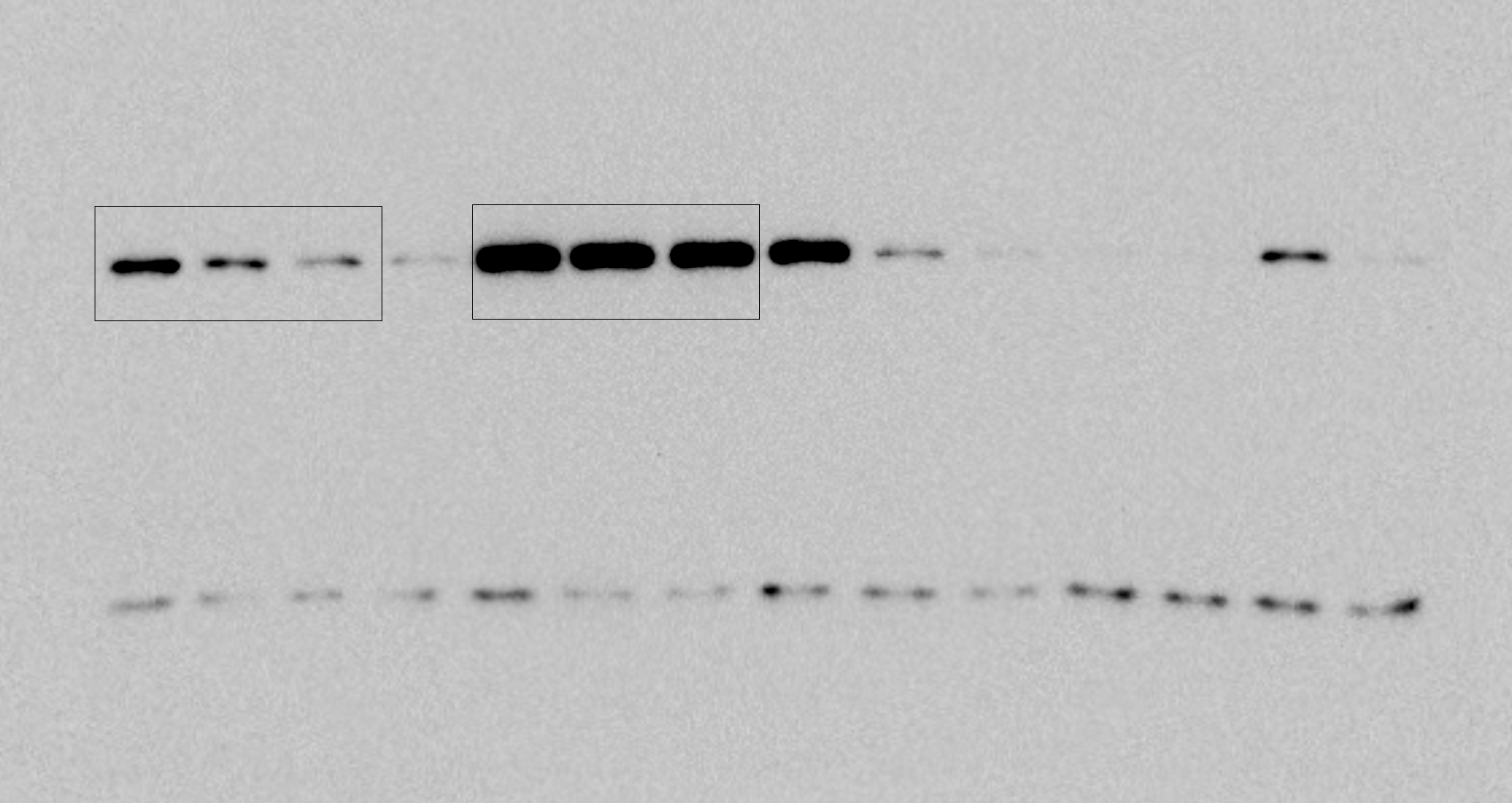

Supplement: Figure 1—source data 2. [file elife-73875-fig1-data2.zip › Figure 1 - source data/Figure 1 panel A/anti-DnaA/anti-DnaA - labelled.tif]

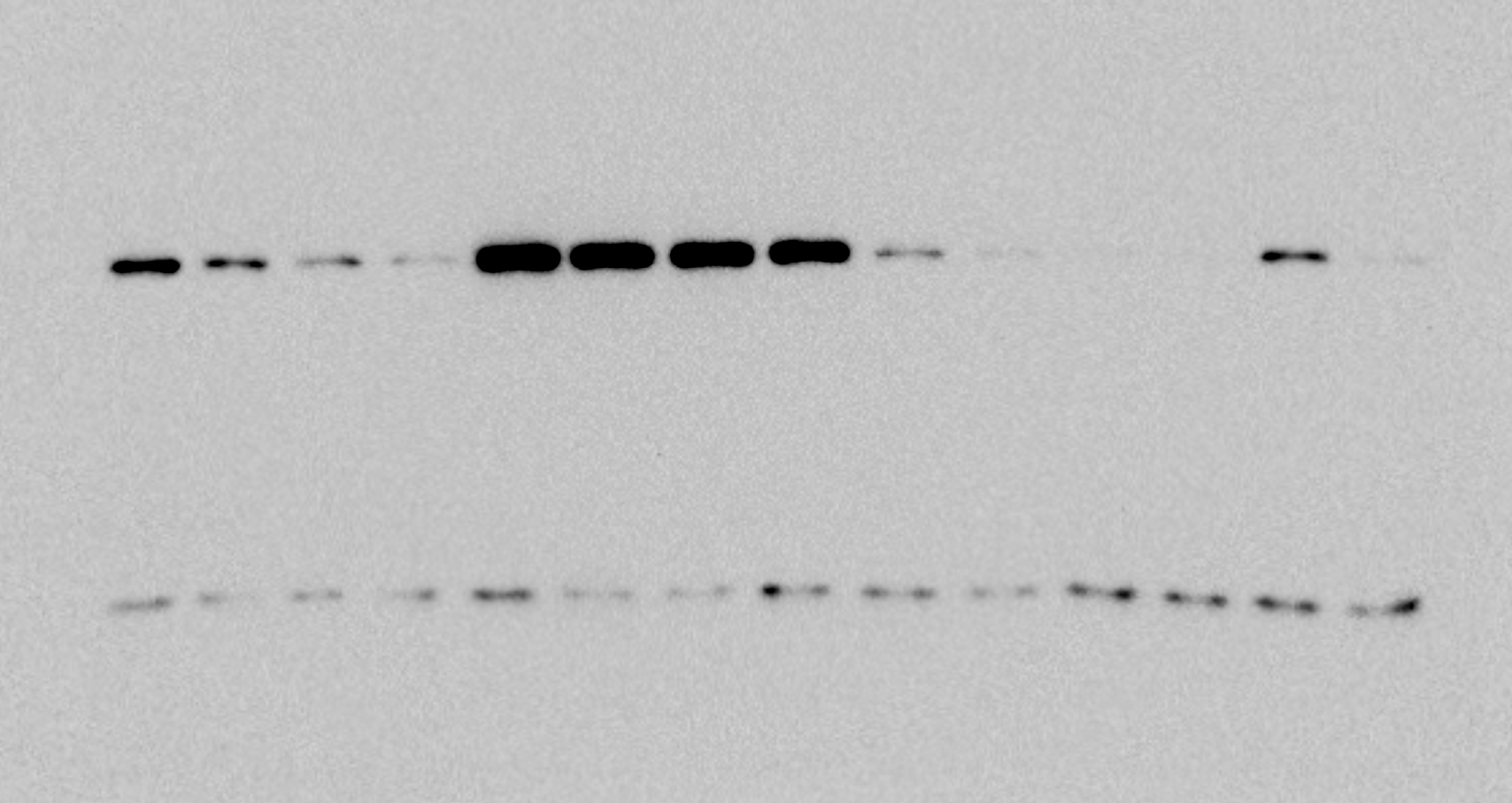

Supplement: Figure 1—source data 2. [file elife-73875-fig1-data2.zip › Figure 1 - source data/Figure 1 panel A/anti-DnaA/anti-DnaA.tif]

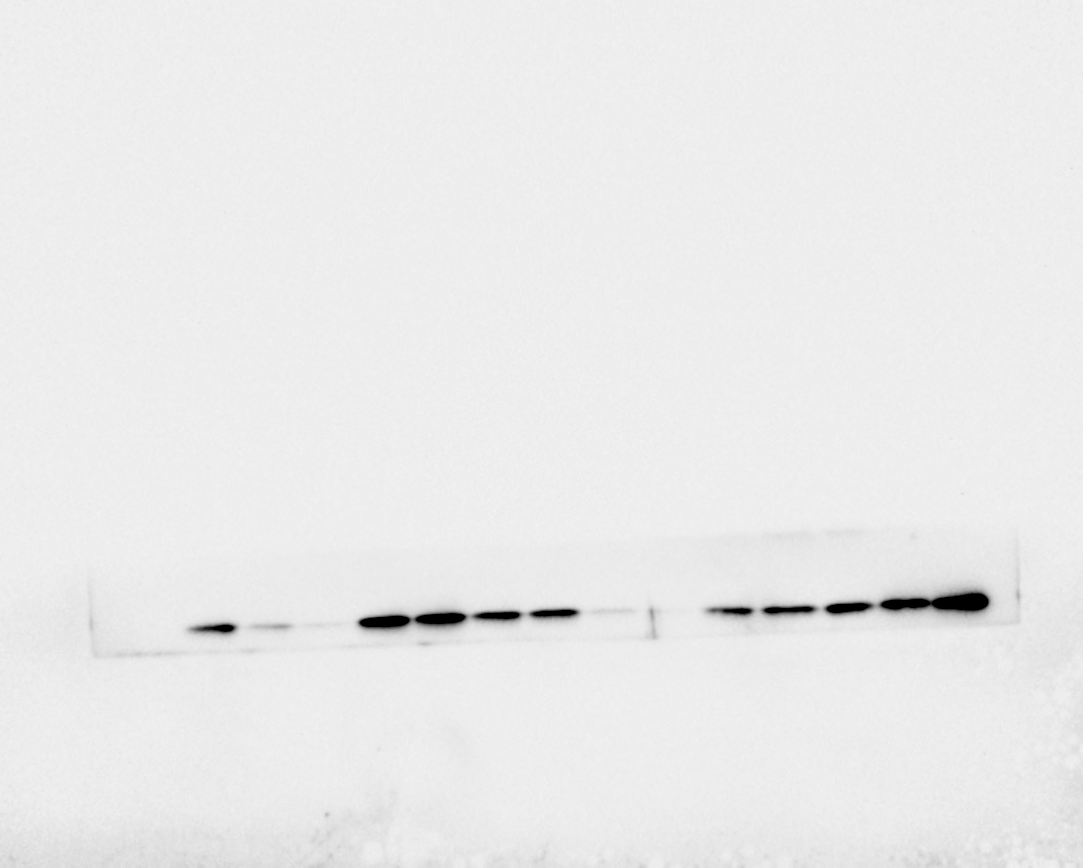

Supplement: Figure 1—source data 2. [file elife-73875-fig1-data2.zip › Figure 1 - source data/Figure 1 panel A/anti-SciP/anti-SciP.tif]

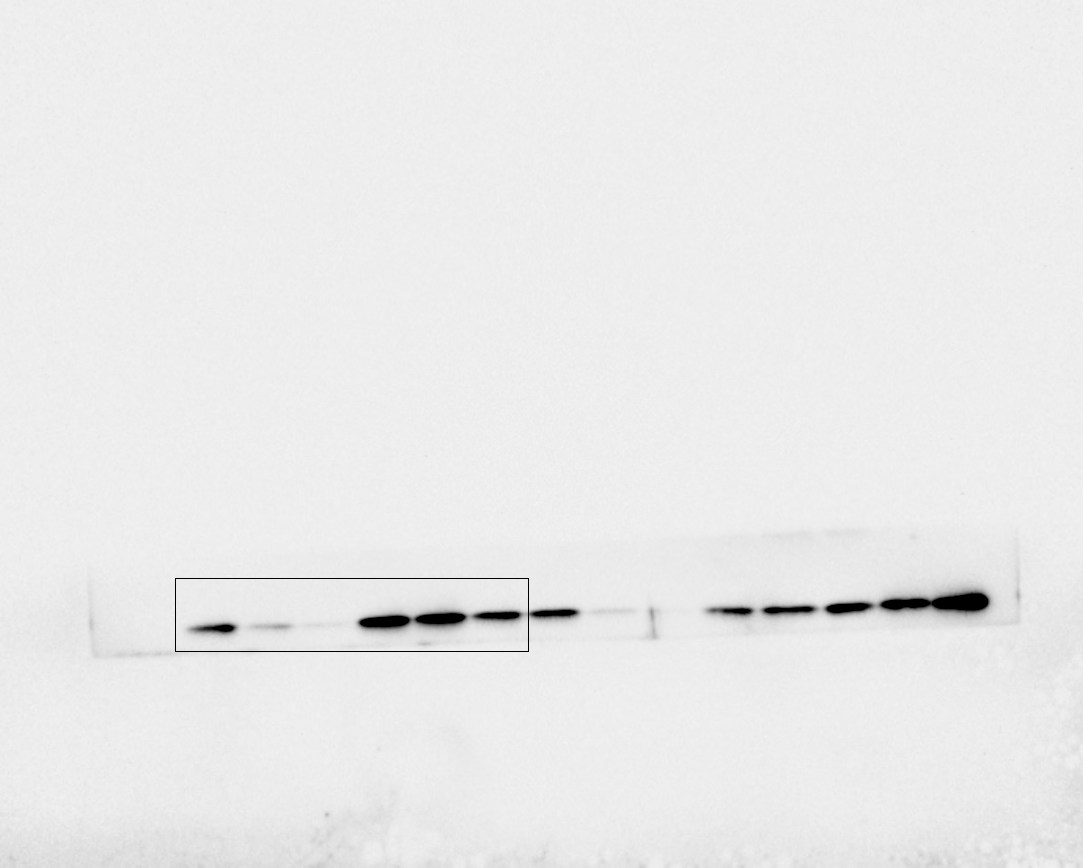

Supplement: Figure 1—source data 2. [file elife-73875-fig1-data2.zip › Figure 1 - source data/Figure 1 panel A/anti-SciP/anti-SciP - labelled.tif]

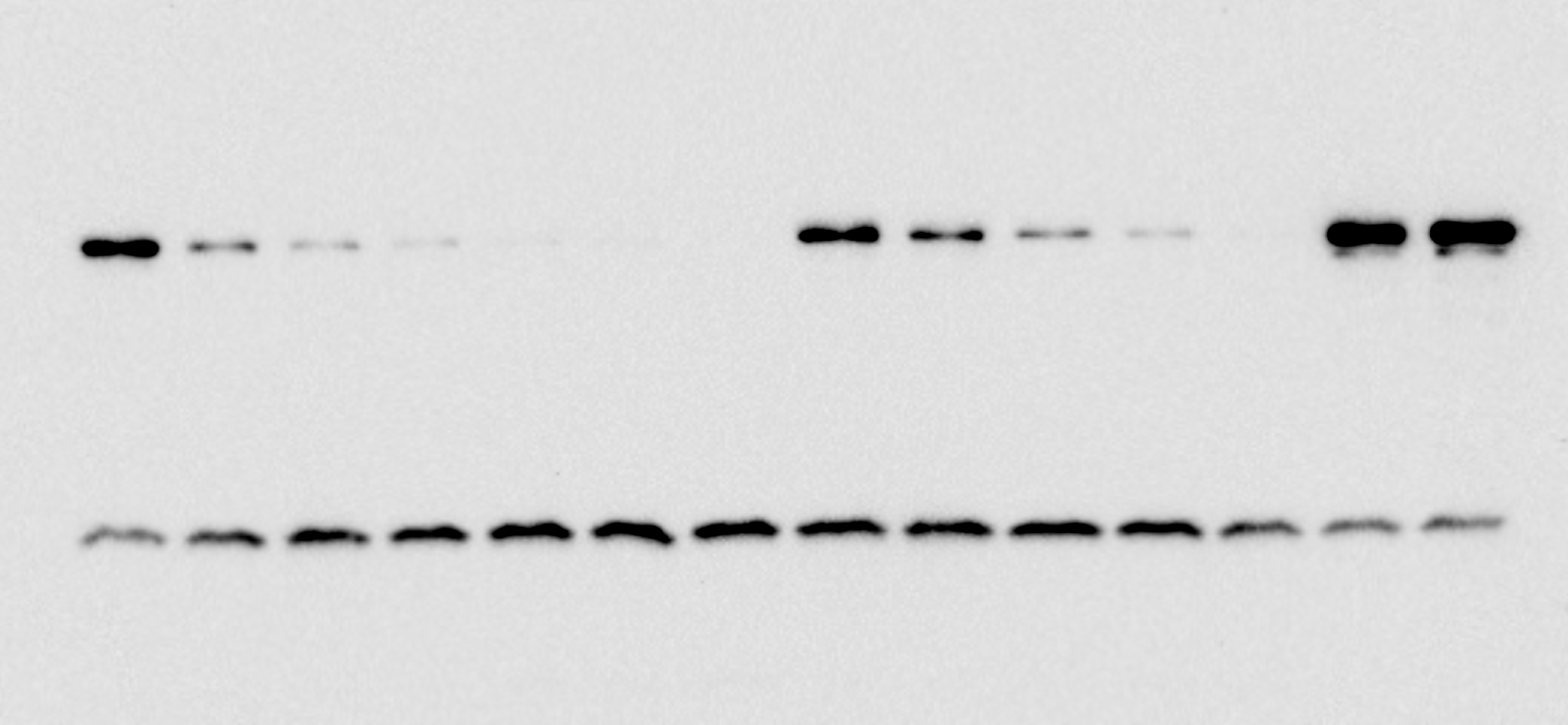

Supplement: Figure 1—source data 2. [file elife-73875-fig1-data2.zip › Figure 1 - source data/Figure 1 panel B/anti-Lon/anti-Lon.tif]

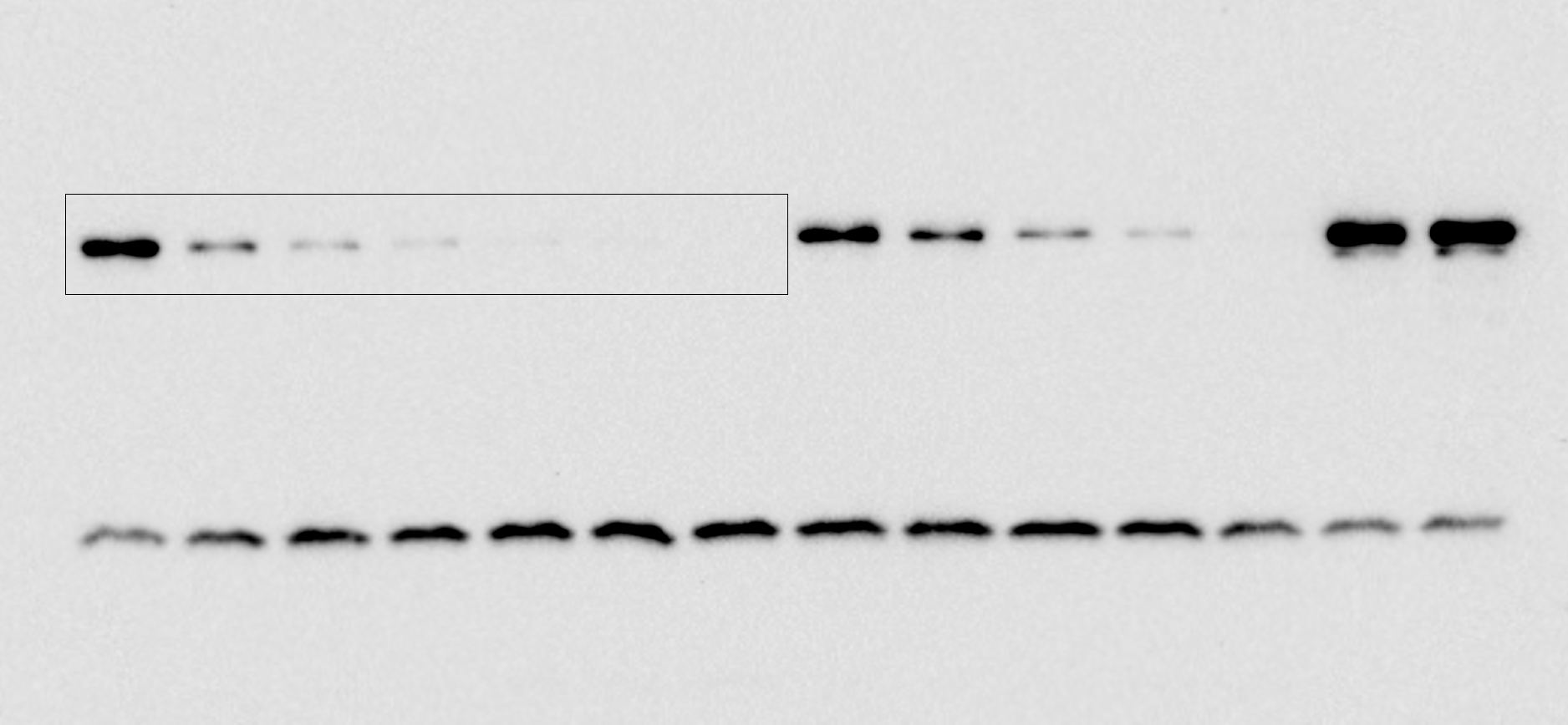

Supplement: Figure 1—source data 2. [file elife-73875-fig1-data2.zip › Figure 1 - source data/Figure 1 panel B/anti-Lon/anti-Lon - labelled.tif]

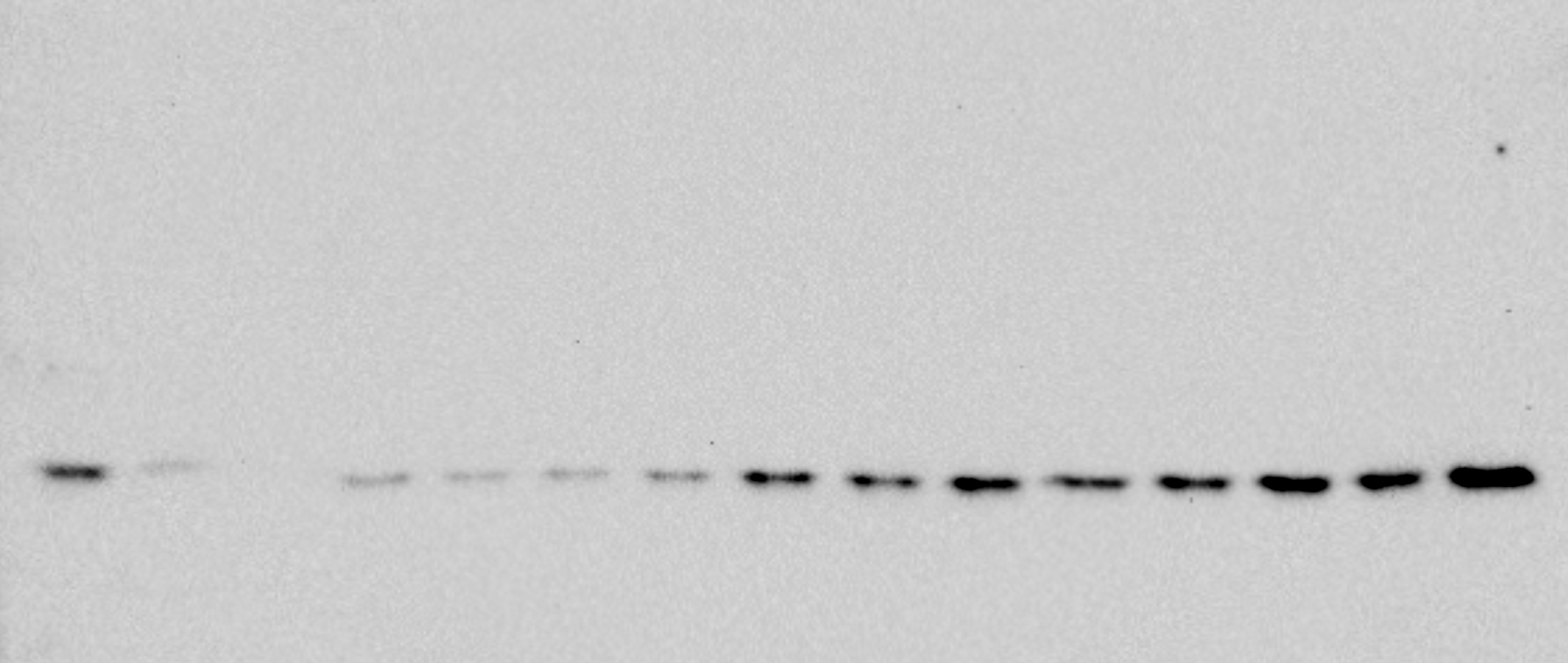

Supplement: Figure 1—source data 2. [file elife-73875-fig1-data2.zip › Figure 1 - source data/Figure 1 panel B/anti-CcrM/anti-CcrM.tif]

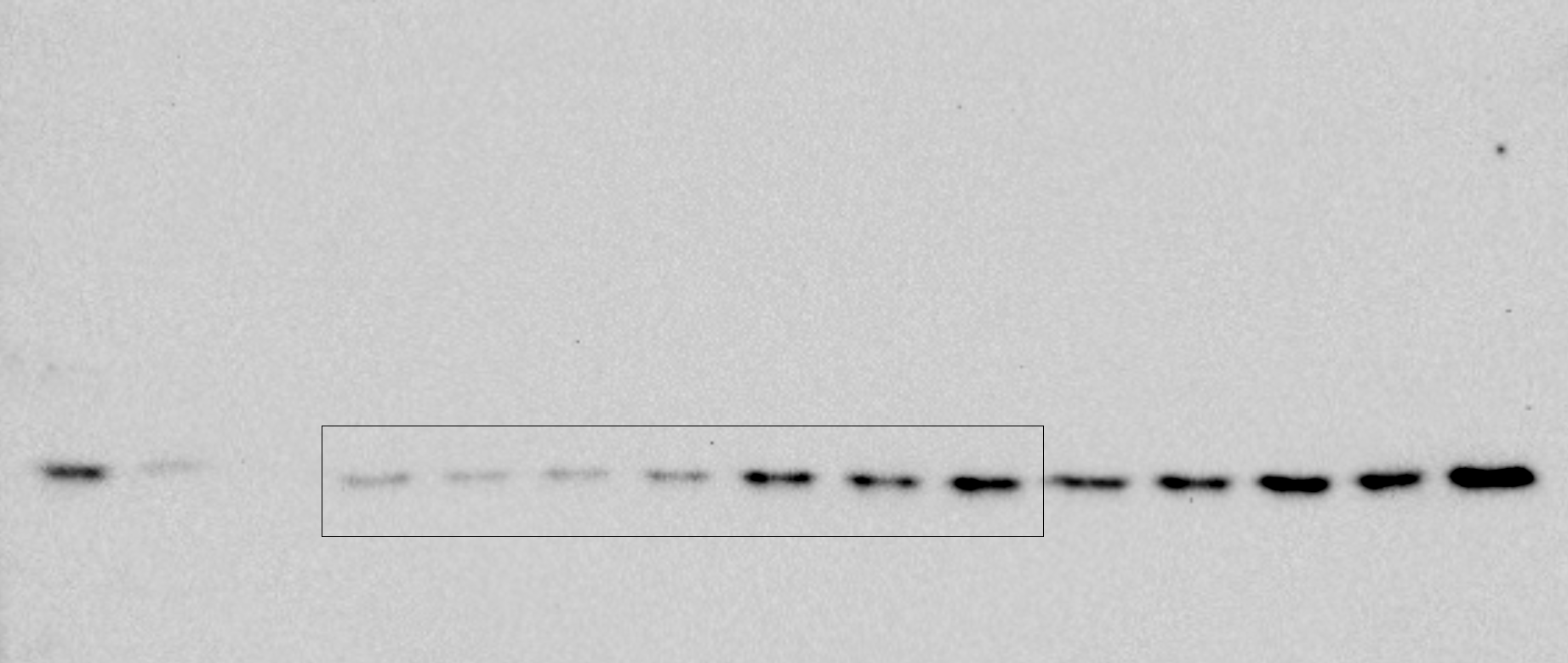

Supplement: Figure 1—source data 2. [file elife-73875-fig1-data2.zip › Figure 1 - source data/Figure 1 panel B/anti-CcrM/anti-CcrM - labelled.tif]

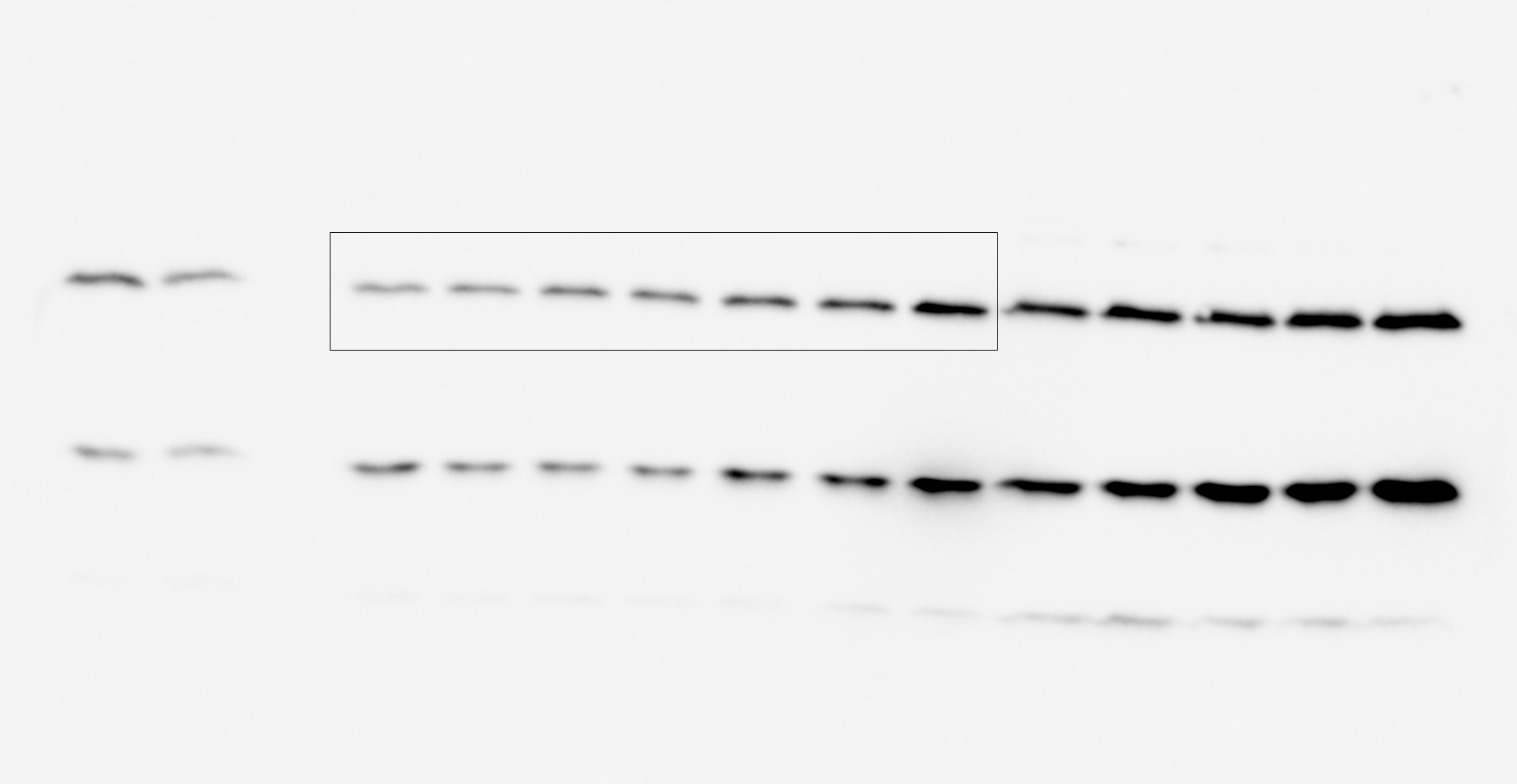

Supplement: Figure 1—source data 2. [file elife-73875-fig1-data2.zip › Figure 1 - source data/Figure 1 panel B/anti-DnaA/anti-DnaA - labelled.tif]

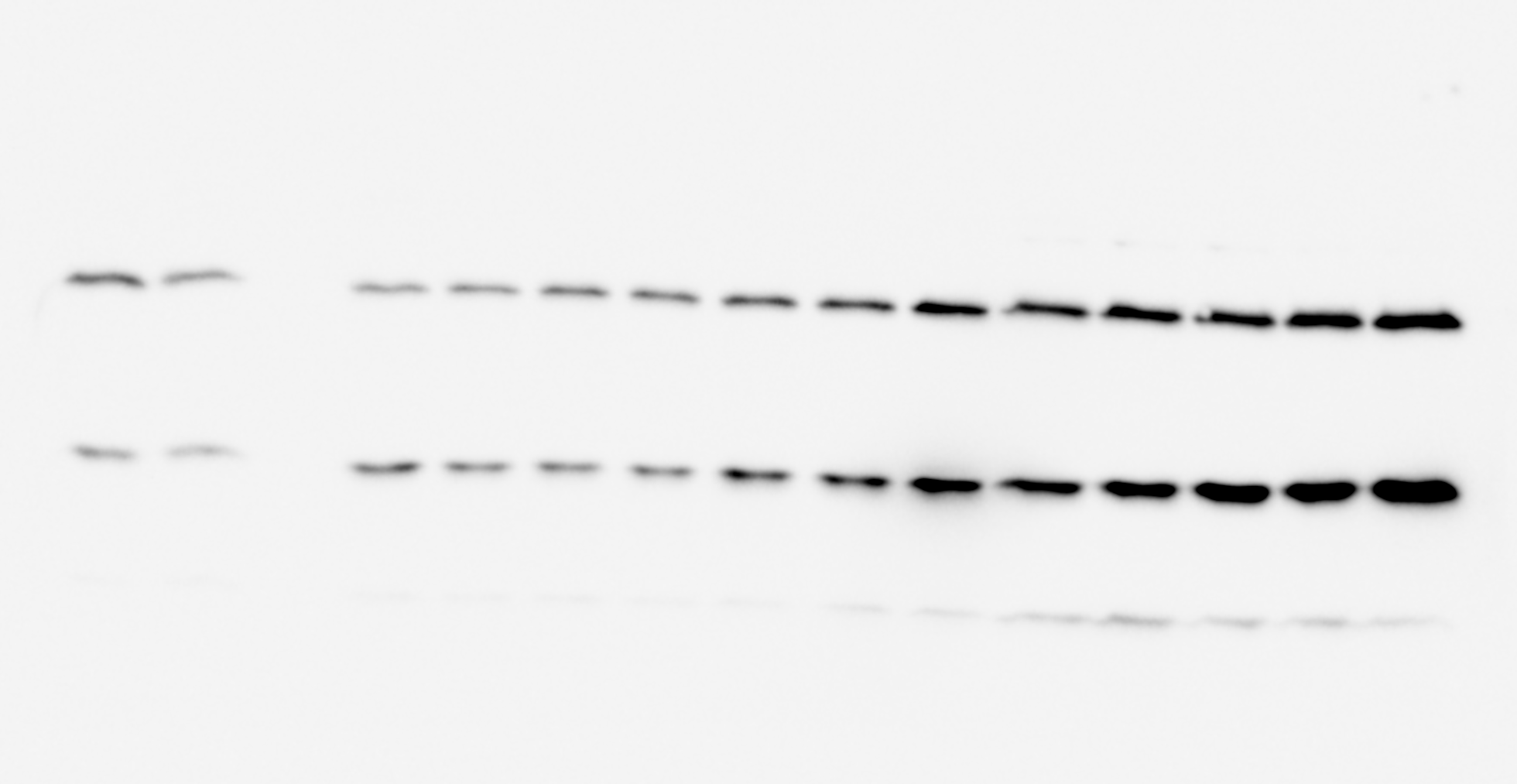

Supplement: Figure 1—source data 2. [file elife-73875-fig1-data2.zip › Figure 1 - source data/Figure 1 panel B/anti-DnaA/anti-DnaA.tif]

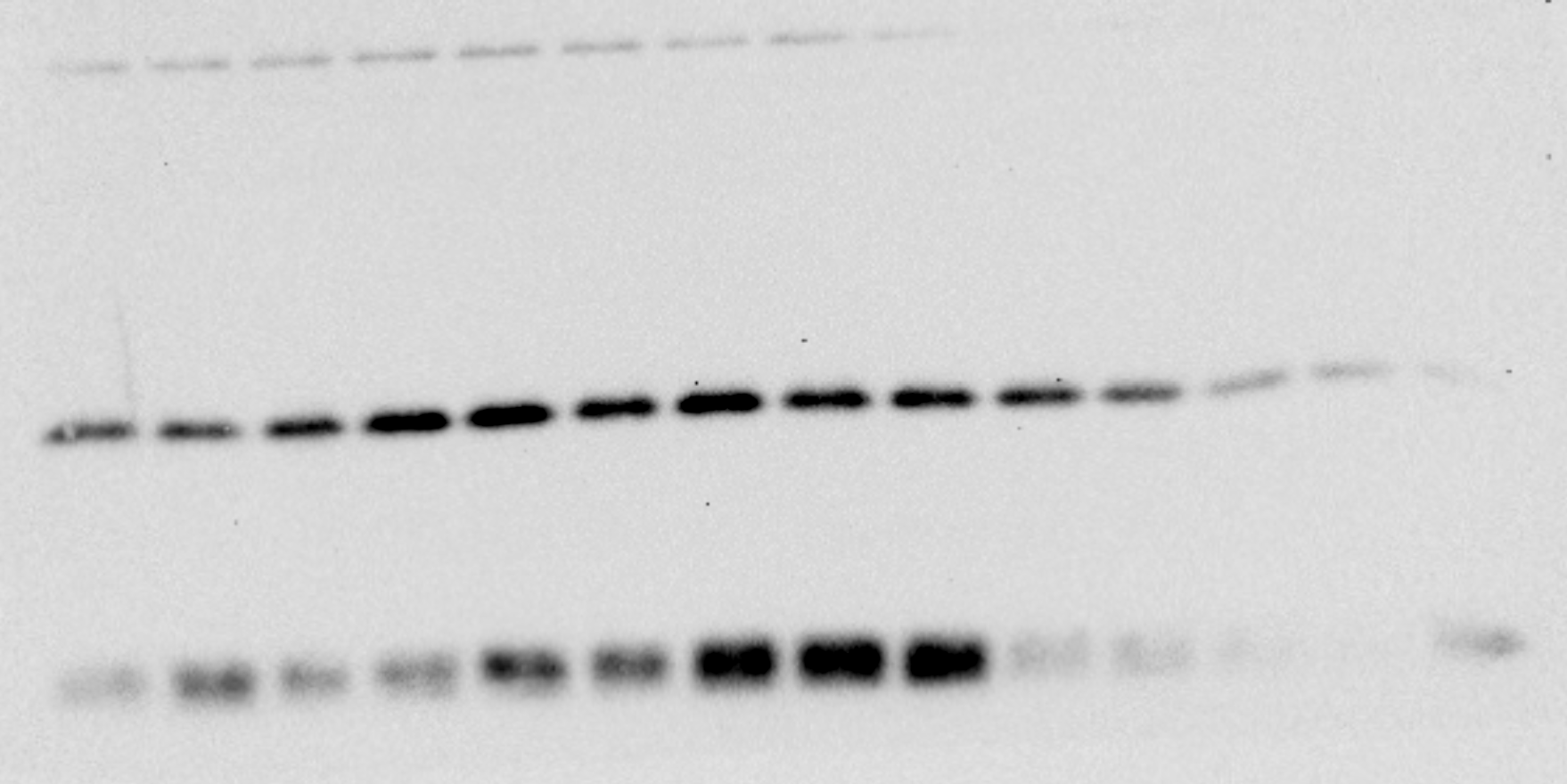

Supplement: Figure 1—source data 2. [file elife-73875-fig1-data2.zip › Figure 1 - source data/Figure 1 panel B/anti-SciP/anti-SciP.tif]

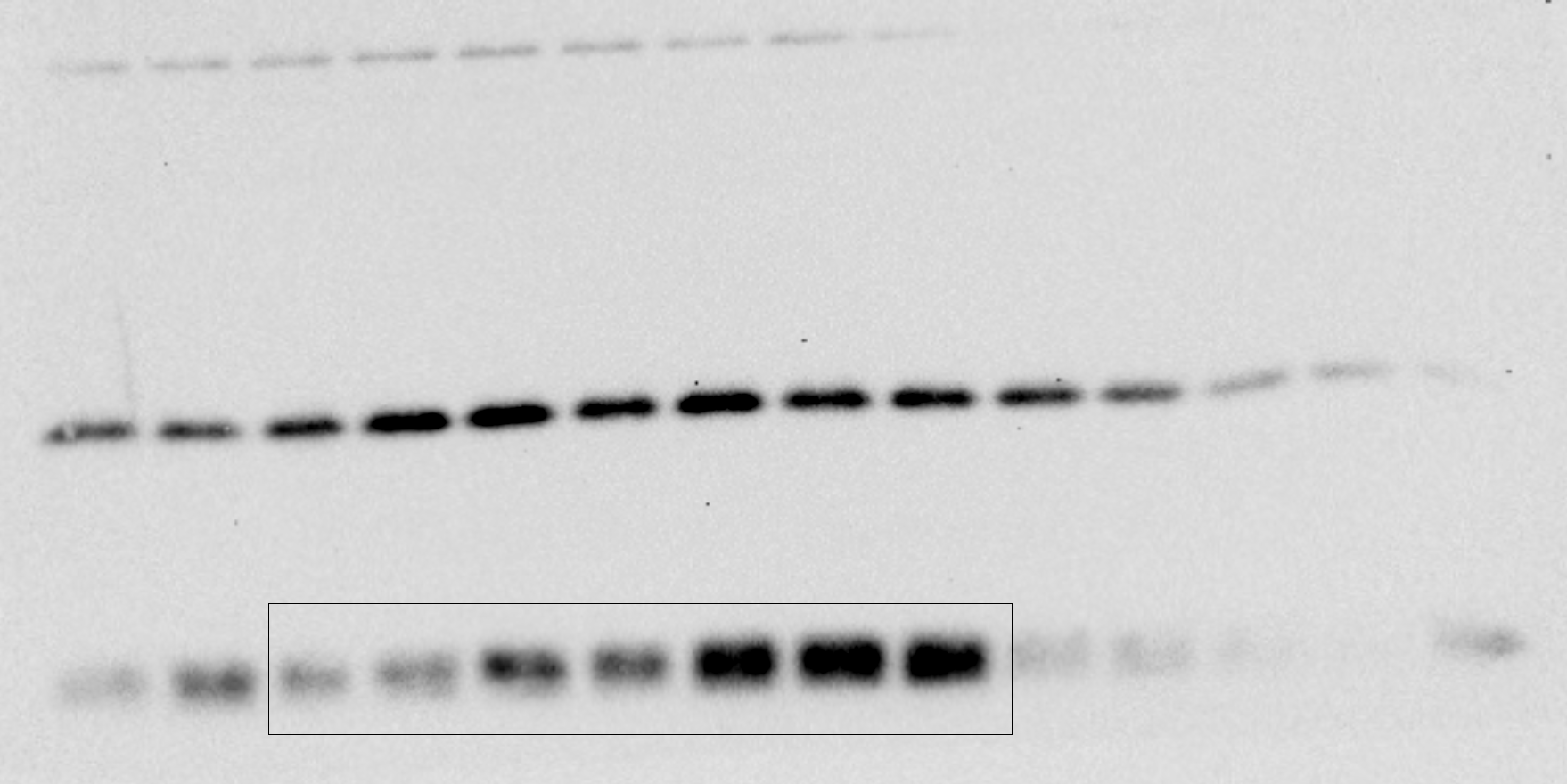

Supplement: Figure 1—source data 2. [file elife-73875-fig1-data2.zip › Figure 1 - source data/Figure 1 panel B/anti-SciP/anti-SciP - labelled.tif]

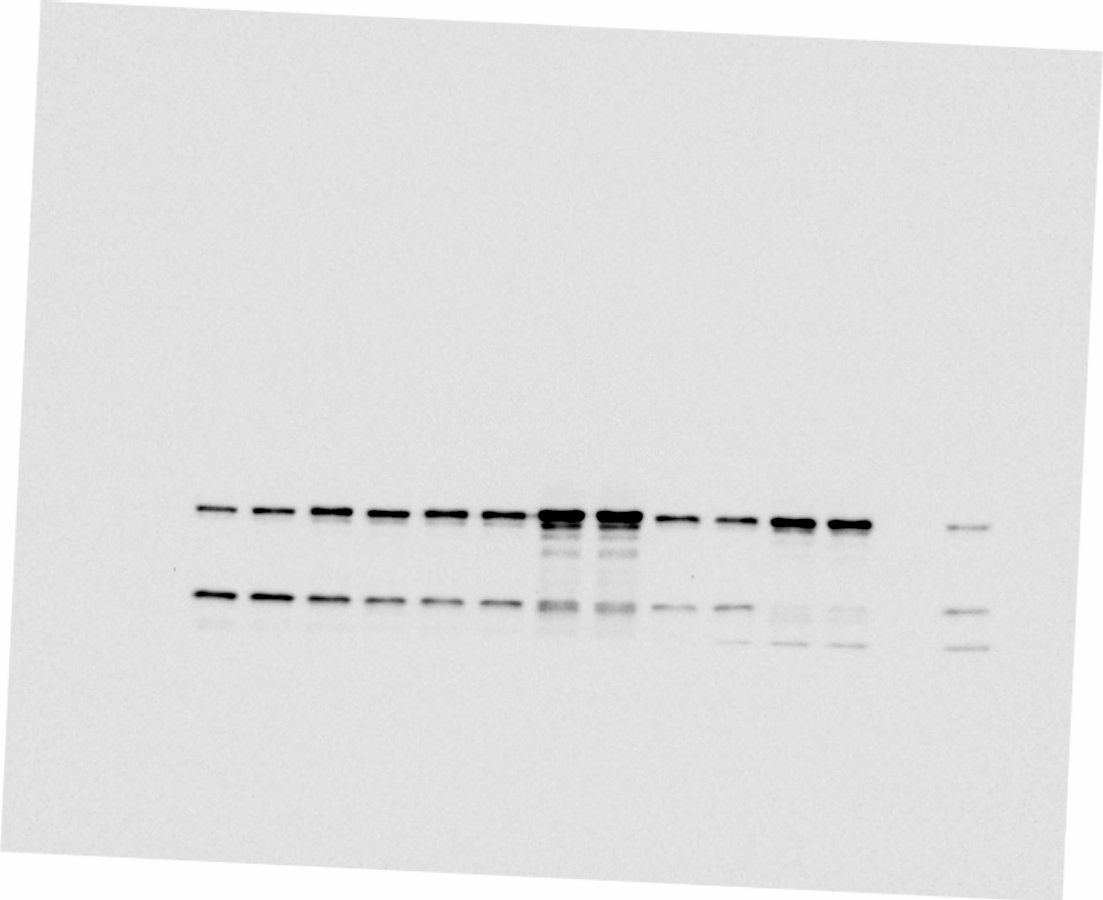

Supplement: Figure 1—source data 2. [file elife-73875-fig1-data2.zip › Figure 1 - source data/Figure 1 panel C/anti-Lon/anti-Lon.tif]

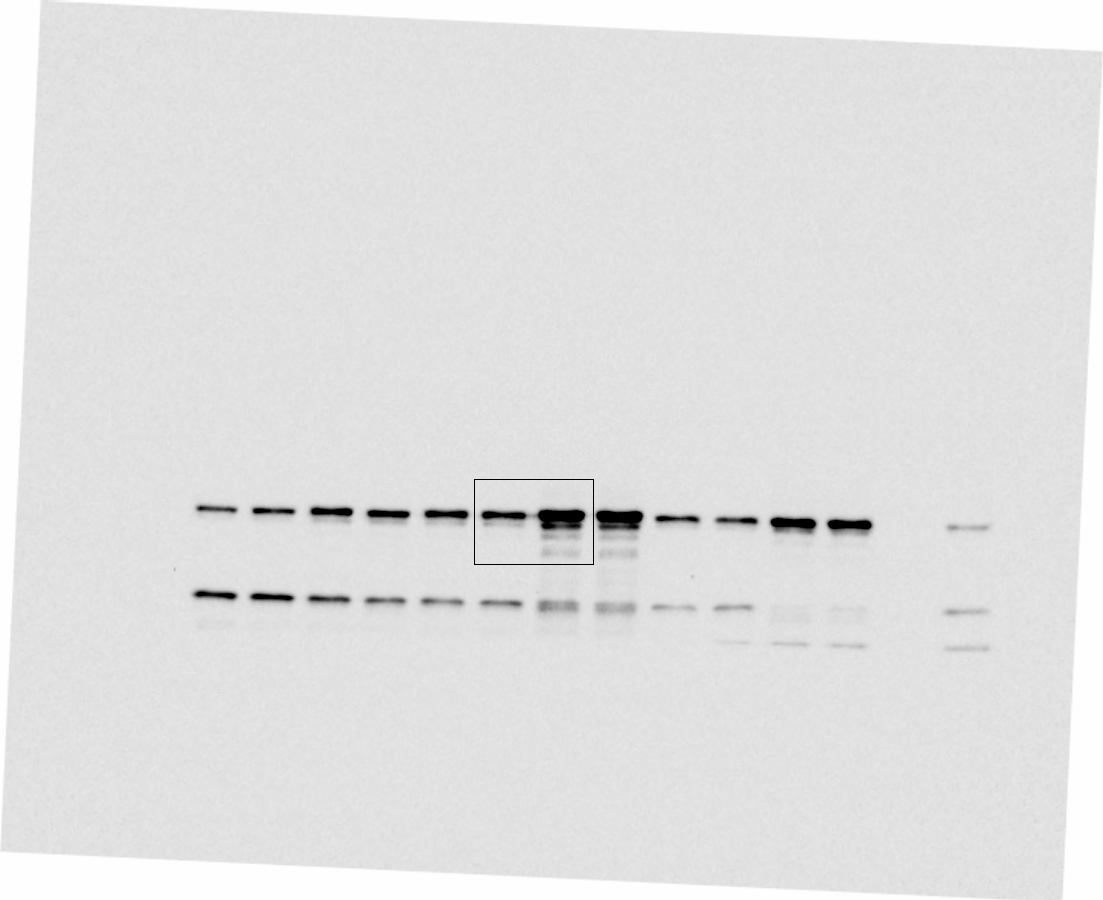

Supplement: Figure 1—source data 2. [file elife-73875-fig1-data2.zip › Figure 1 - source data/Figure 1 panel C/anti-Lon/anti-Lon - labelled.tif]

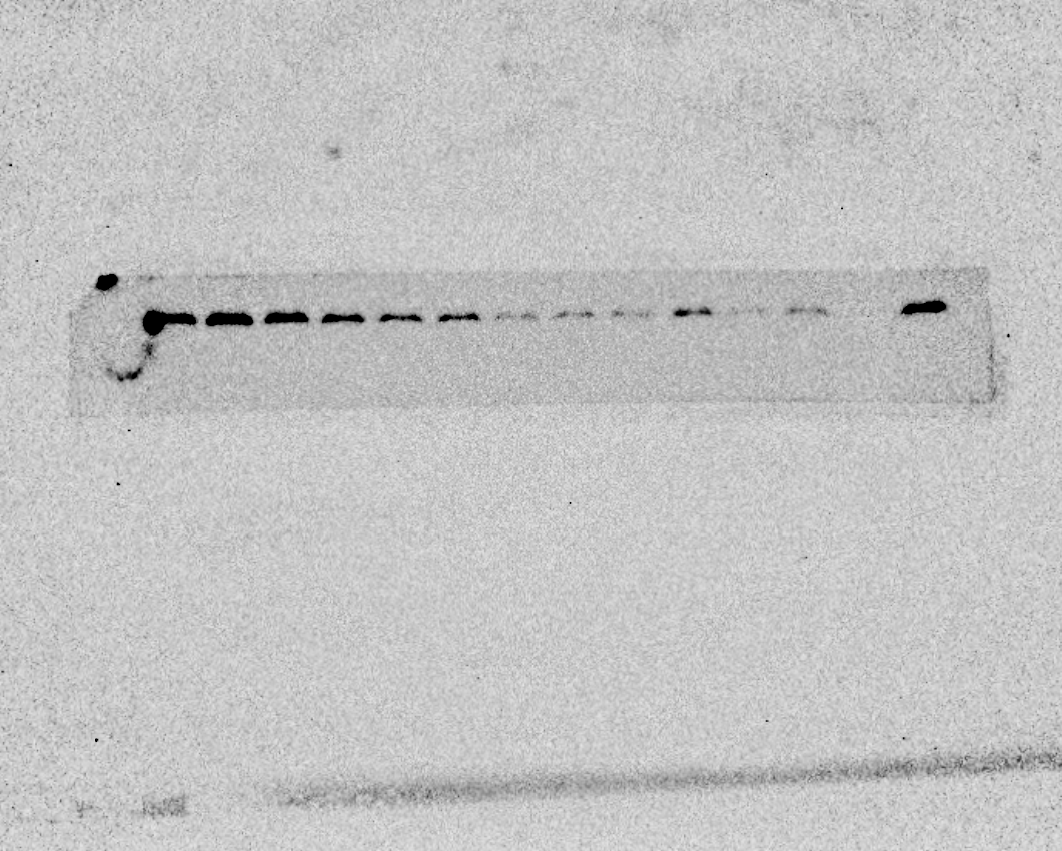

Supplement: Figure 1—source data 2. [file elife-73875-fig1-data2.zip › Figure 1 - source data/Figure 1 panel C/anti-CcrM/anti-CcrM.tif]

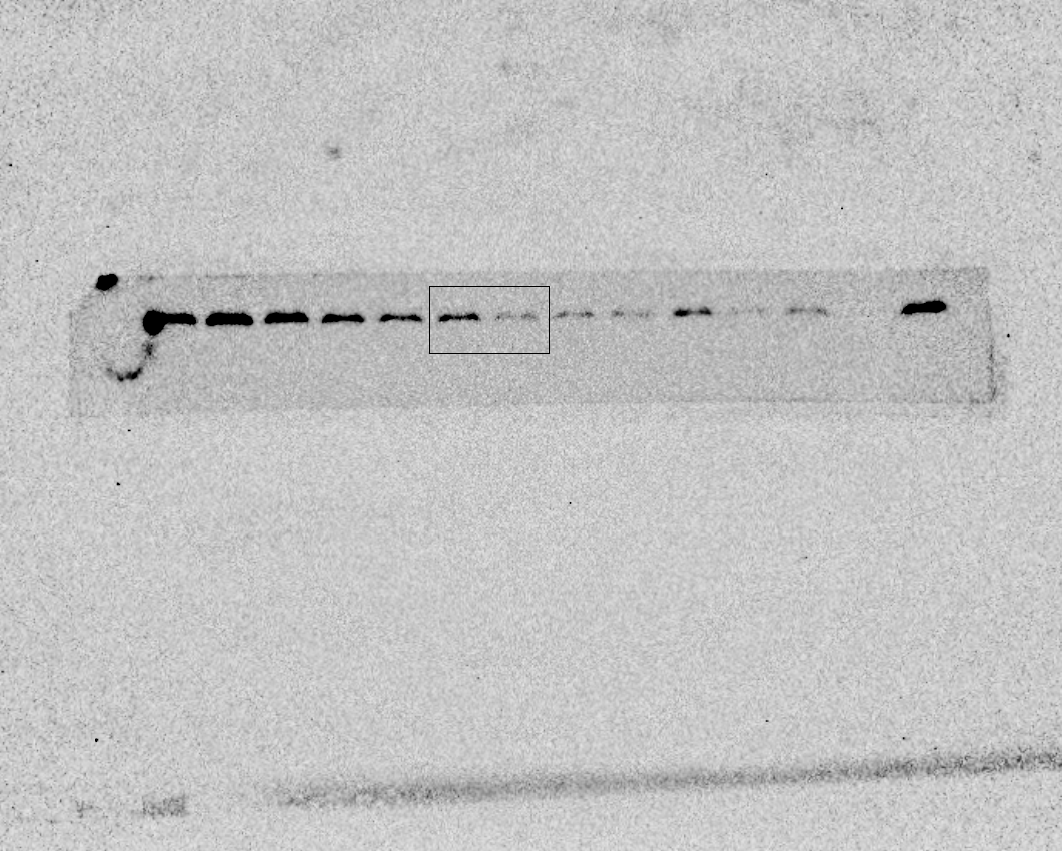

Supplement: Figure 1—source data 2. [file elife-73875-fig1-data2.zip › Figure 1 - source data/Figure 1 panel C/anti-CcrM/anti-CcrM - labelled.tif]

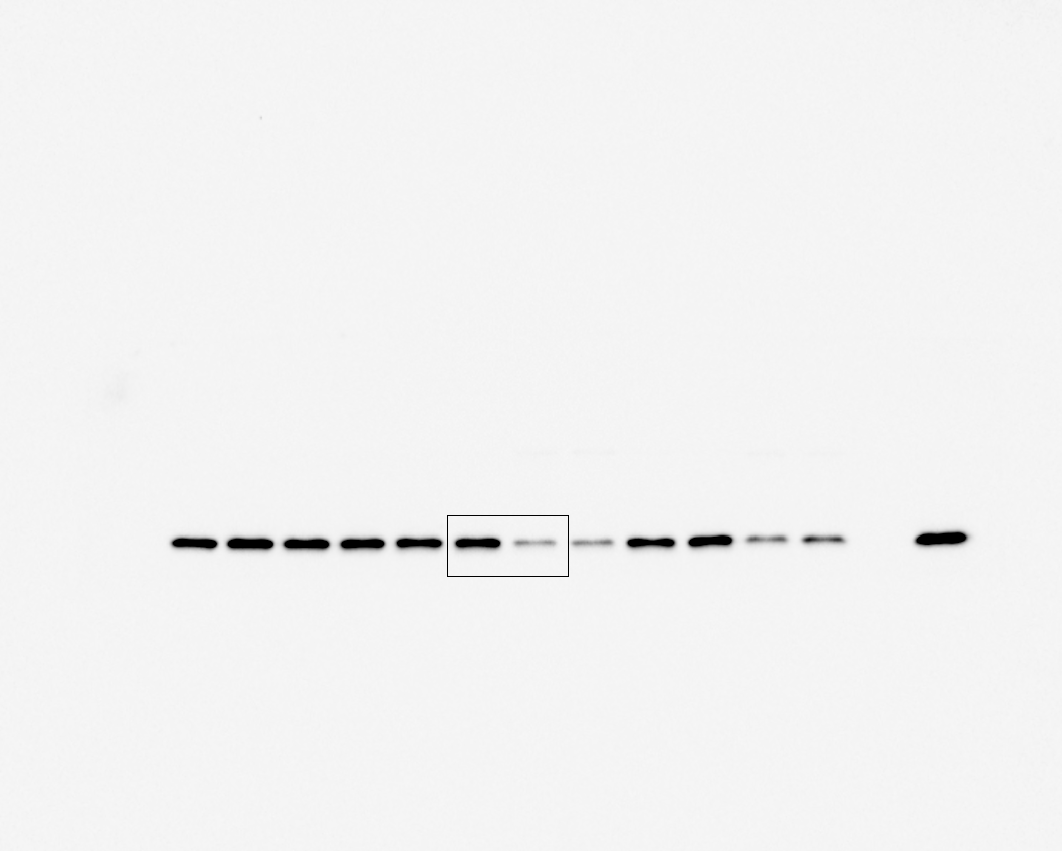

Supplement: Figure 1—source data 2. [file elife-73875-fig1-data2.zip › Figure 1 - source data/Figure 1 panel C/anti-DnaA/anti-DnaA - labelled.tif]

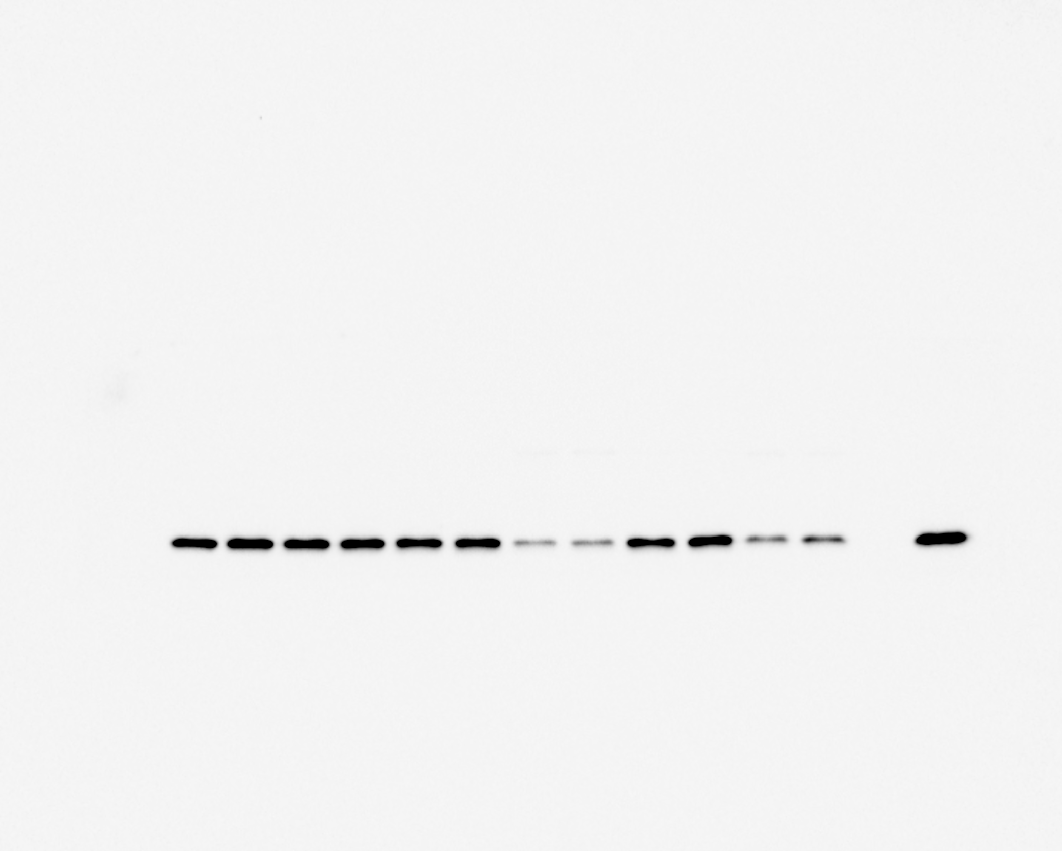

Supplement: Figure 1—source data 2. [file elife-73875-fig1-data2.zip › Figure 1 - source data/Figure 1 panel C/anti-DnaA/anti-DnaA.tif]

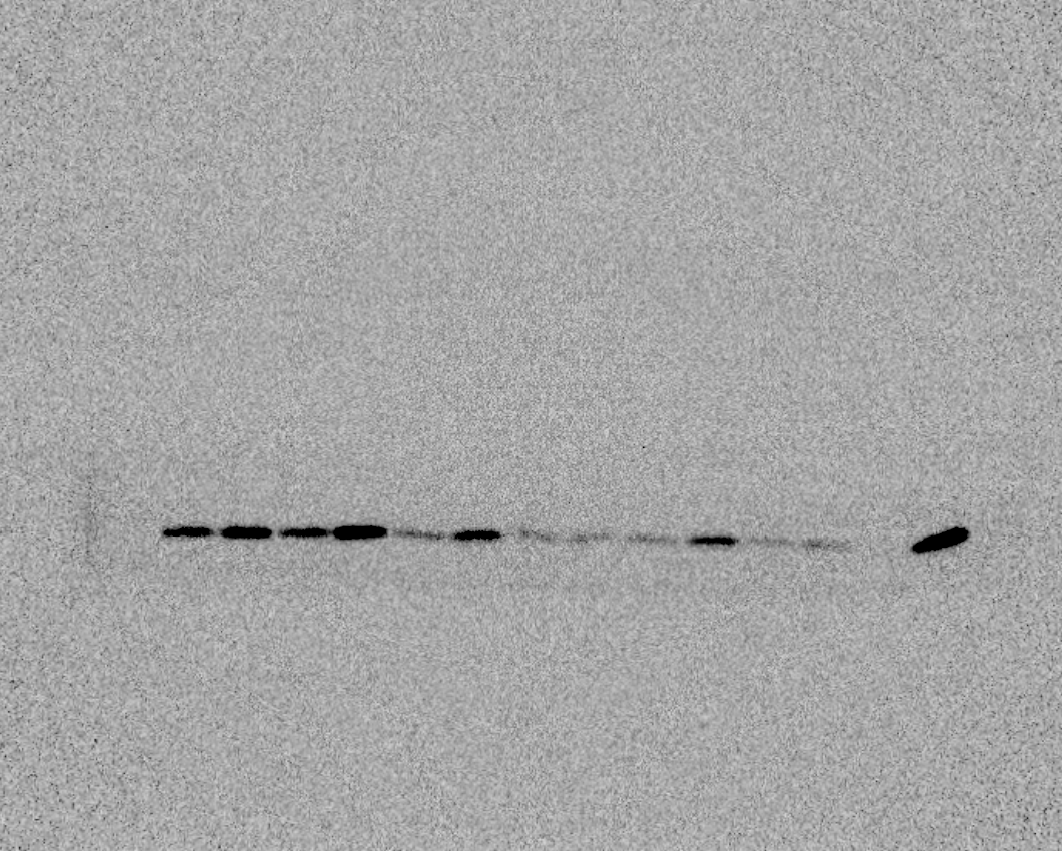

Supplement: Figure 1—source data 2. [file elife-73875-fig1-data2.zip › Figure 1 - source data/Figure 1 panel C/anti-SciP/anti-SciP.tif]

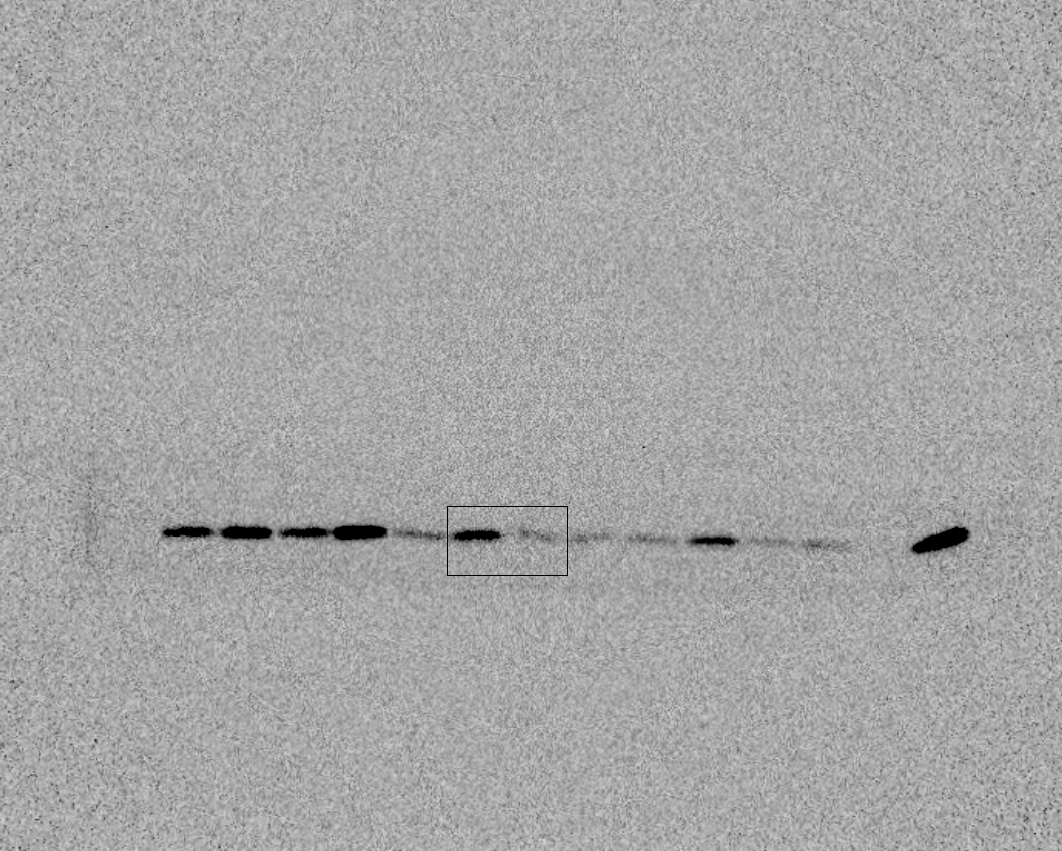

Supplement: Figure 1—source data 2. [file elife-73875-fig1-data2.zip › Figure 1 - source data/Figure 1 panel C/anti-SciP/anti-SciP - labelled.tif]

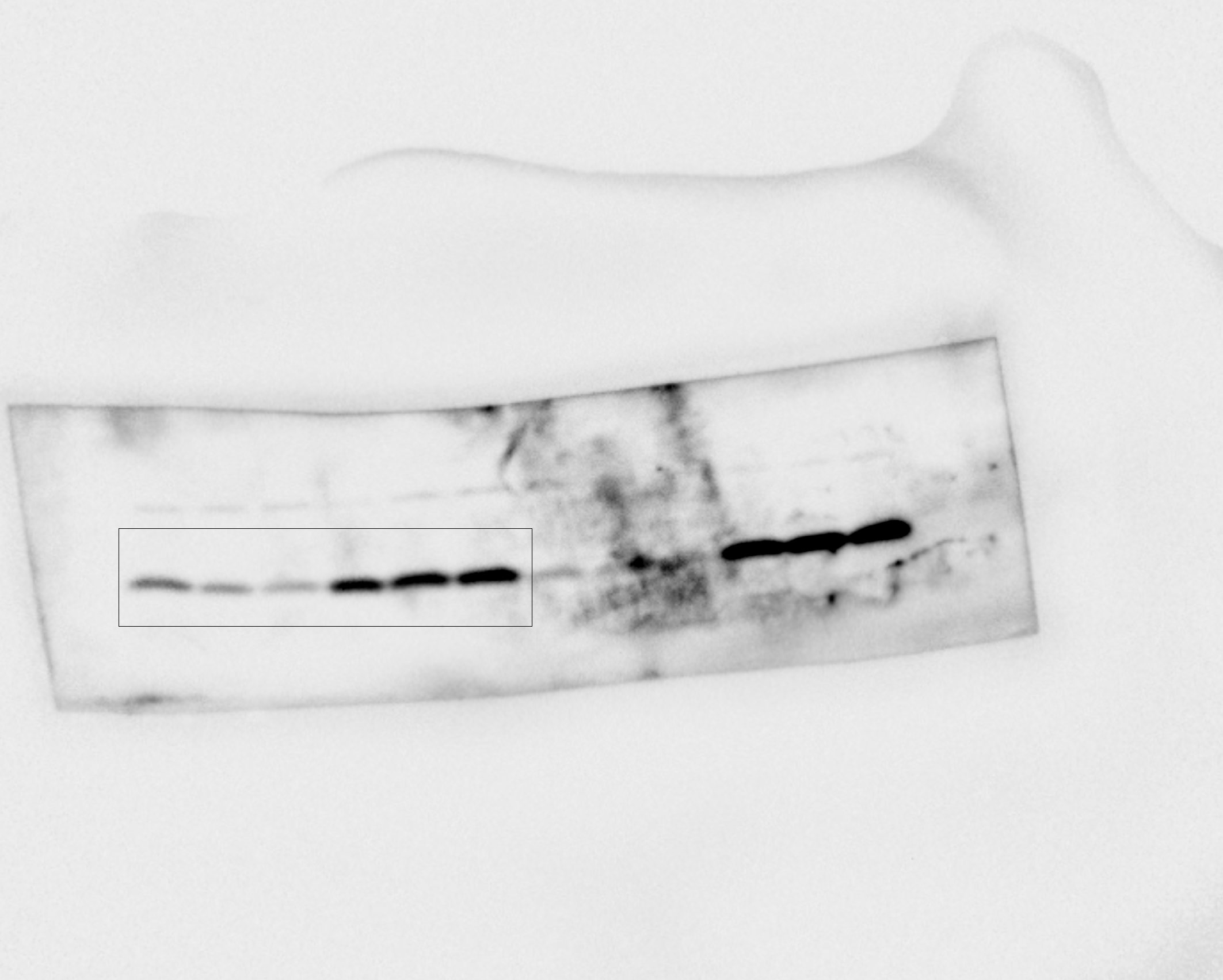

Supplement: Figure 2—source data 1. [file elife-73875-fig2-data1.zip › Figure 2-source data 1/Figure 2 panel C/anti-StaR/anti-StaR - labelled.tif]

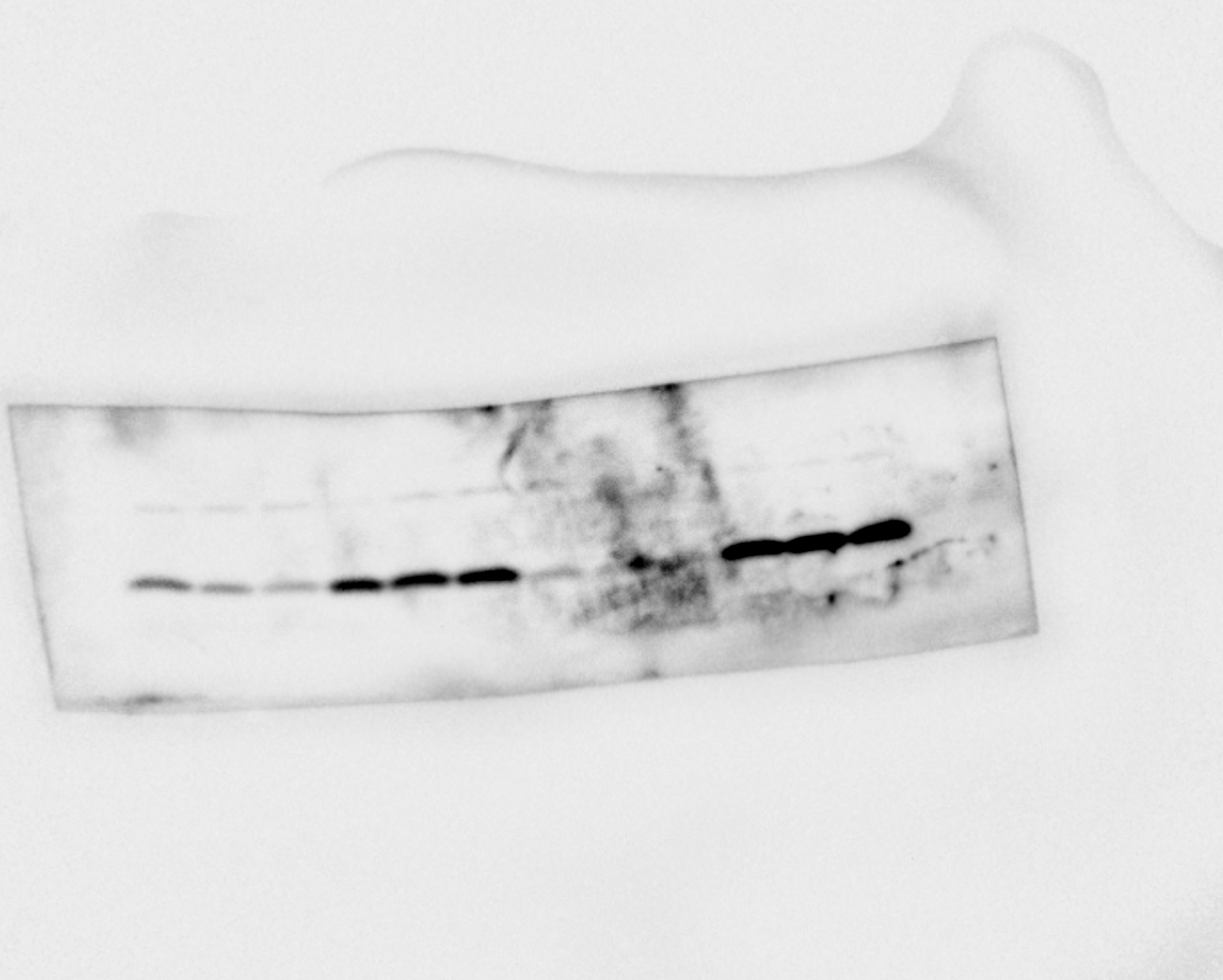

Supplement: Figure 2—source data 1. [file elife-73875-fig2-data1.zip › Figure 2-source data 1/Figure 2 panel C/anti-StaR/anti-StaR.tif]

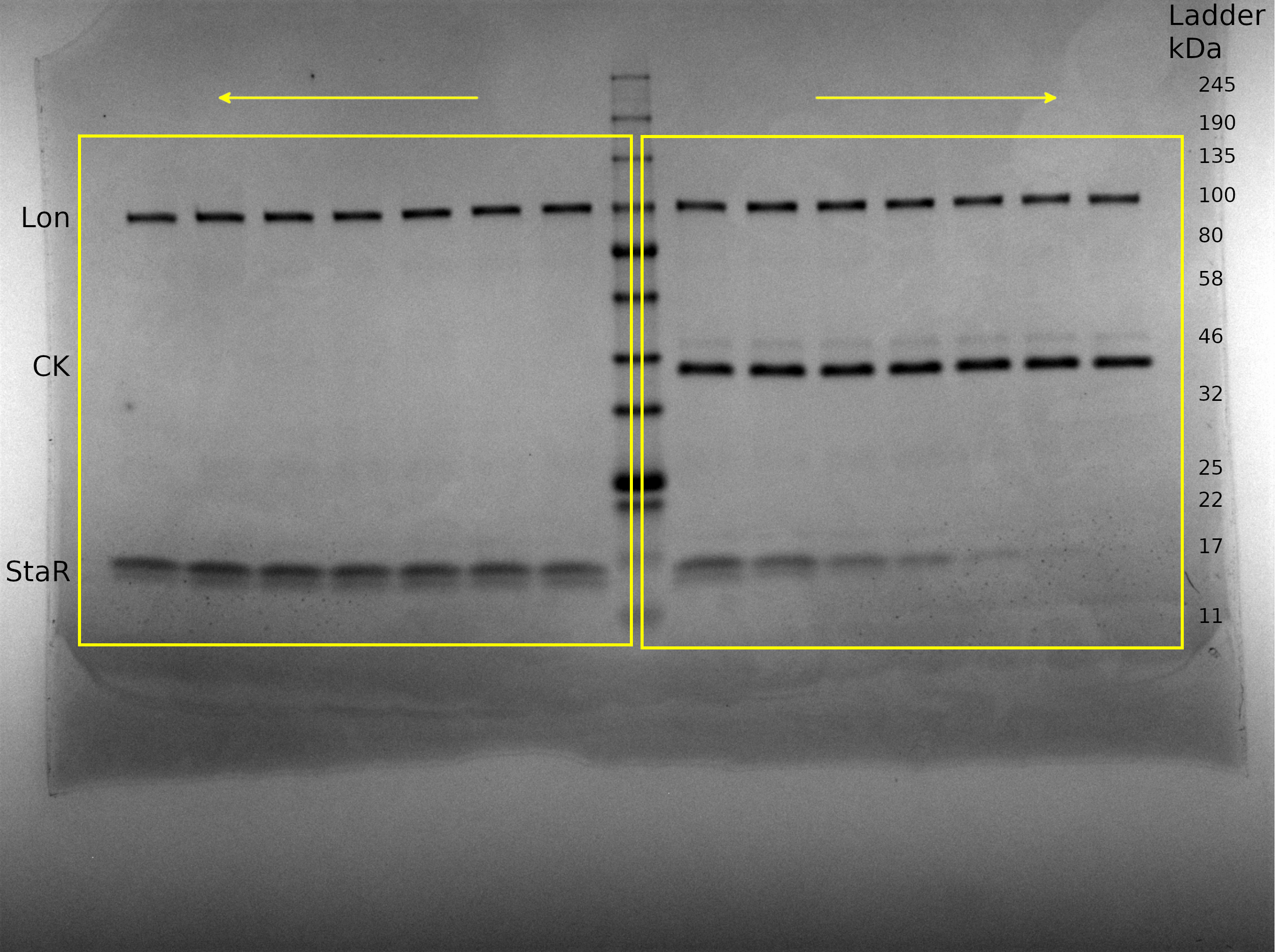

Supplement: Figure 2—source data 1. [file elife-73875-fig2-data1.zip › Figure 2-source data 1/Figure 2 panel D/iv deg StaR/Lon+StaR+-ATP - crop_labelled.tif]

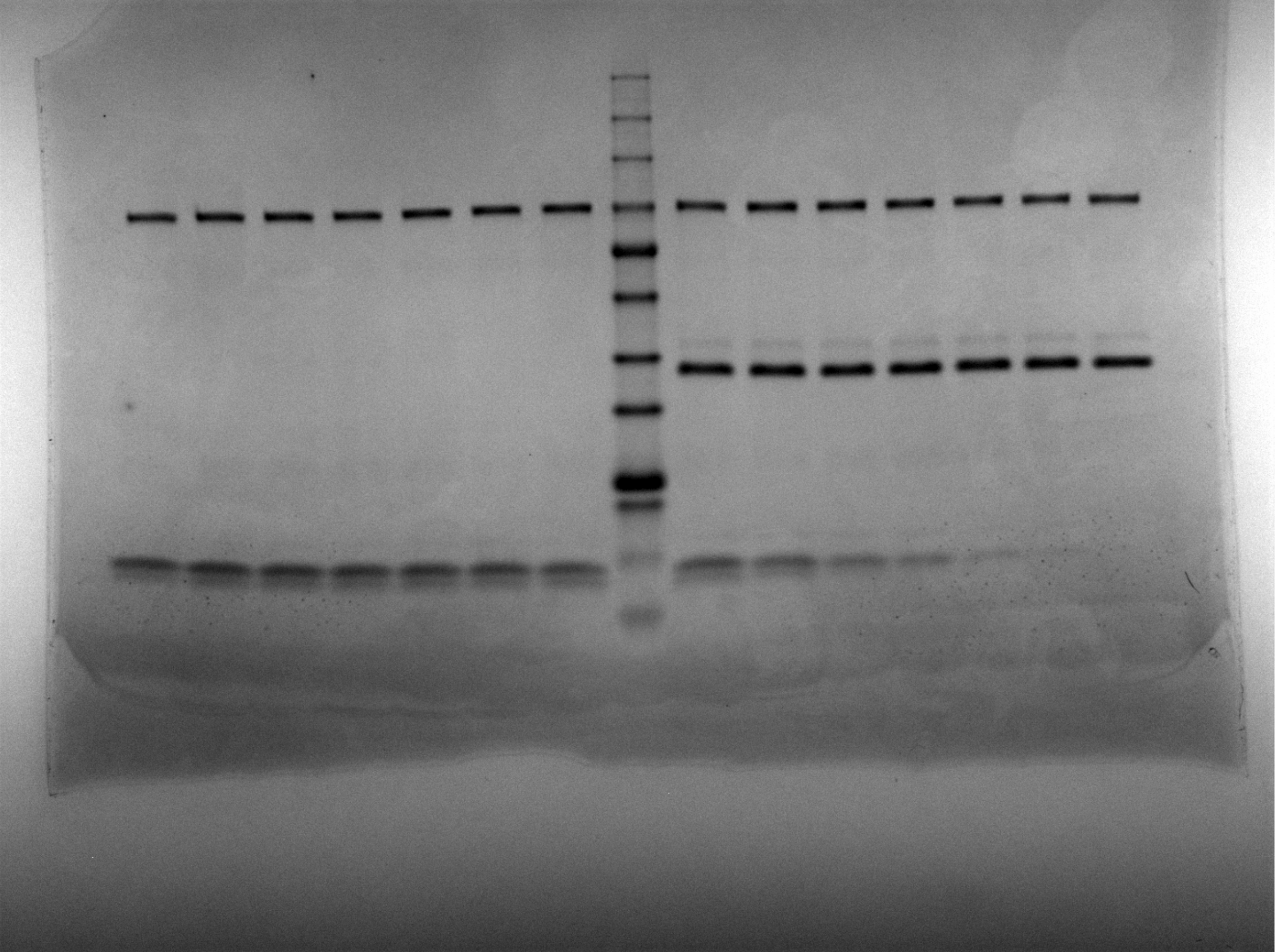

Supplement: Figure 2—source data 1. [file elife-73875-fig2-data1.zip › Figure 2-source data 1/Figure 2 panel D/iv deg StaR/Lon+StaR+-ATP.tif]

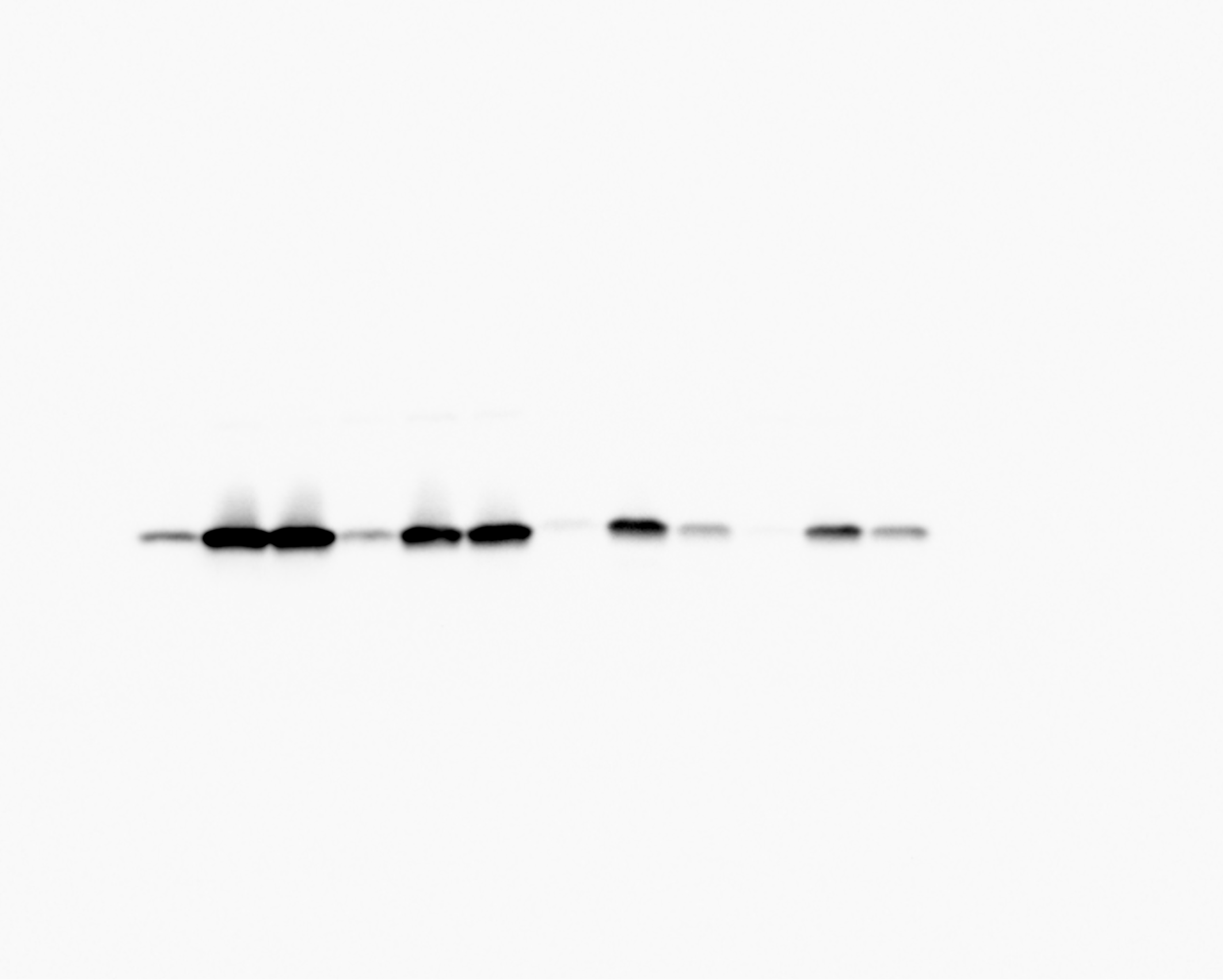

Supplement: Figure 2—source data 1. [file elife-73875-fig2-data1.zip › Figure 2-source data 1/Figure 2 panel E/anti-FLAG/anti-FLAG.tif]

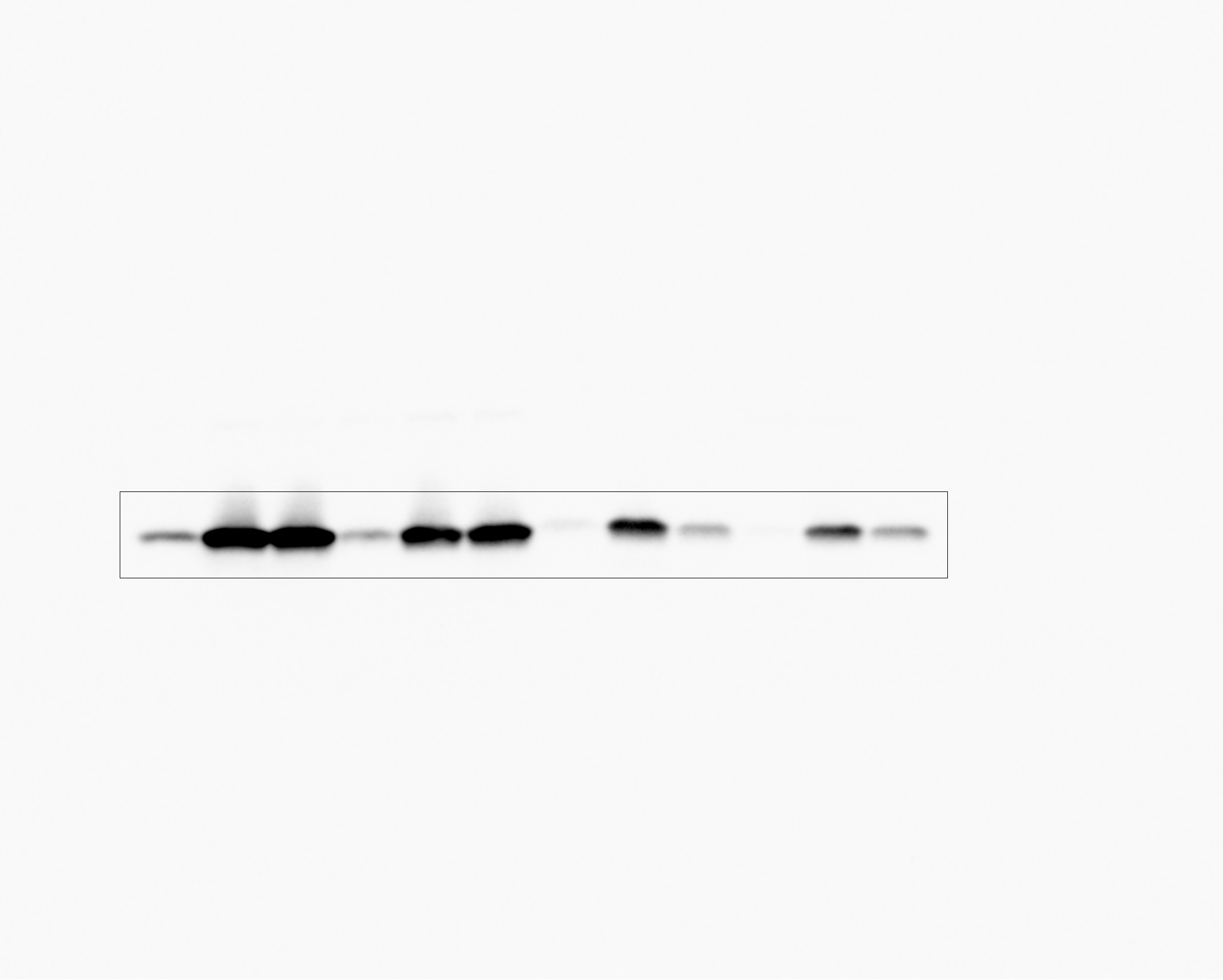

Supplement: Figure 2—source data 1. [file elife-73875-fig2-data1.zip › Figure 2-source data 1/Figure 2 panel E/anti-FLAG/anti-FLAG - labelled.tif]

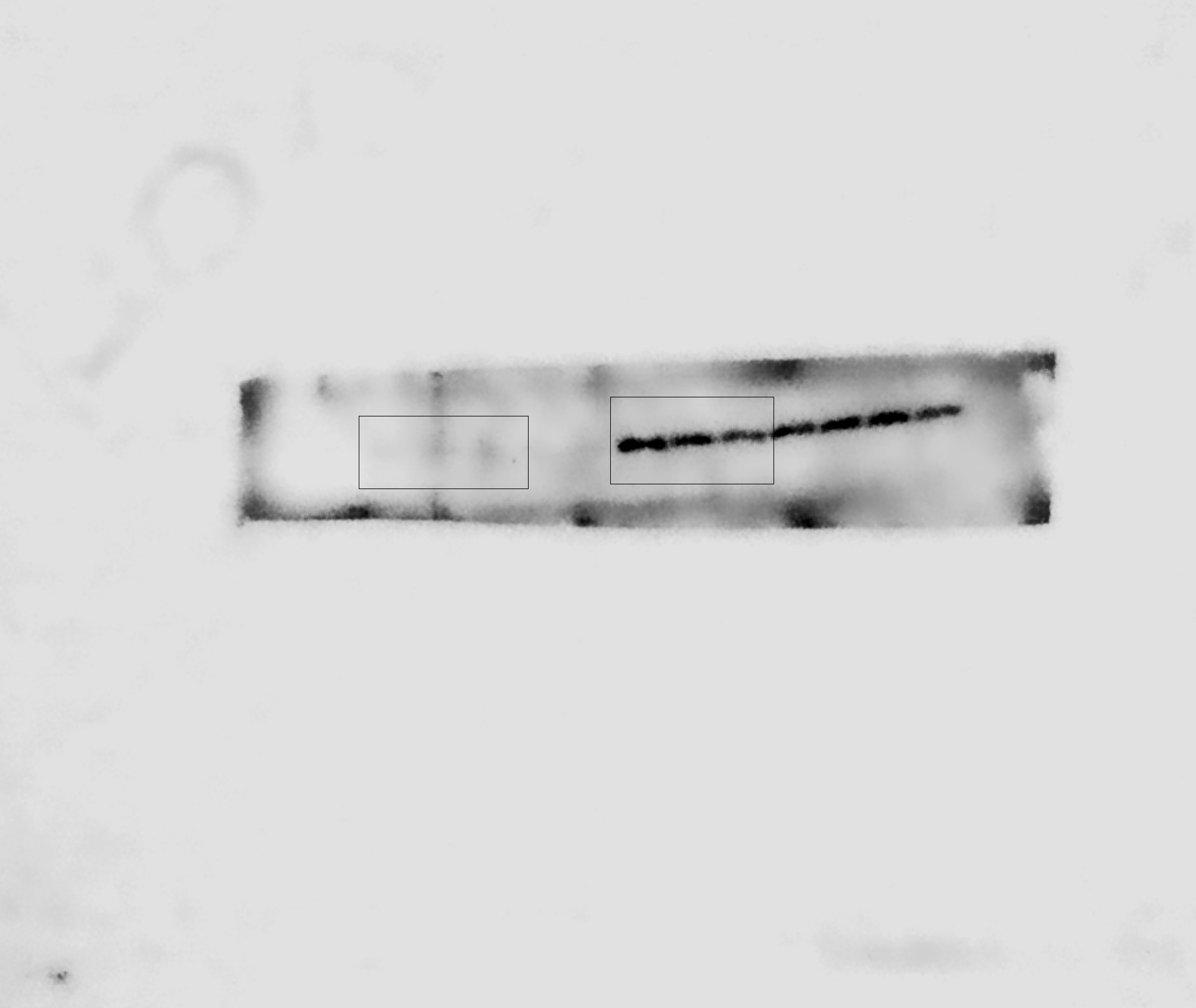

Supplement: Figure 2—source data 1. [file elife-73875-fig2-data1.zip › Figure 2-source data 1/Figure 2 panel B/anti-StaR/anti-StaR - labelled.tif]

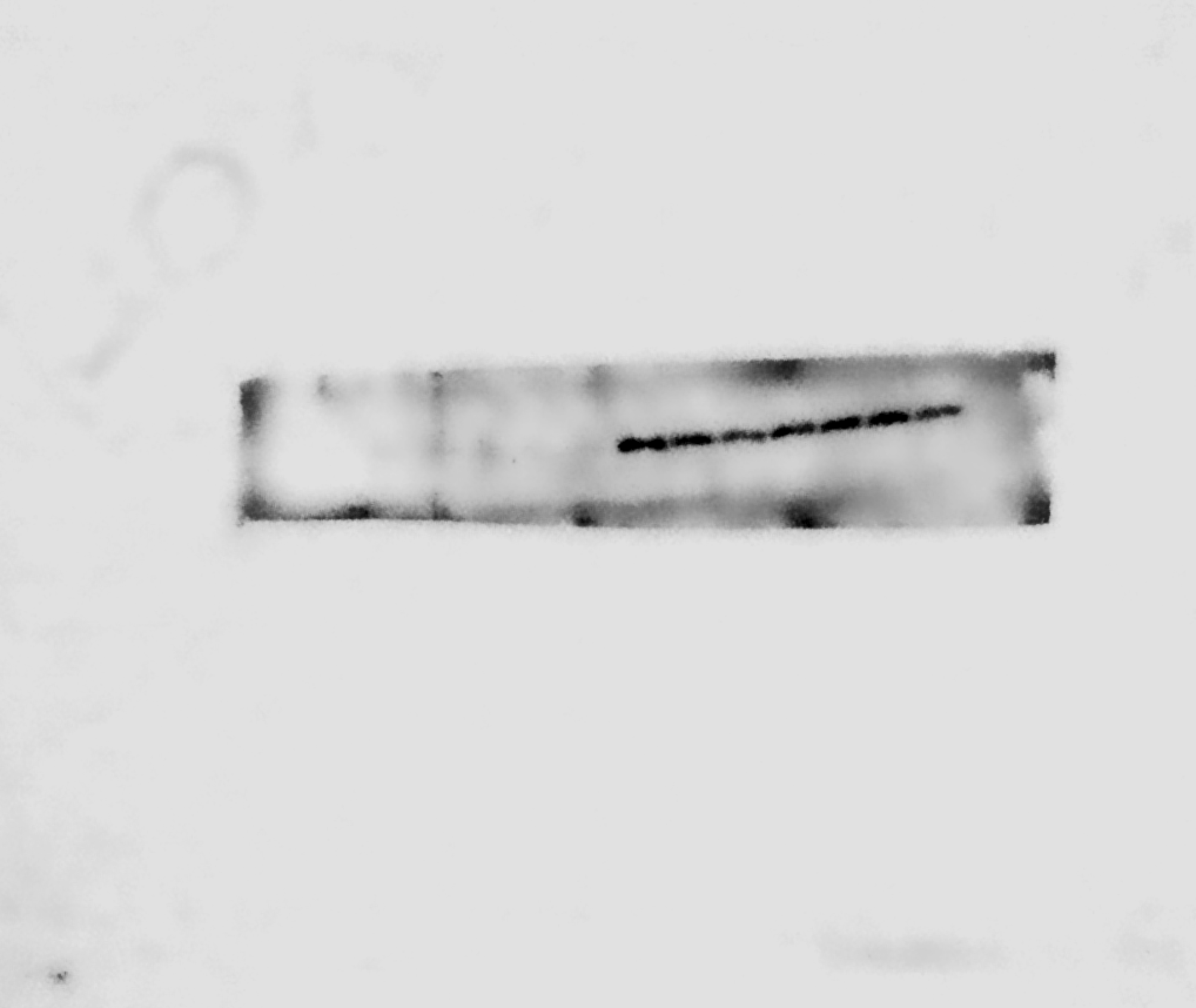

Supplement: Figure 2—source data 1. [file elife-73875-fig2-data1.zip › Figure 2-source data 1/Figure 2 panel B/anti-StaR/anti-StaR.tif]

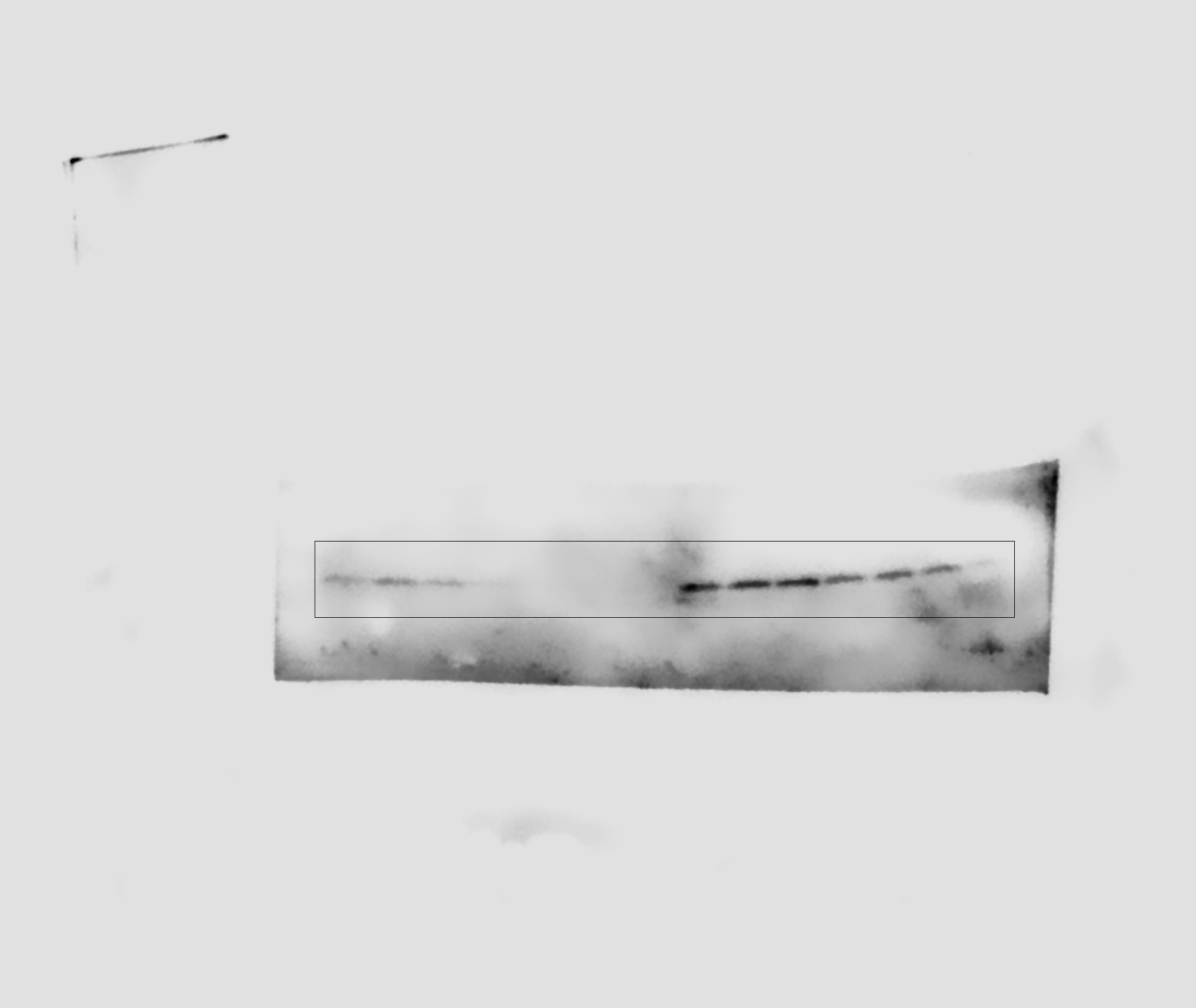

Supplement: Figure 3—source data 1. [file elife-73875-fig3-data1.zip › Figure 3-source data 1/Figure 3 panel C/anti-StaR/anti-StaR - labelled.tif]

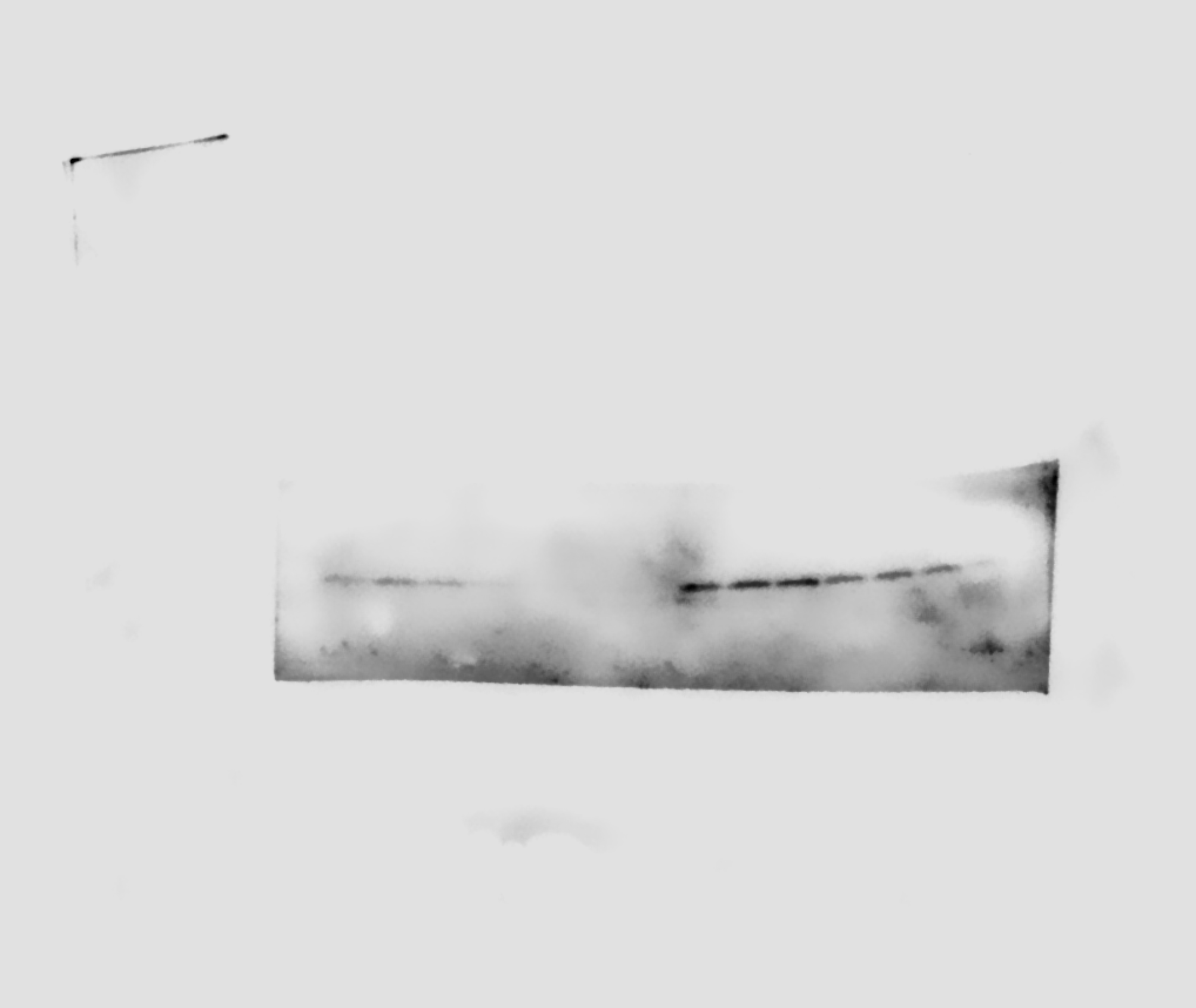

Supplement: Figure 3—source data 1. [file elife-73875-fig3-data1.zip › Figure 3-source data 1/Figure 3 panel C/anti-StaR/anti-StaR.tif]

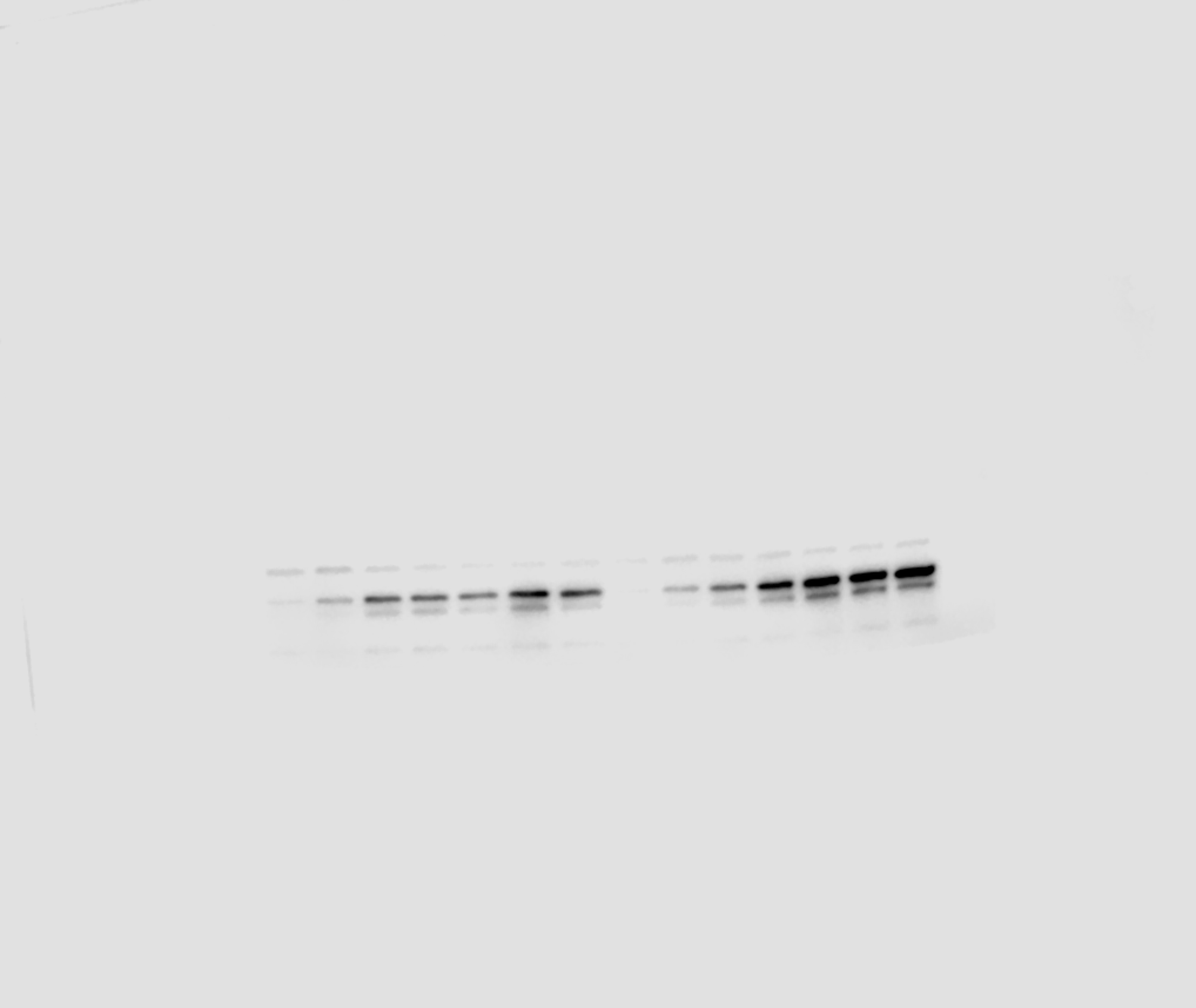

Supplement: Figure 3—source data 1. [file elife-73875-fig3-data1.zip › Figure 3-source data 1/Figure 3 panel C/anti-TipF/anti-TipF.tif]

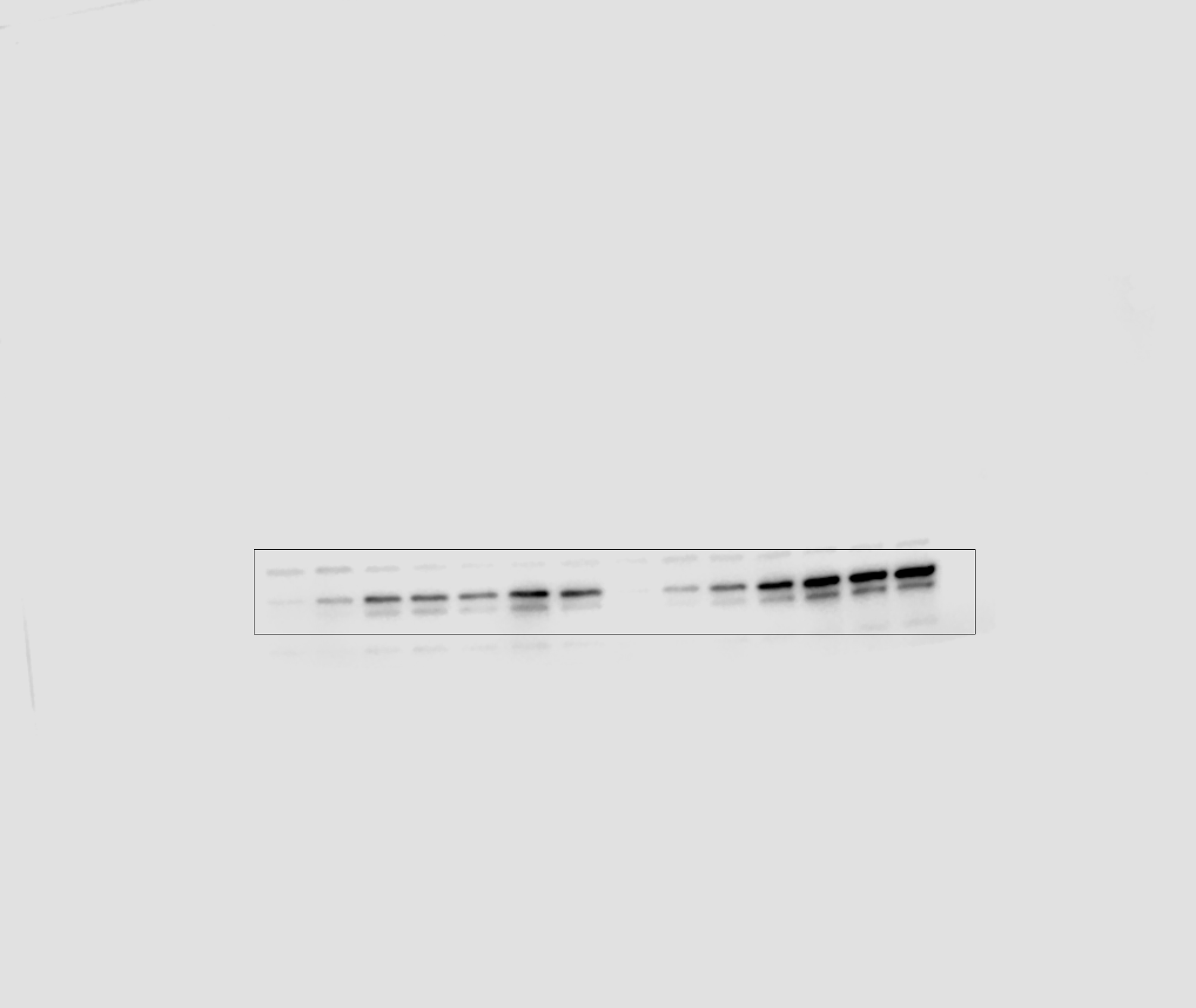

Supplement: Figure 3—source data 1. [file elife-73875-fig3-data1.zip › Figure 3-source data 1/Figure 3 panel C/anti-TipF/anti-TipF - labelled.tif]

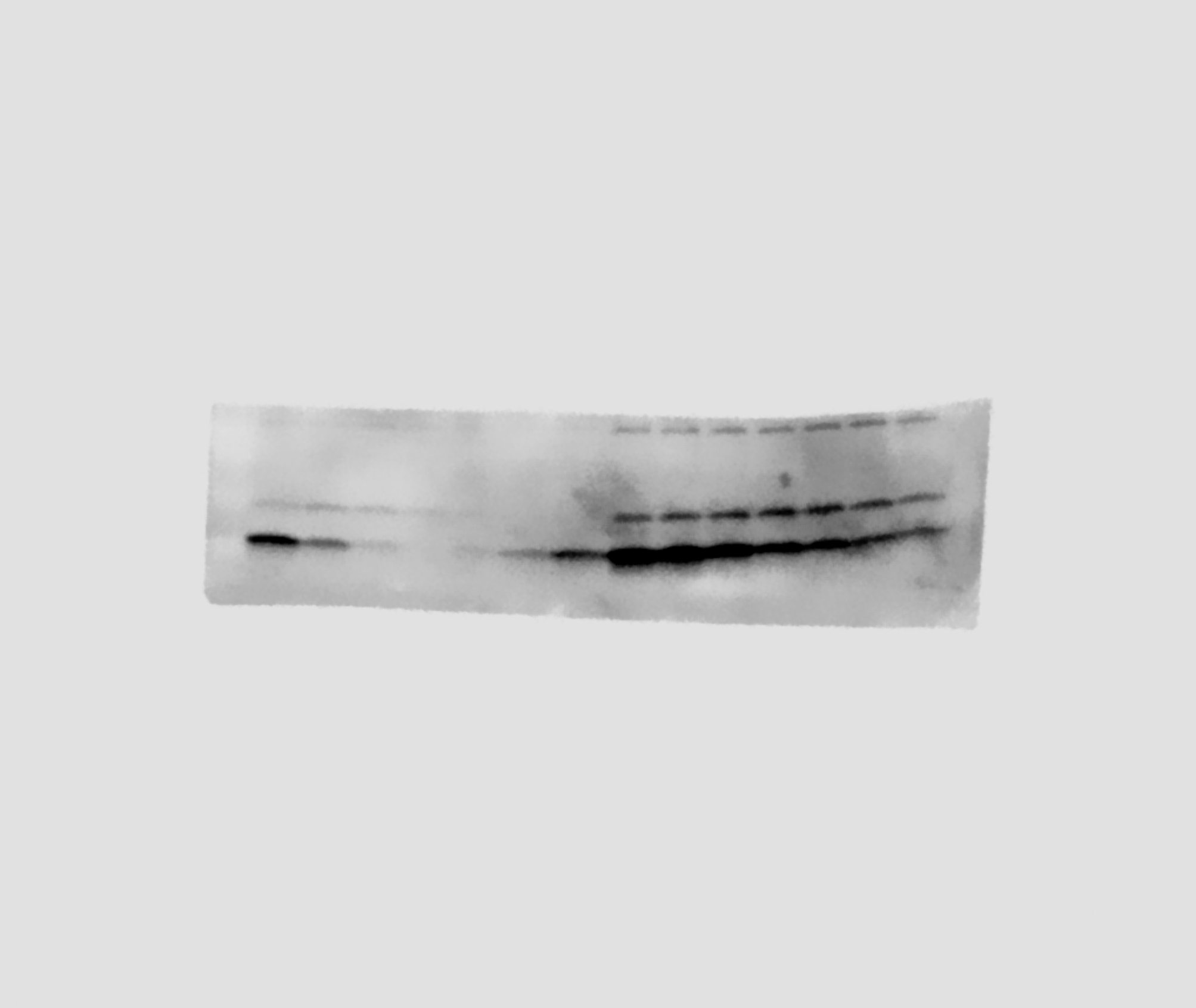

Supplement: Figure 3—source data 1. [file elife-73875-fig3-data1.zip › Figure 3-source data 1/Figure 3 panel C/anti-SciP/anti-SciP.tif]

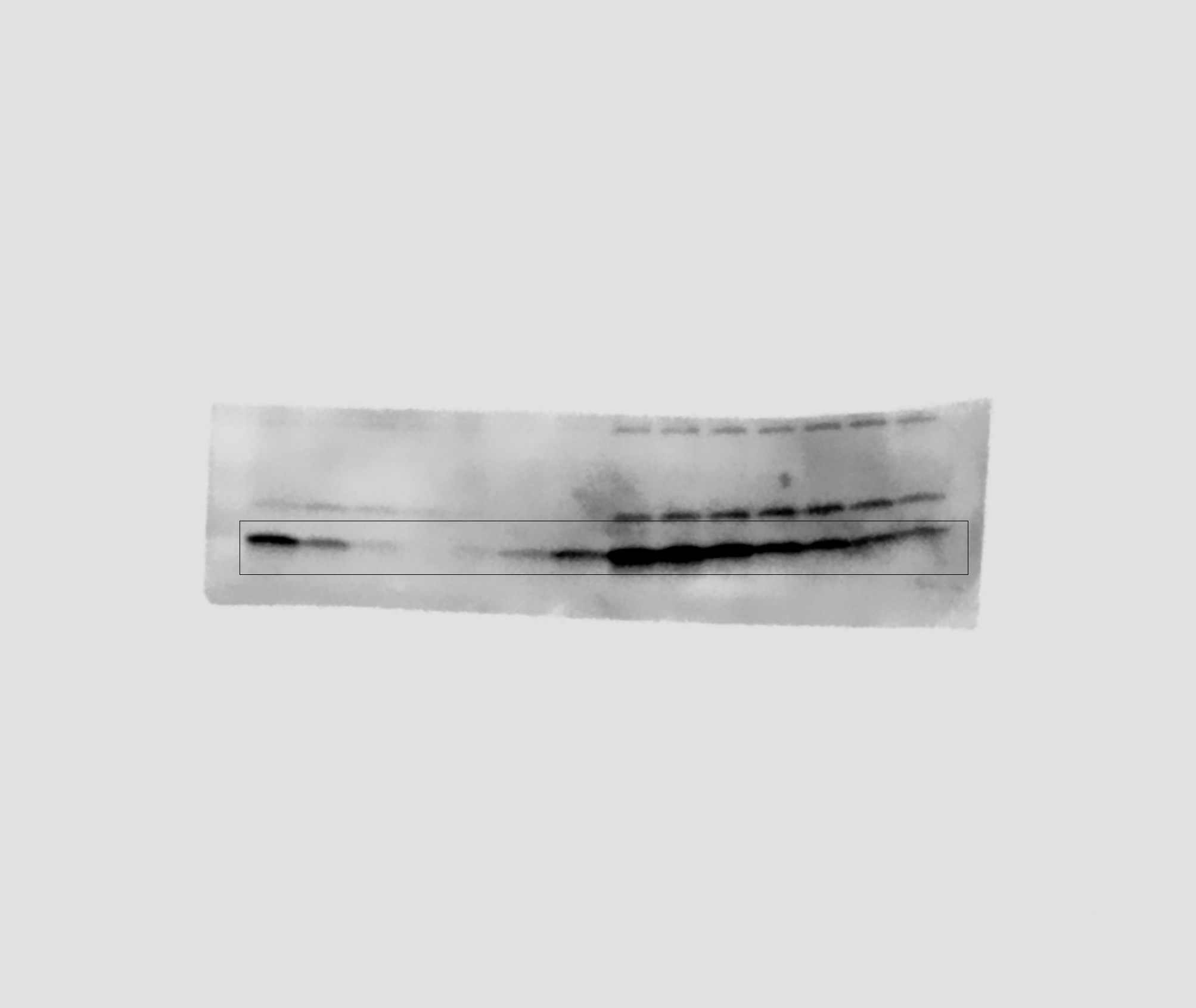

Supplement: Figure 3—source data 1. [file elife-73875-fig3-data1.zip › Figure 3-source data 1/Figure 3 panel C/anti-SciP/anti-SciP - labelled.tif]

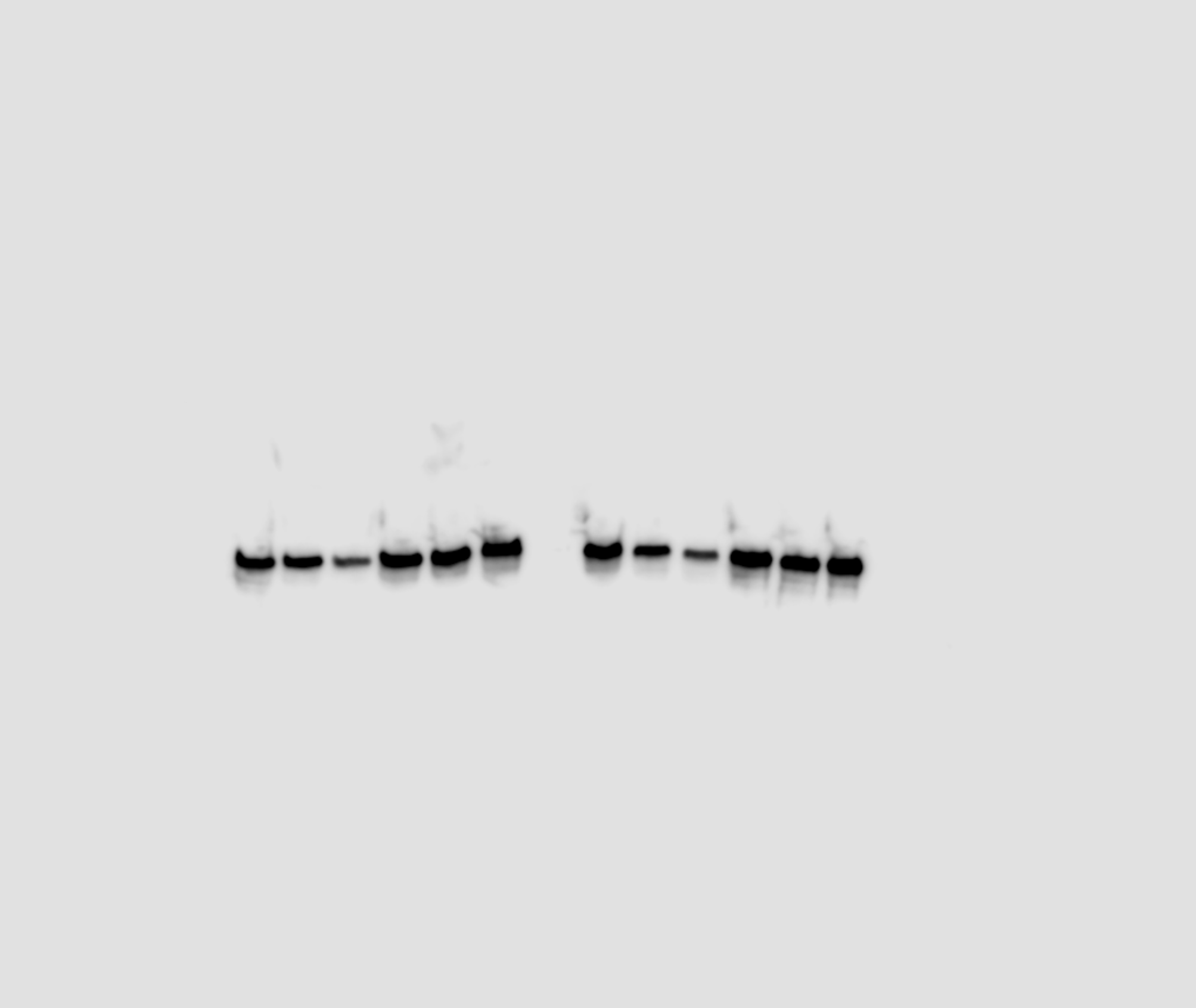

Supplement: Figure 4—source data 1. [file elife-73875-fig4-data1.zip › Figure 4-source data 1 /Figure 4 panel D/anti-FLAG/anti-FLAG.tif]

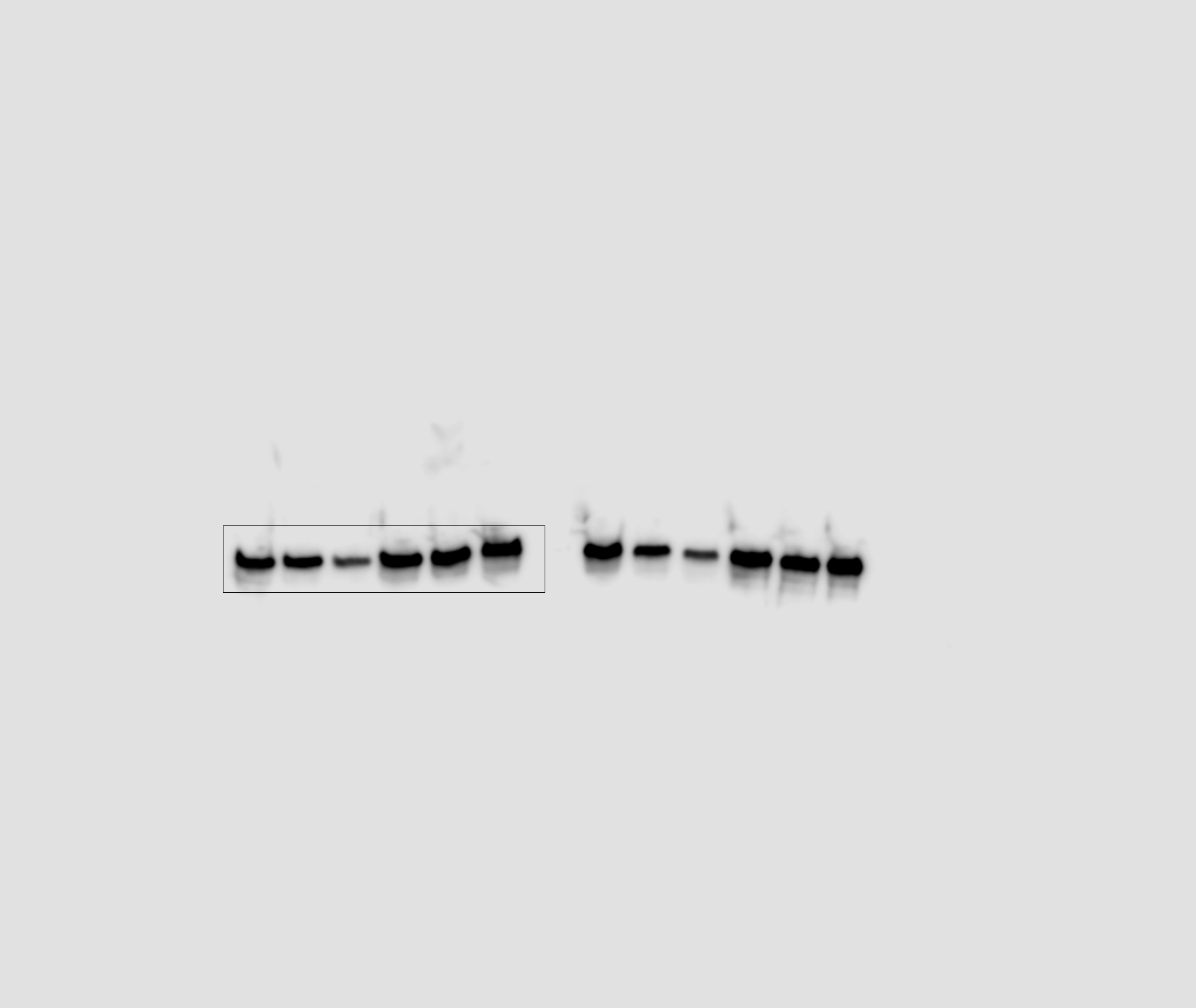

Supplement: Figure 4—source data 1. [file elife-73875-fig4-data1.zip › Figure 4-source data 1 /Figure 4 panel D/anti-FLAG/anti-FLAG- labelled.tif]

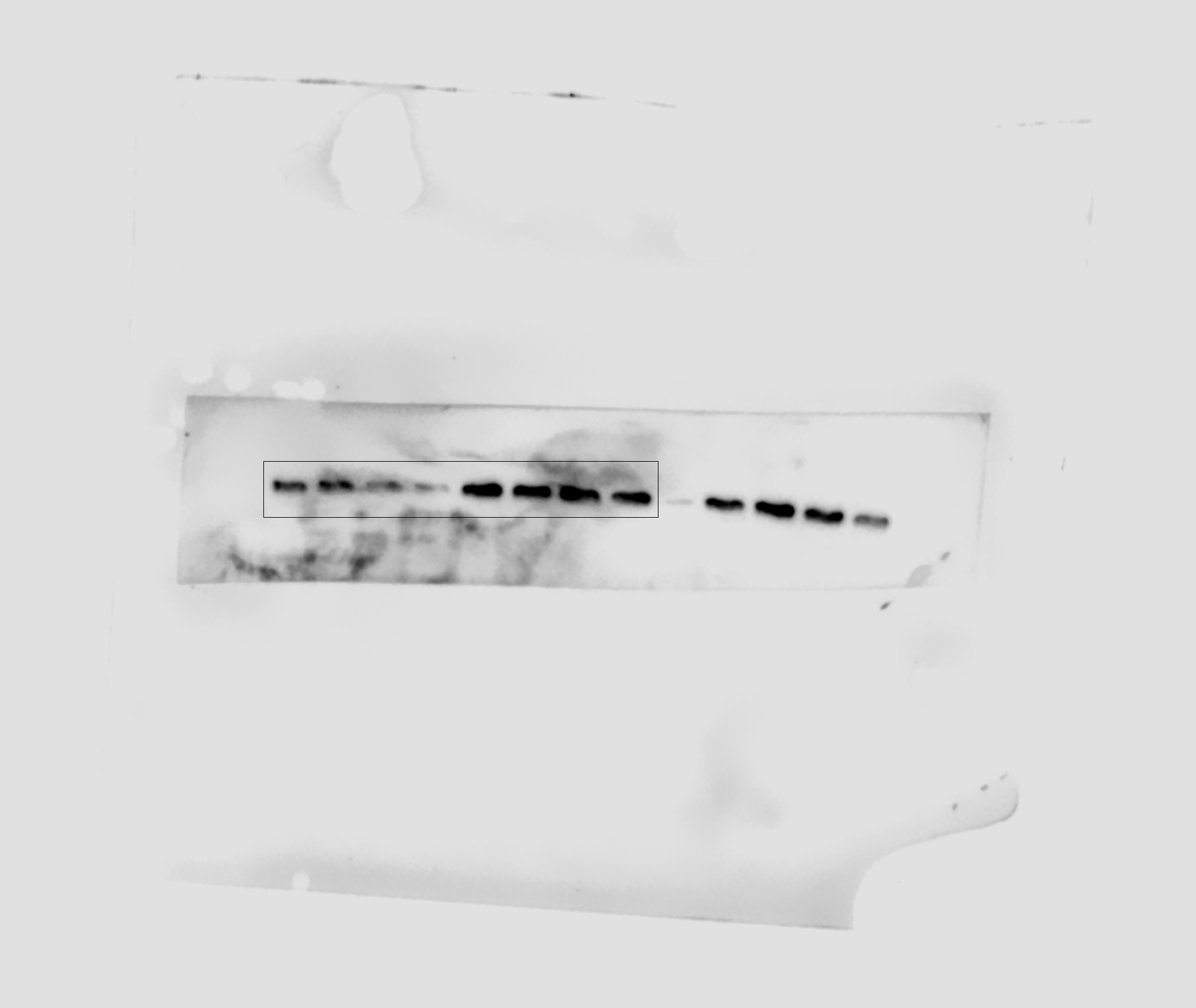

Supplement: Figure 4—source data 1. [file elife-73875-fig4-data1.zip › Figure 4-source data 1 /Figure 4 panel C/anti-FliK-C/anti-FliK-C - labelled.tif]

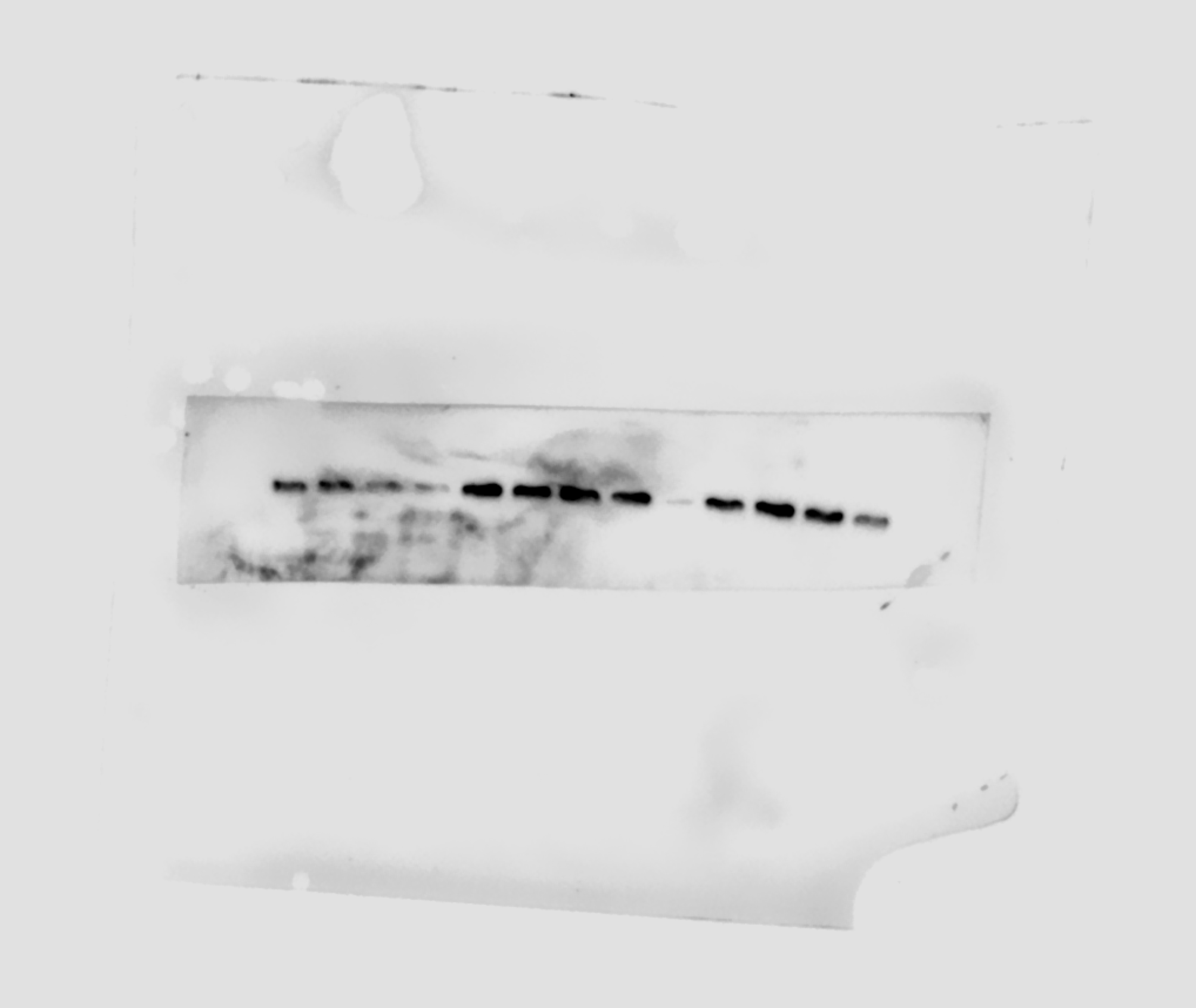

Supplement: Figure 4—source data 1. [file elife-73875-fig4-data1.zip › Figure 4-source data 1 /Figure 4 panel C/anti-FliK-C/anti-FliK-C.tif]

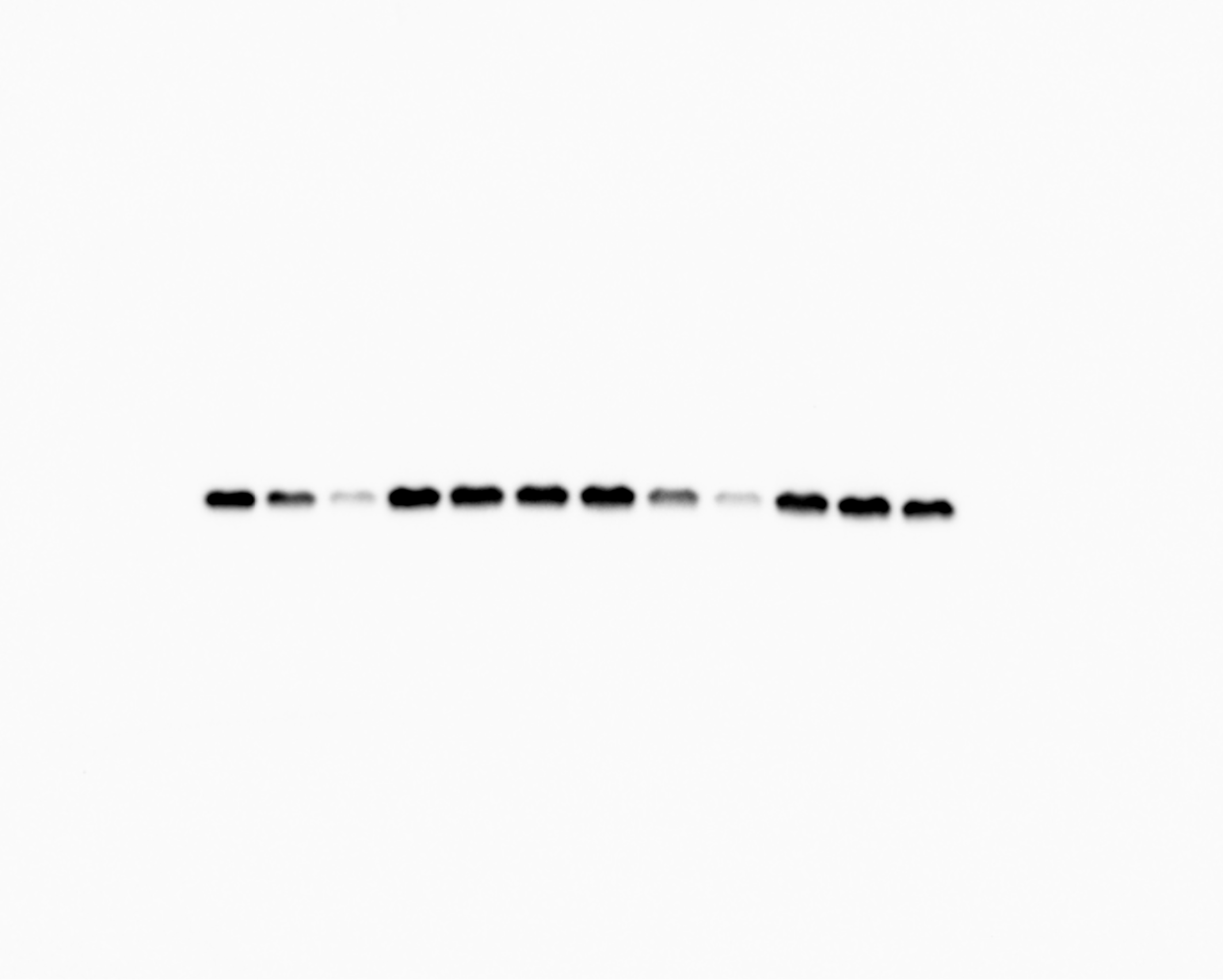

Supplement: Figure 4—source data 1. [file elife-73875-fig4-data1.zip › Figure 4-source data 1 /Figure 4 panel E/anti-FLAG/anti-FLAG.tif]

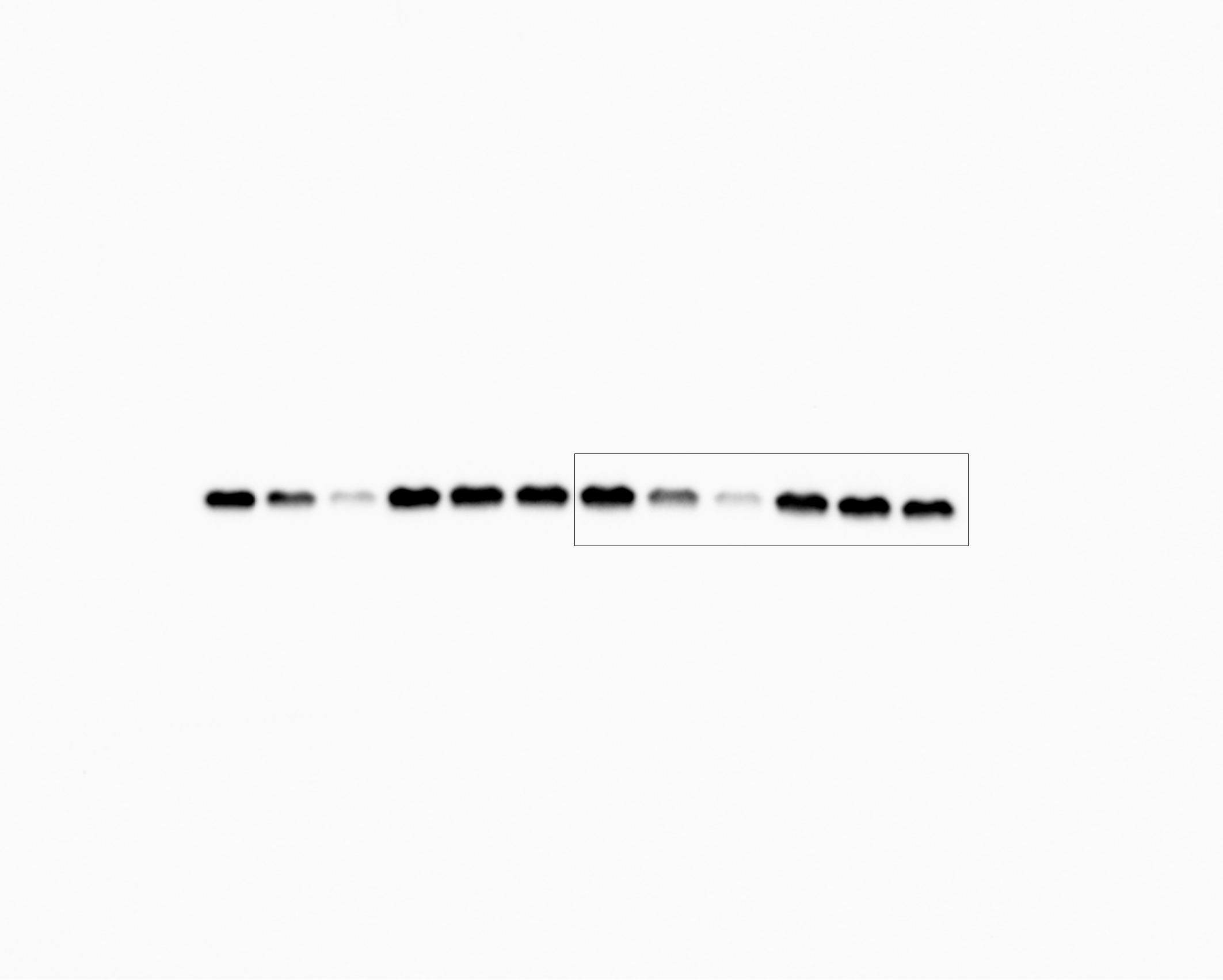

Supplement: Figure 4—source data 1. [file elife-73875-fig4-data1.zip › Figure 4-source data 1 /Figure 4 panel E/anti-FLAG/anti-FLAG - labelled.tif]

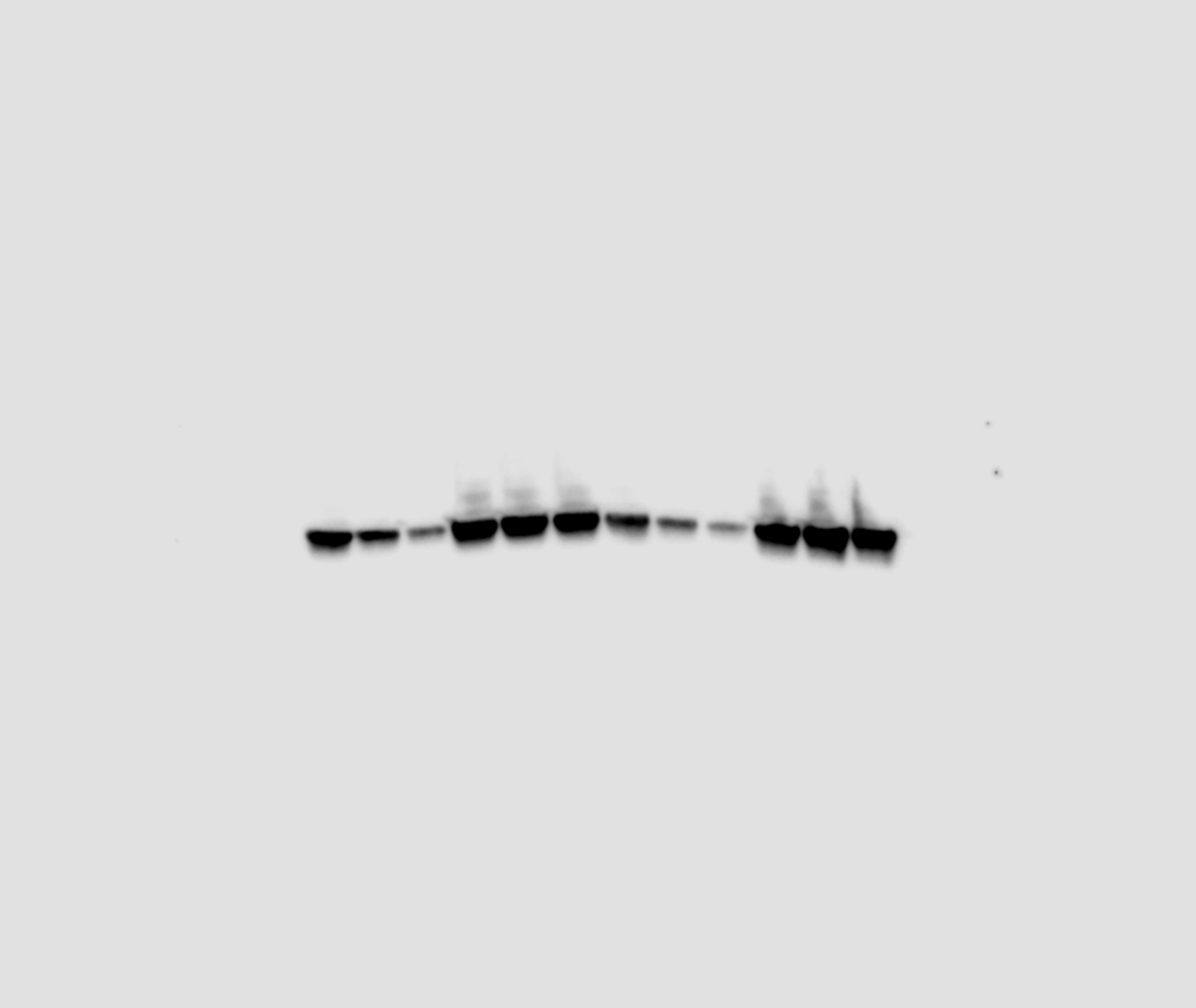

Supplement: Figure 4—source data 1. [file elife-73875-fig4-data1.zip › Figure 4-source data 1 /Figure 4 panel G/anti-FLAG/anti-FLAG.tif]

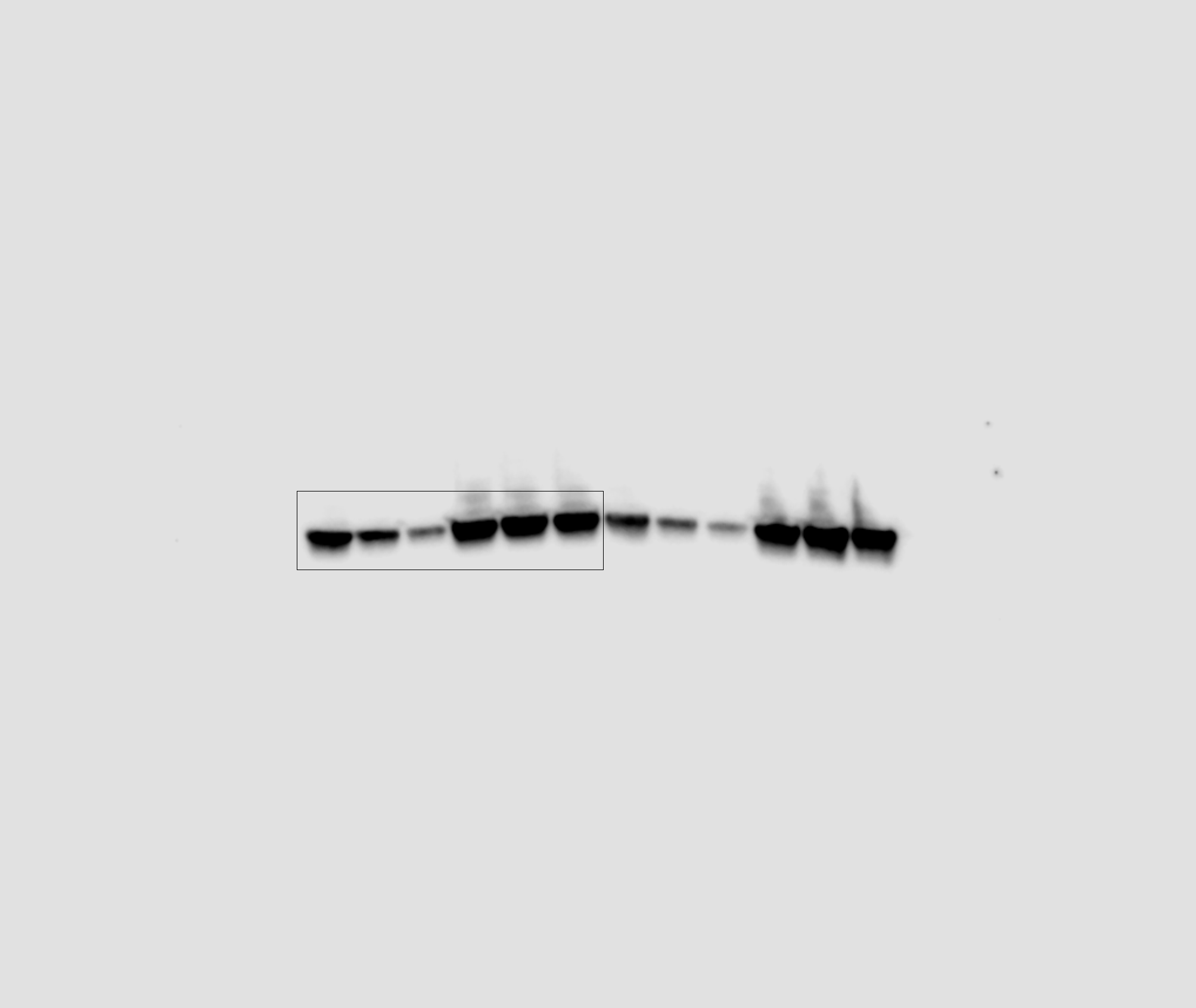

Supplement: Figure 4—source data 1. [file elife-73875-fig4-data1.zip › Figure 4-source data 1 /Figure 4 panel G/anti-FLAG/anti-FLAG - labelled.tif]

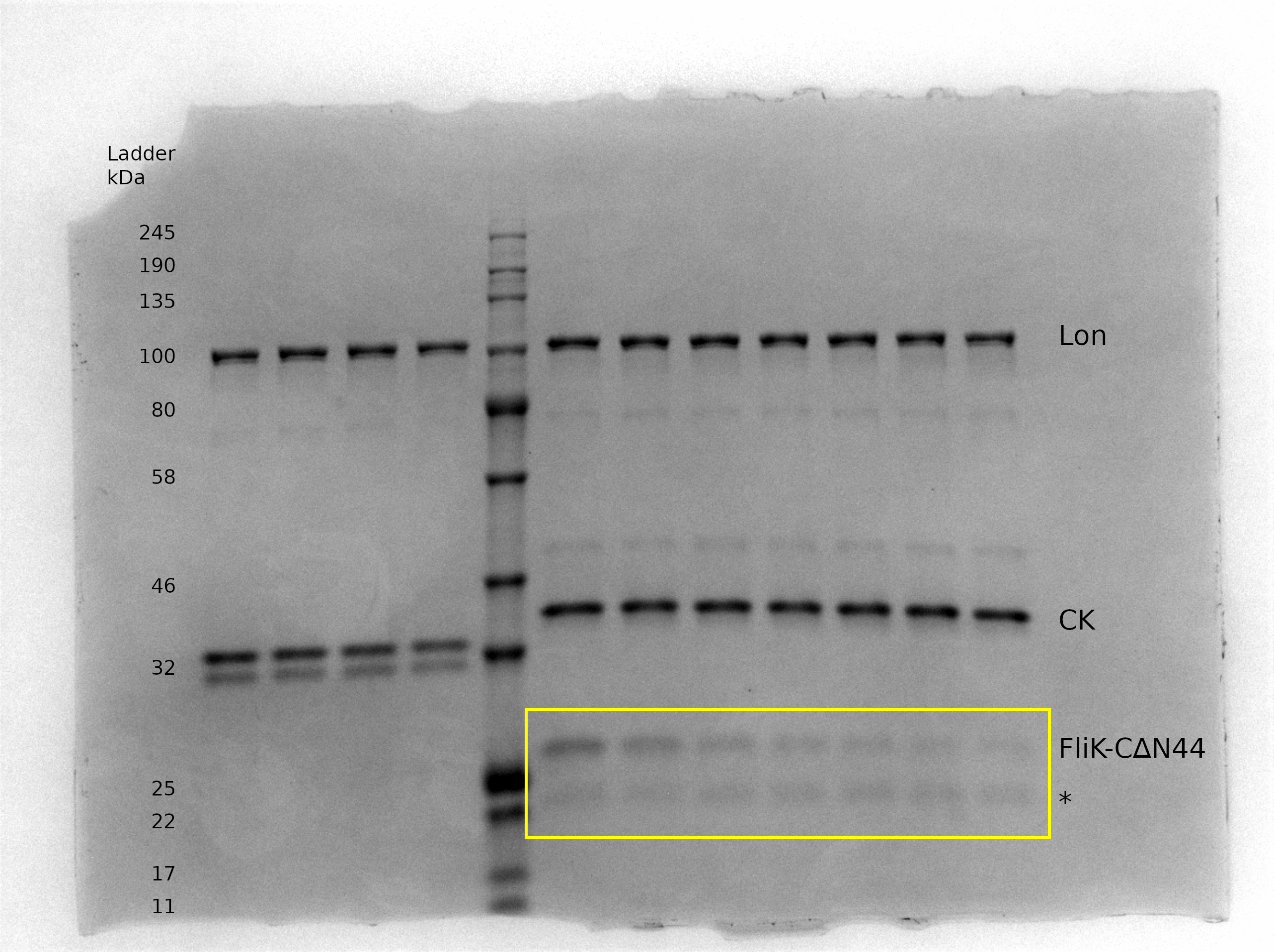

Supplement: Figure 4—source data 1. [file elife-73875-fig4-data1.zip › Figure 4-source data 1 /Figure 4 panel F/iv deg FliK-C +trunc/FliK-C dN44 - Crop_labelled.tif]

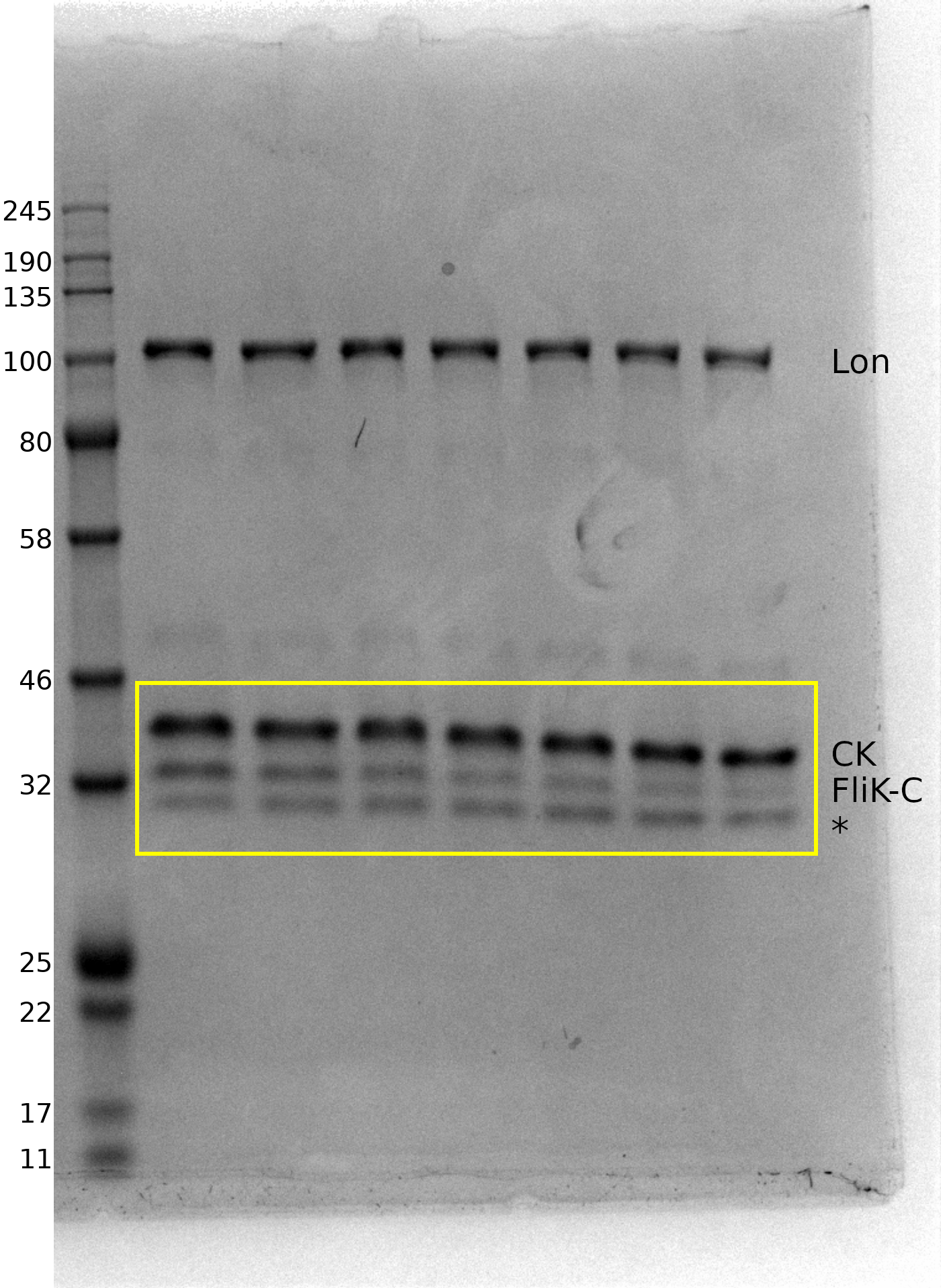

Supplement: Figure 4—source data 1. [file elife-73875-fig4-data1.zip › Figure 4-source data 1 /Figure 4 panel F/iv deg FliK-C +trunc/FliK-C wt - Crop_labelled.tif]

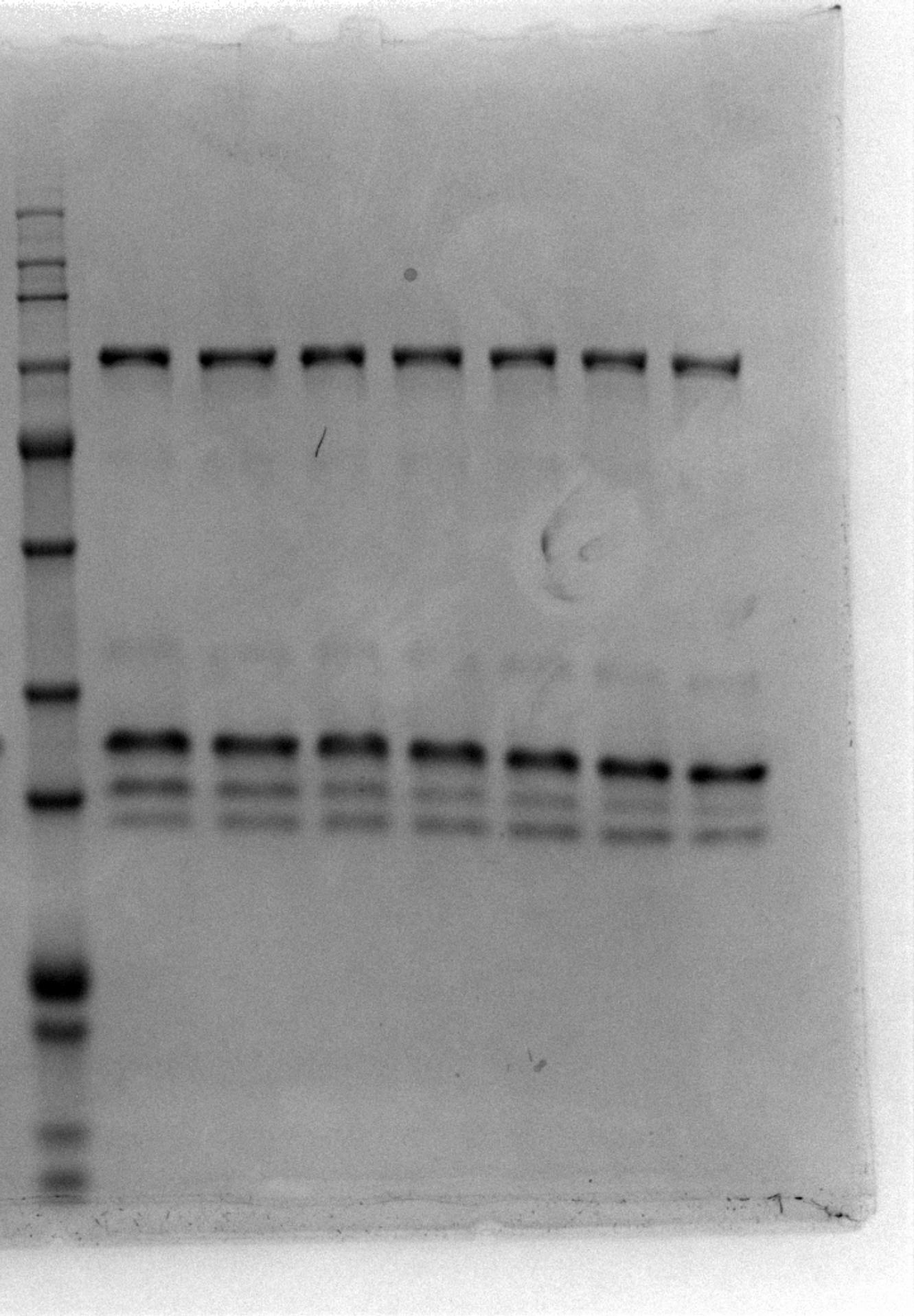

Supplement: Figure 4—source data 1. [file elife-73875-fig4-data1.zip › Figure 4-source data 1 /Figure 4 panel F/iv deg FliK-C +trunc/FliK-C wt.tif]

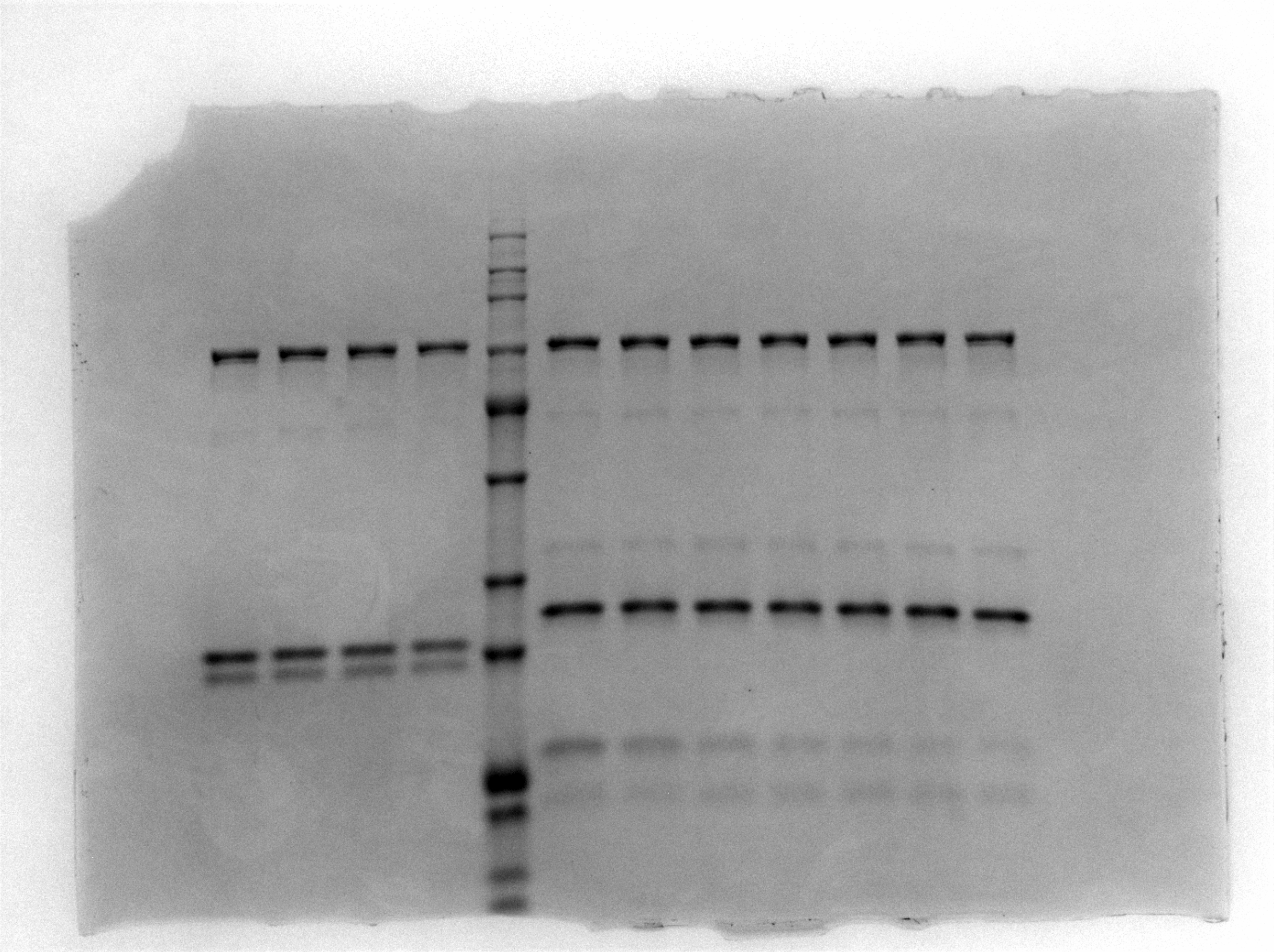

Supplement: Figure 4—source data 1. [file elife-73875-fig4-data1.zip › Figure 4-source data 1 /Figure 4 panel F/iv deg FliK-C +trunc/FliK-C dN44.tif]

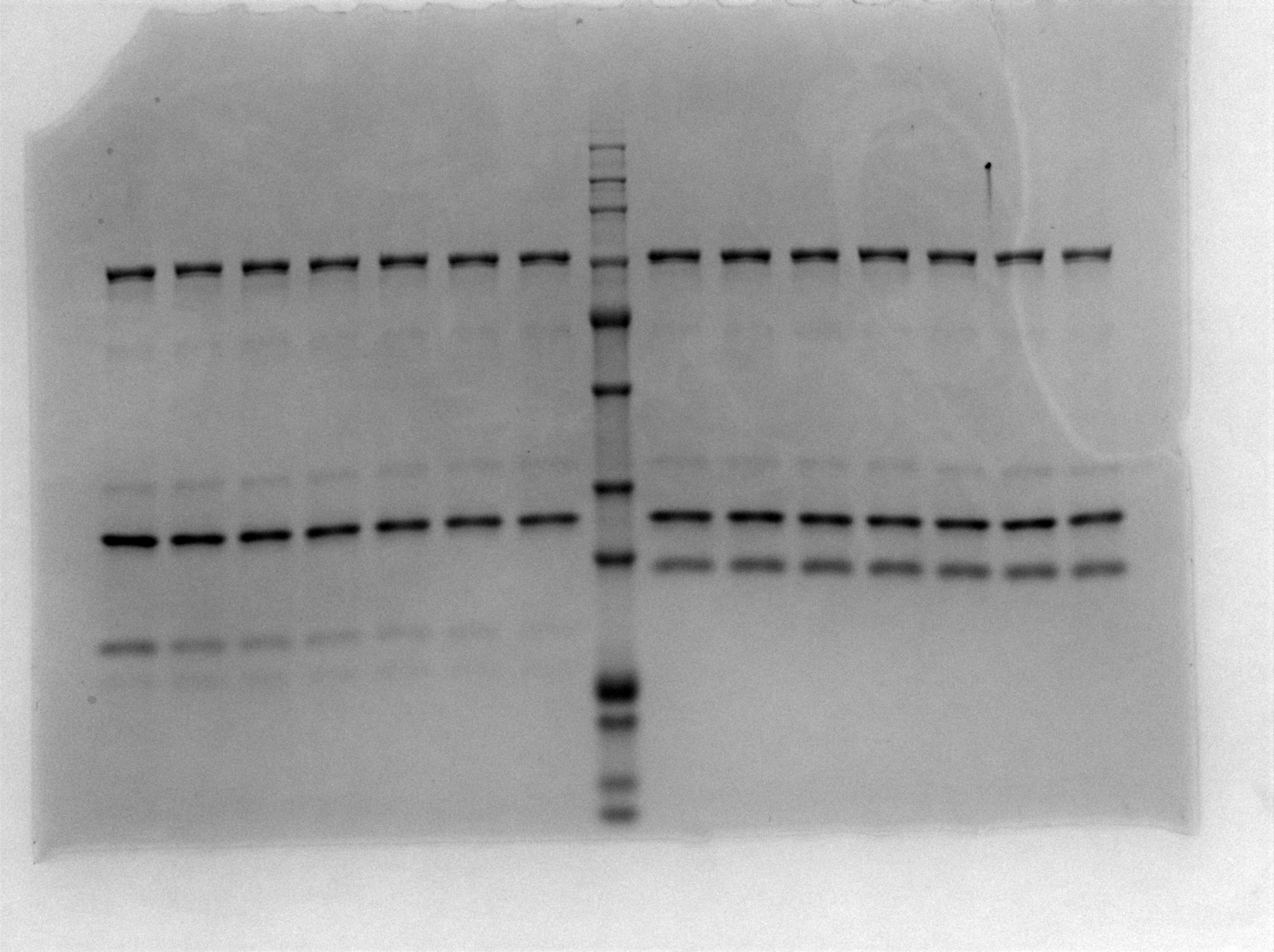

Supplement: Figure 4—source data 1. [file elife-73875-fig4-data1.zip › Figure 4-source data 1 /Figure 4 panel F/iv deg FliK-C +trunc/FliK-C d59-91 + dC44.tif]

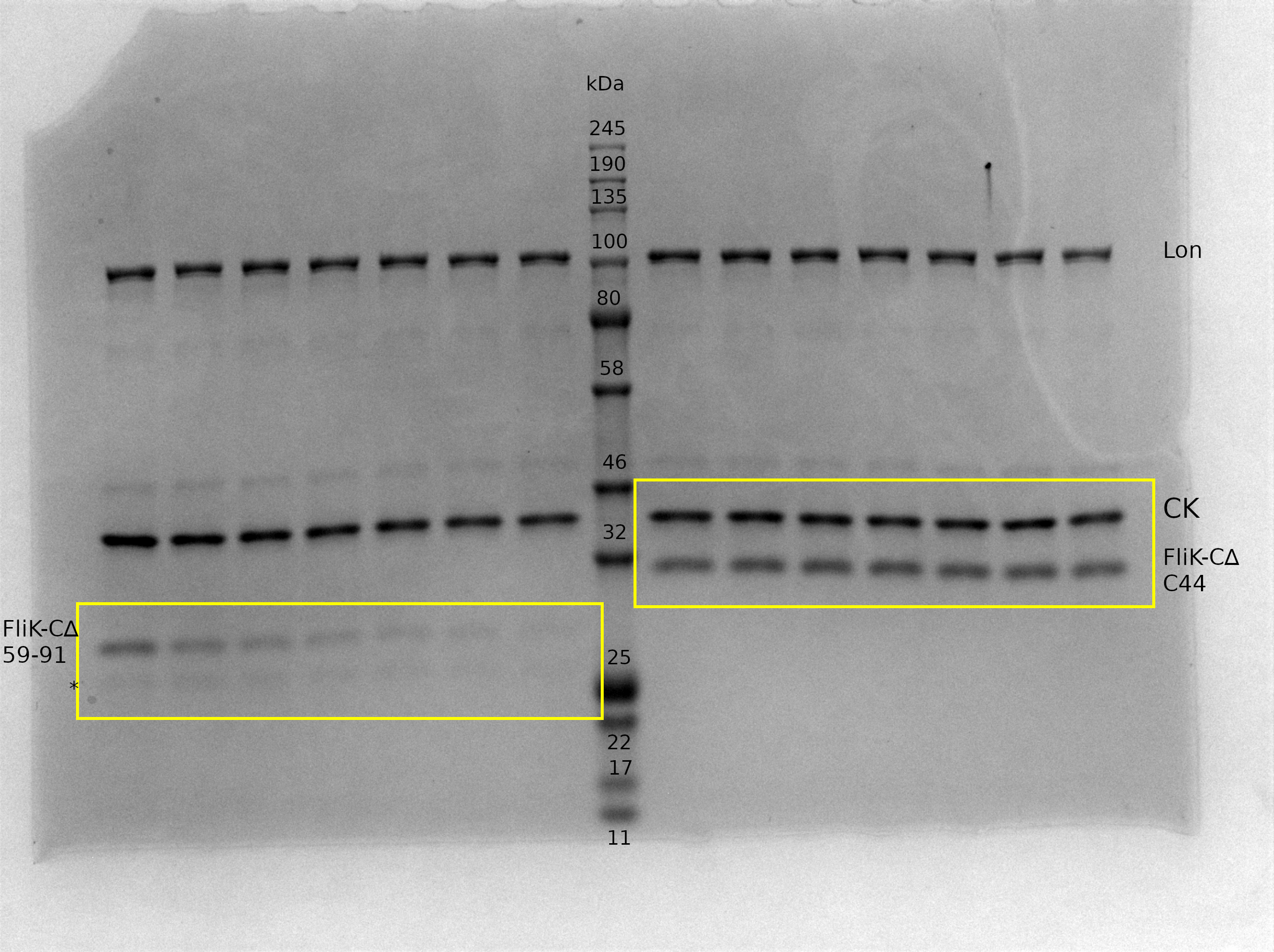

Supplement: Figure 4—source data 1. [file elife-73875-fig4-data1.zip › Figure 4-source data 1 /Figure 4 panel F/iv deg FliK-C +trunc/FliK-C d59-91 + dC44 - Crop_labelled.tif]

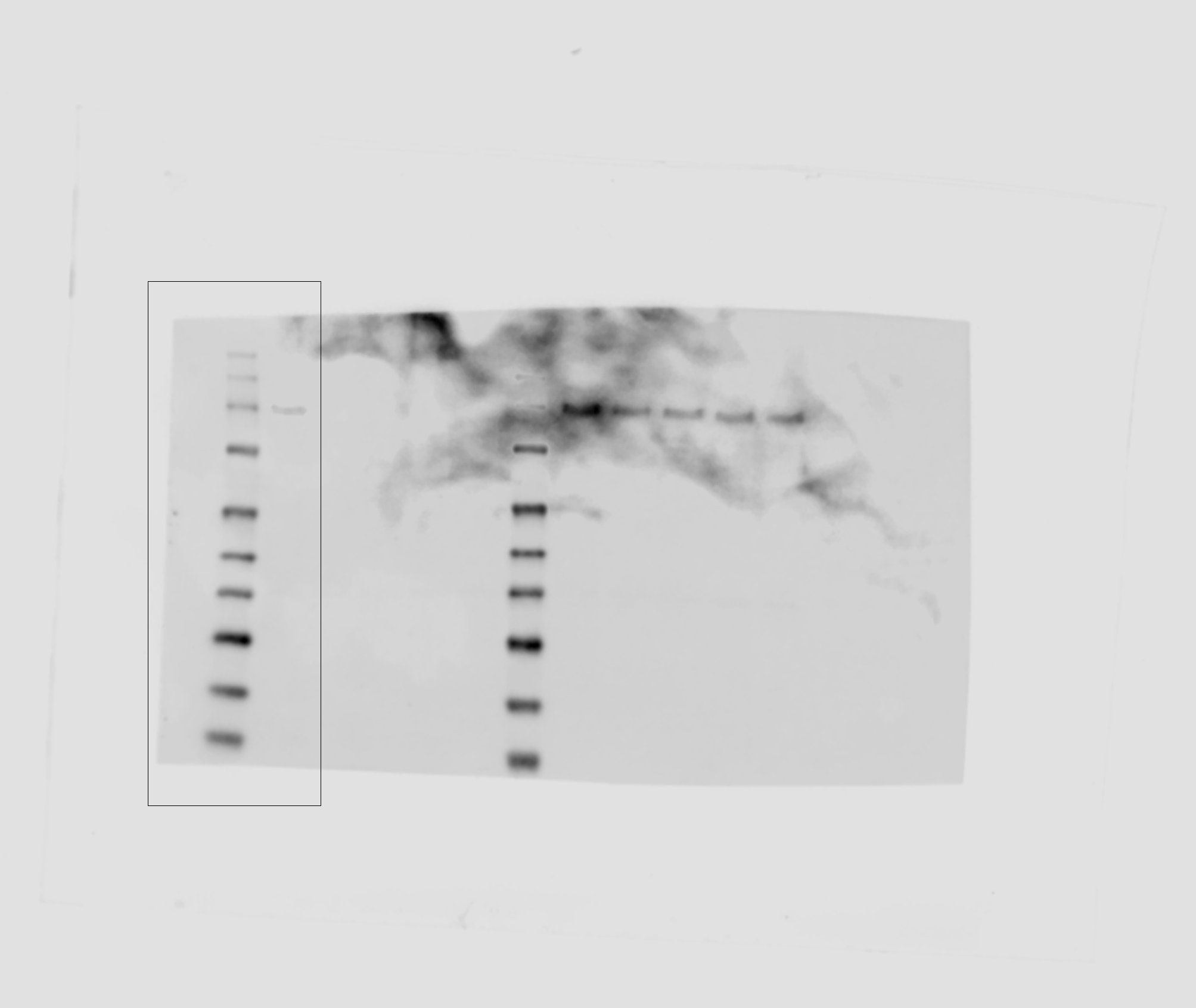

Supplement: Figure 4—figure supplement 1—source data 1. [file elife-73875-fig4-figsupp1-data1.zip › Figure 4-figure supplement 1-source data 1/left panel/anti-FliK-C overlay with protein standard - labelled.tif]

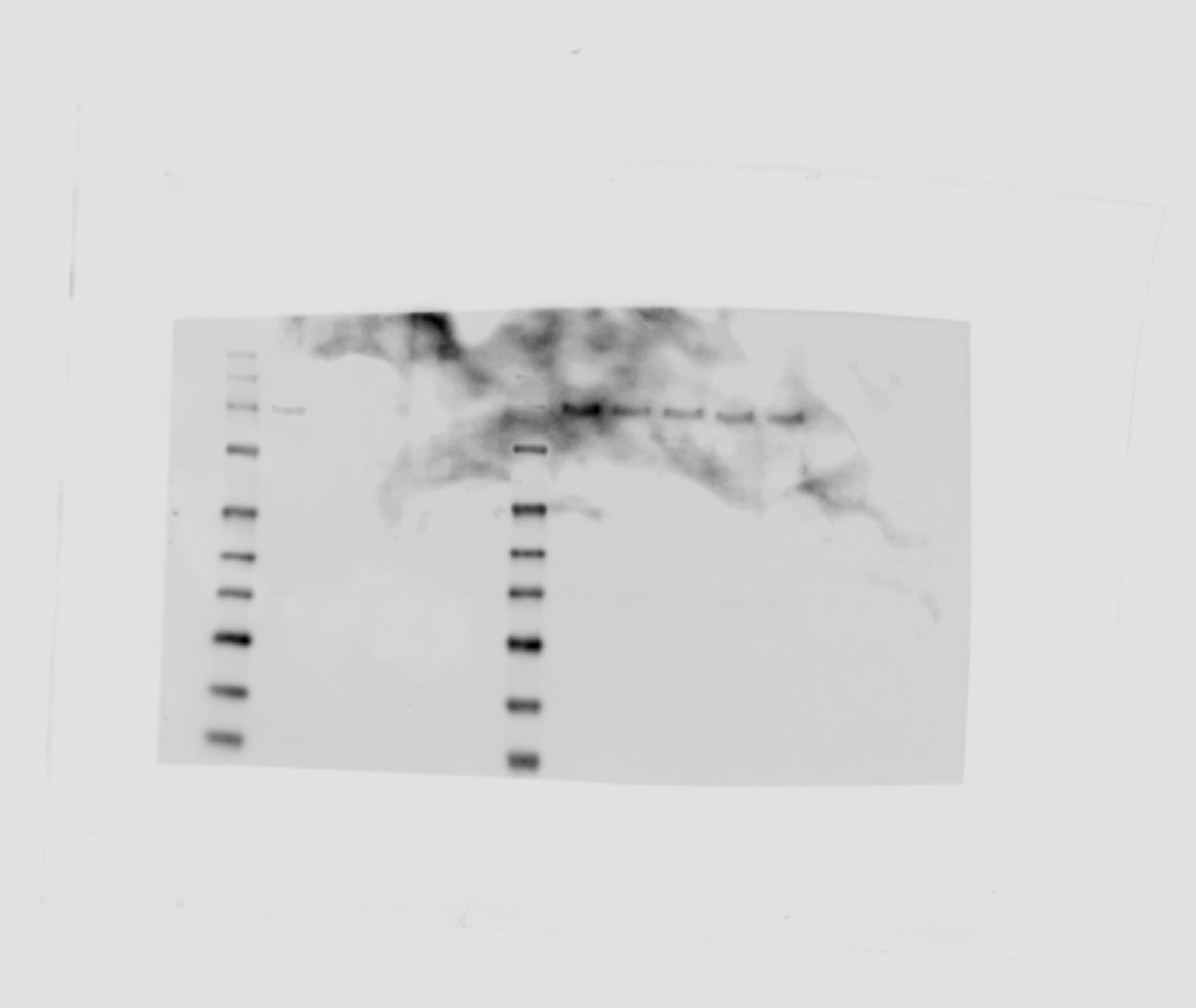

Supplement: Figure 4—figure supplement 1—source data 1. [file elife-73875-fig4-figsupp1-data1.zip › Figure 4-figure supplement 1-source data 1/left panel/anti-FliK-C overlay with protein standard.tif]

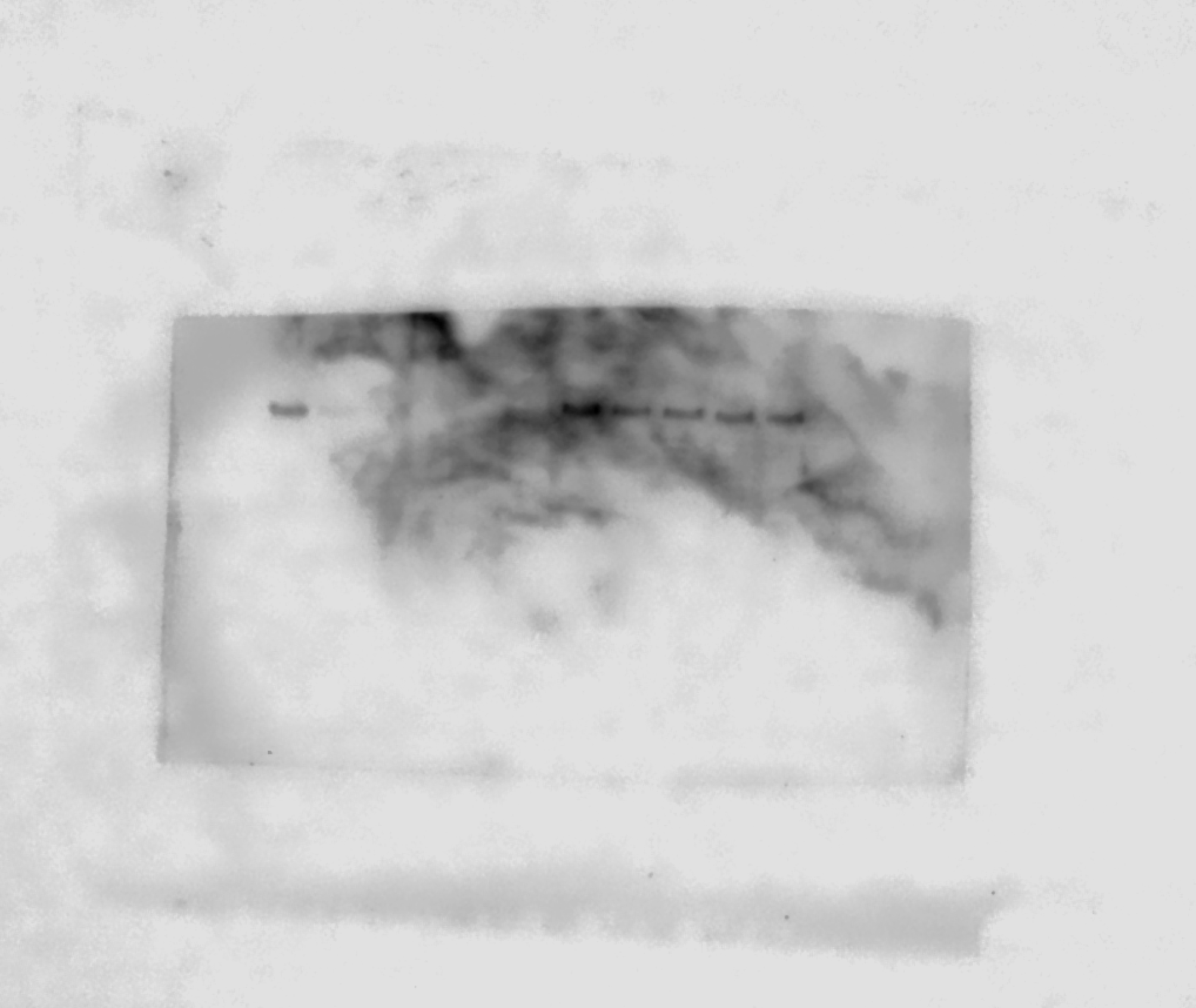

Supplement: Figure 4—figure supplement 1—source data 1. [file elife-73875-fig4-figsupp1-data1.zip › Figure 4-figure supplement 1-source data 1/left panel/anti-FliK-C.tif]

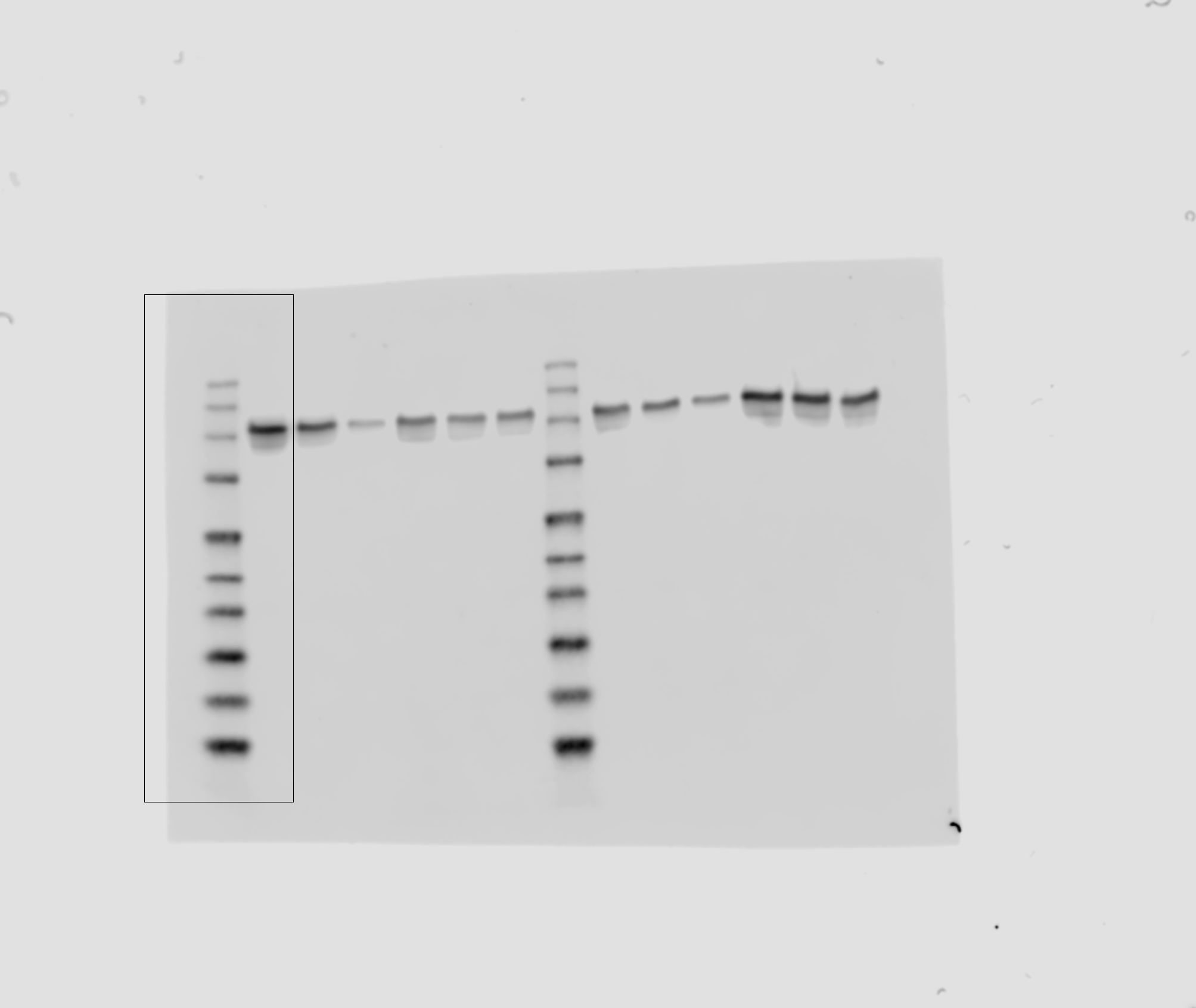

Supplement: Figure 4—figure supplement 1—source data 1. [file elife-73875-fig4-figsupp1-data1.zip › Figure 4-figure supplement 1-source data 1/right panel/anti-FLAG overly with protein standard - labelled.tif]

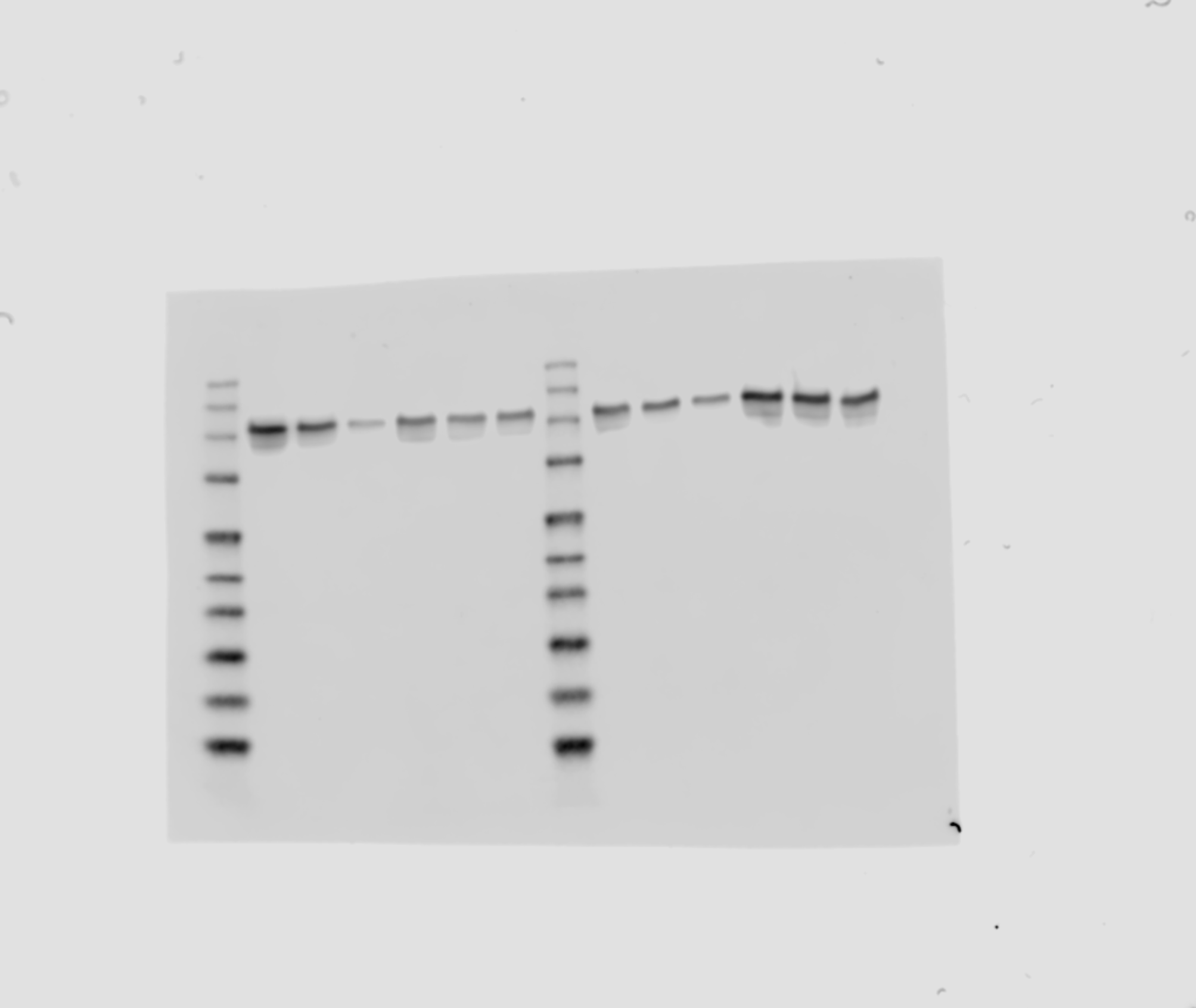

Supplement: Figure 4—figure supplement 1—source data 1. [file elife-73875-fig4-figsupp1-data1.zip › Figure 4-figure supplement 1-source data 1/right panel/anti-FLAG overlay with protein standard.tif]

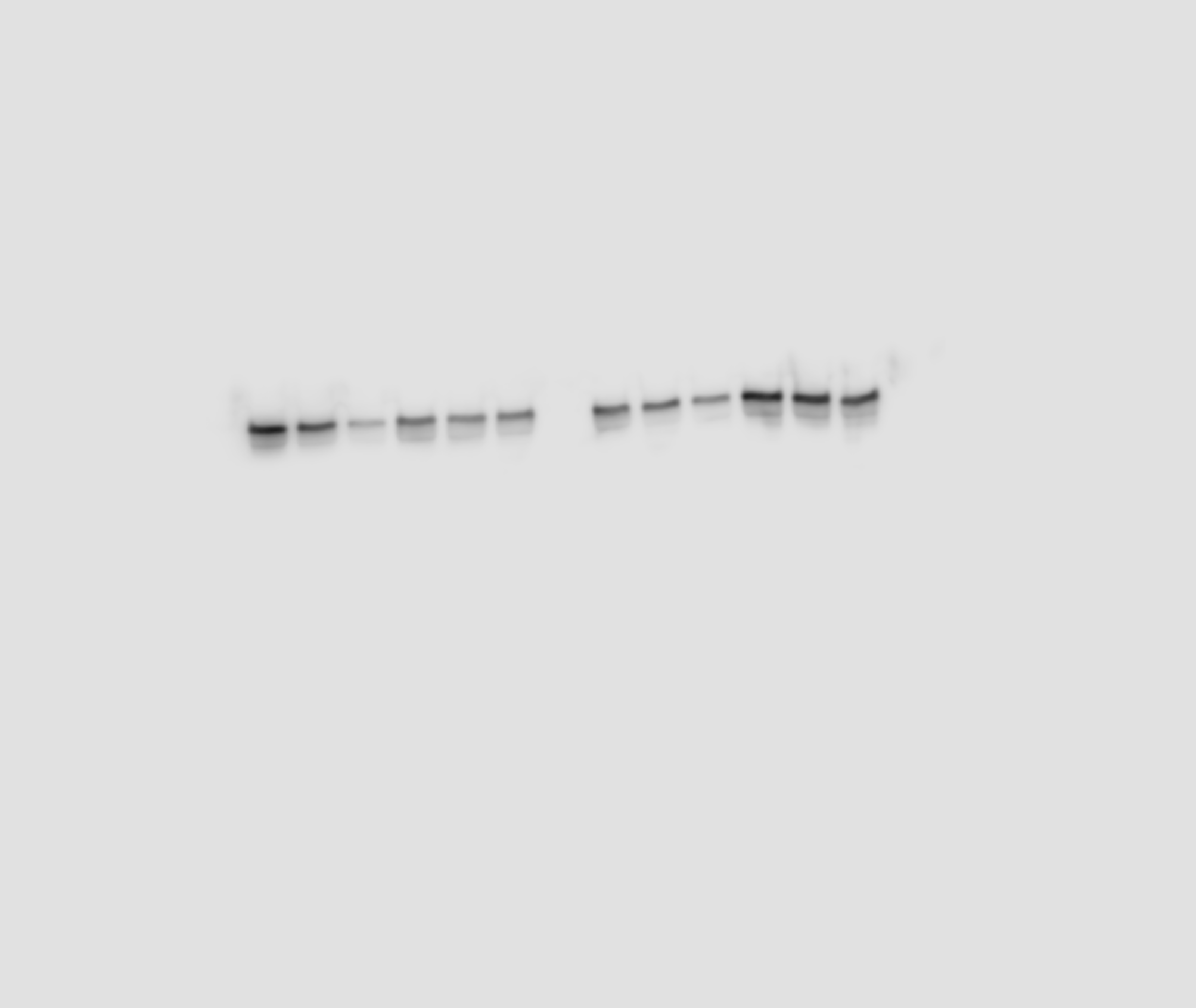

Supplement: Figure 4—figure supplement 1—source data 1. [file elife-73875-fig4-figsupp1-data1.zip › Figure 4-figure supplement 1-source data 1/right panel/anti-FLAG.tif]

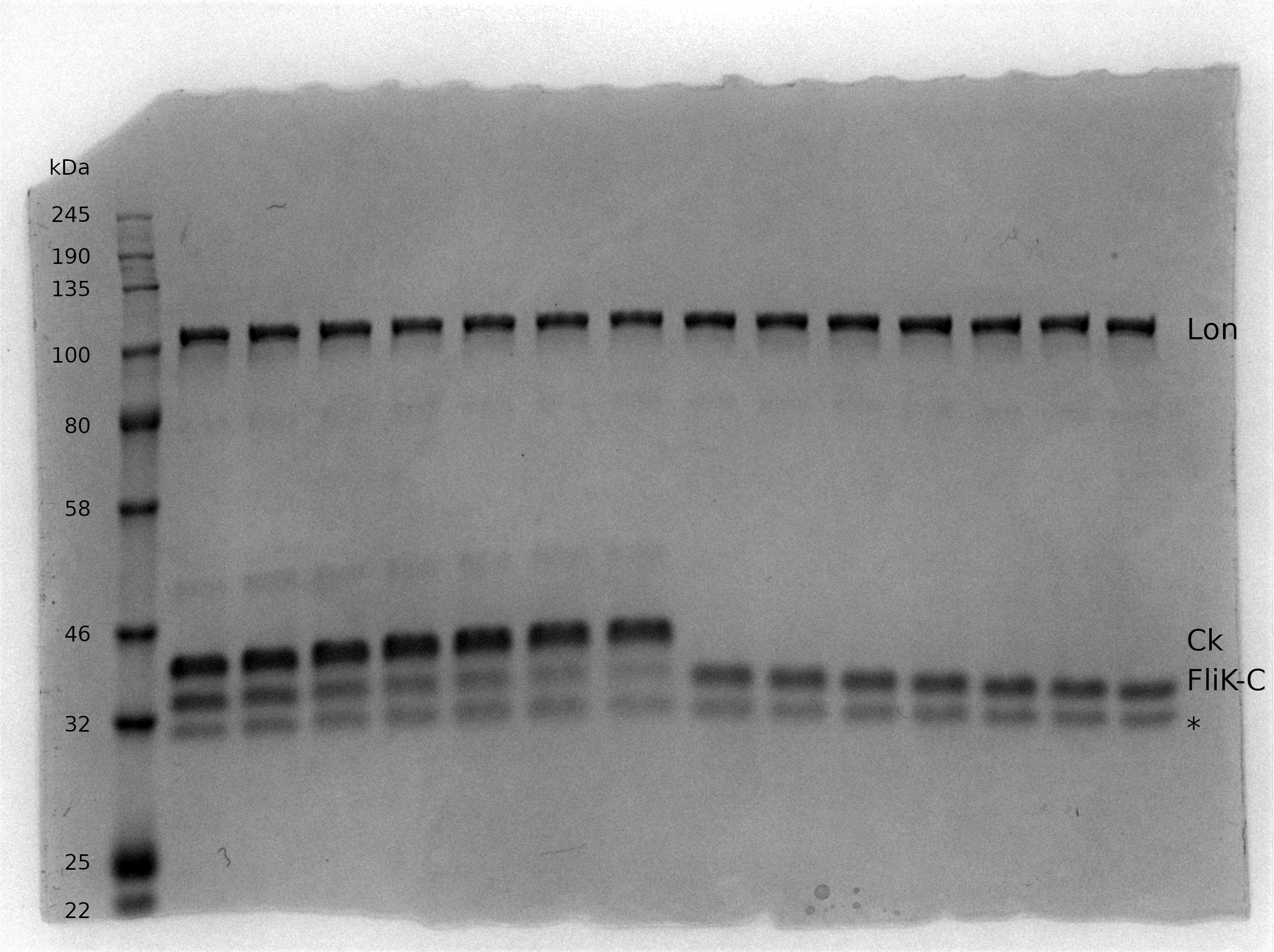

Supplement: Figure 4—figure supplement 2—source data 1. [file elife-73875-fig4-figsupp2-data1.zip › Figure 4-figure supplement 2-source data 1/iv deg FliK-C/Lon+FliK-C+-ATP - Crop_labelled.tif]

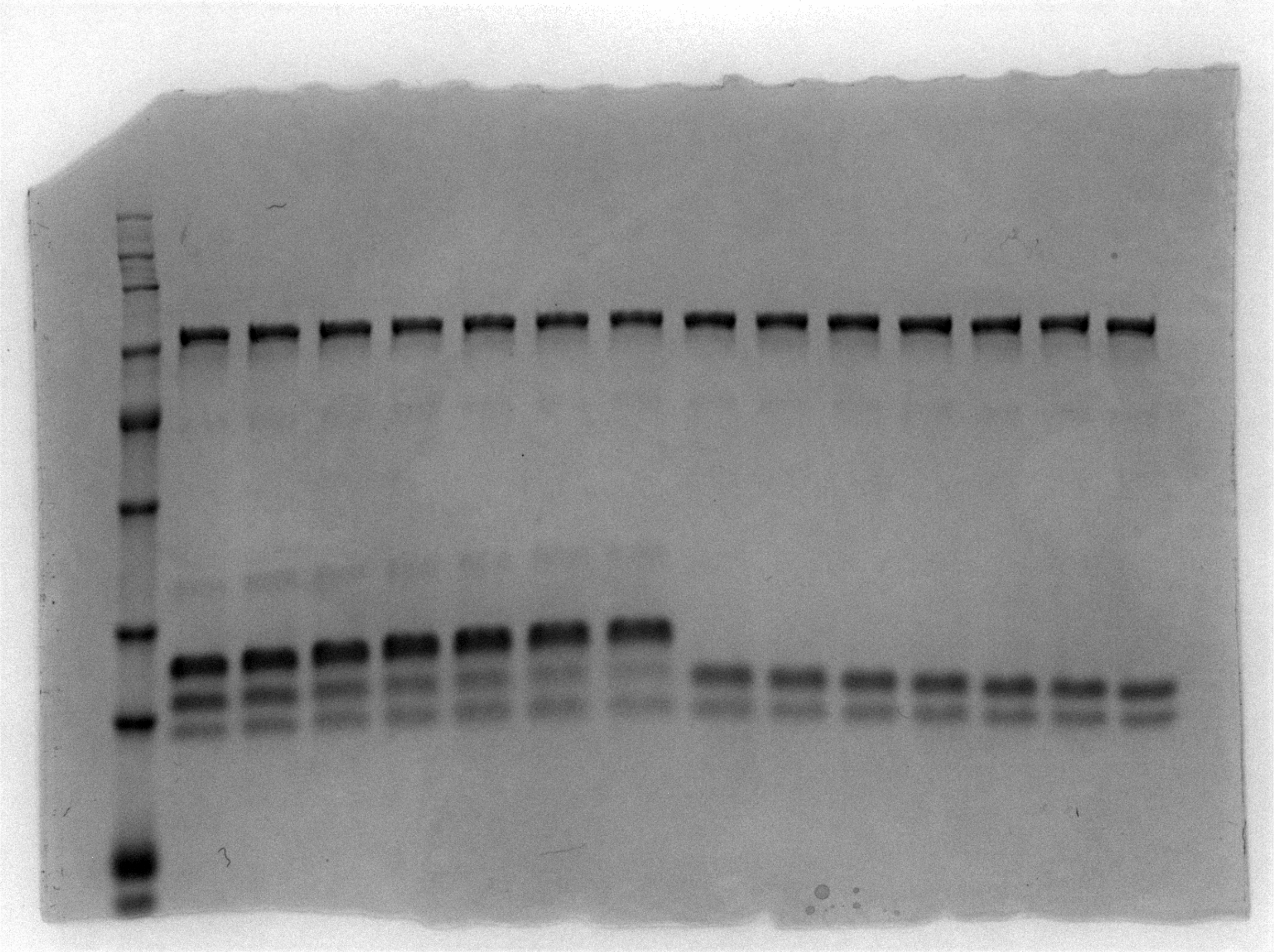

Supplement: Figure 4—figure supplement 2—source data 1. [file elife-73875-fig4-figsupp2-data1.zip › Figure 4-figure supplement 2-source data 1/iv deg FliK-C/Lon+FliK-C+-ATP original.tif]

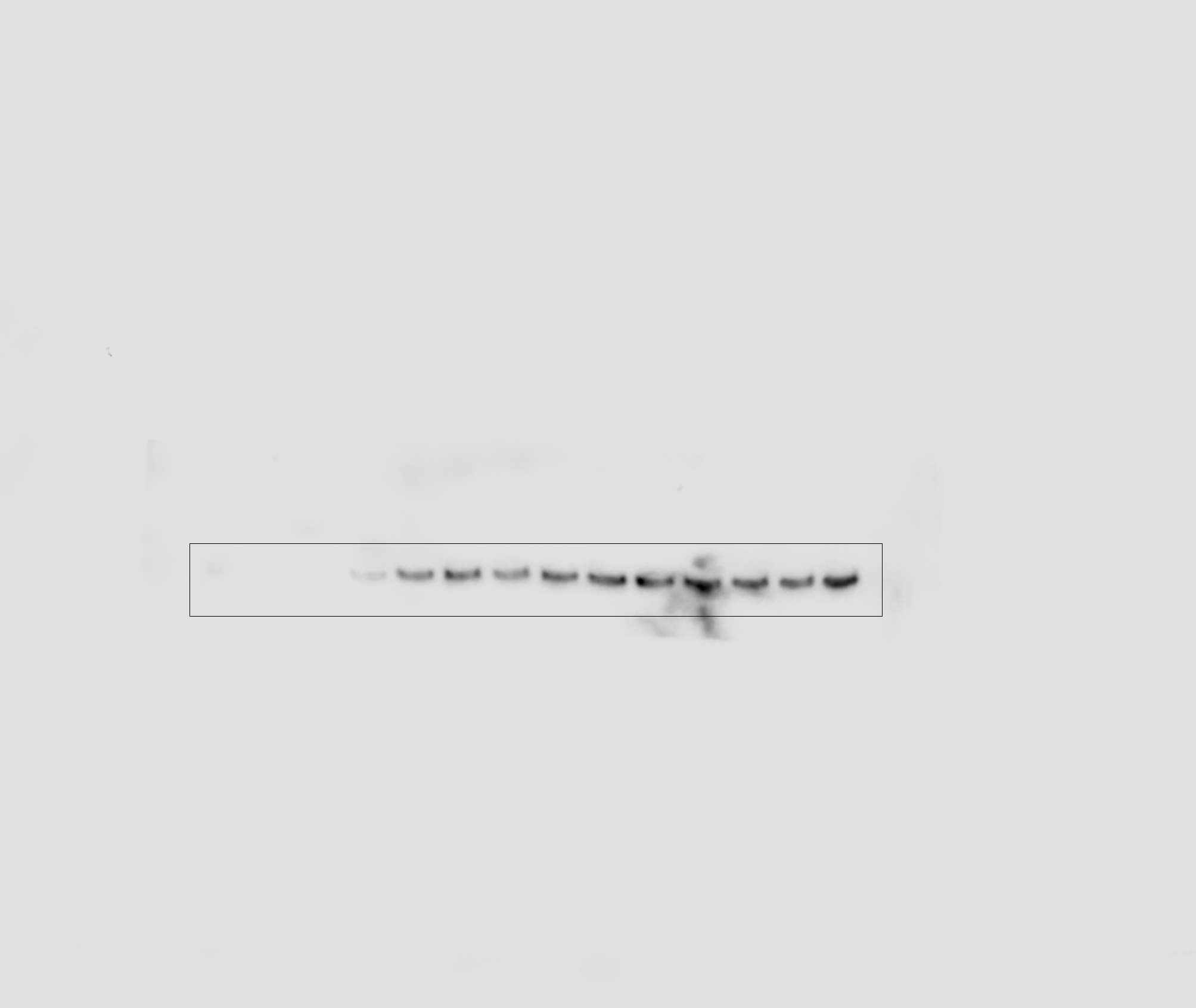

Supplement: Figure 5—source data 1. [file elife-73875-fig5-data1.zip › Figure 5-source data 1/Figure 5 panel B/anti-FliK-C/anti-FliK-C - labelled.tif]

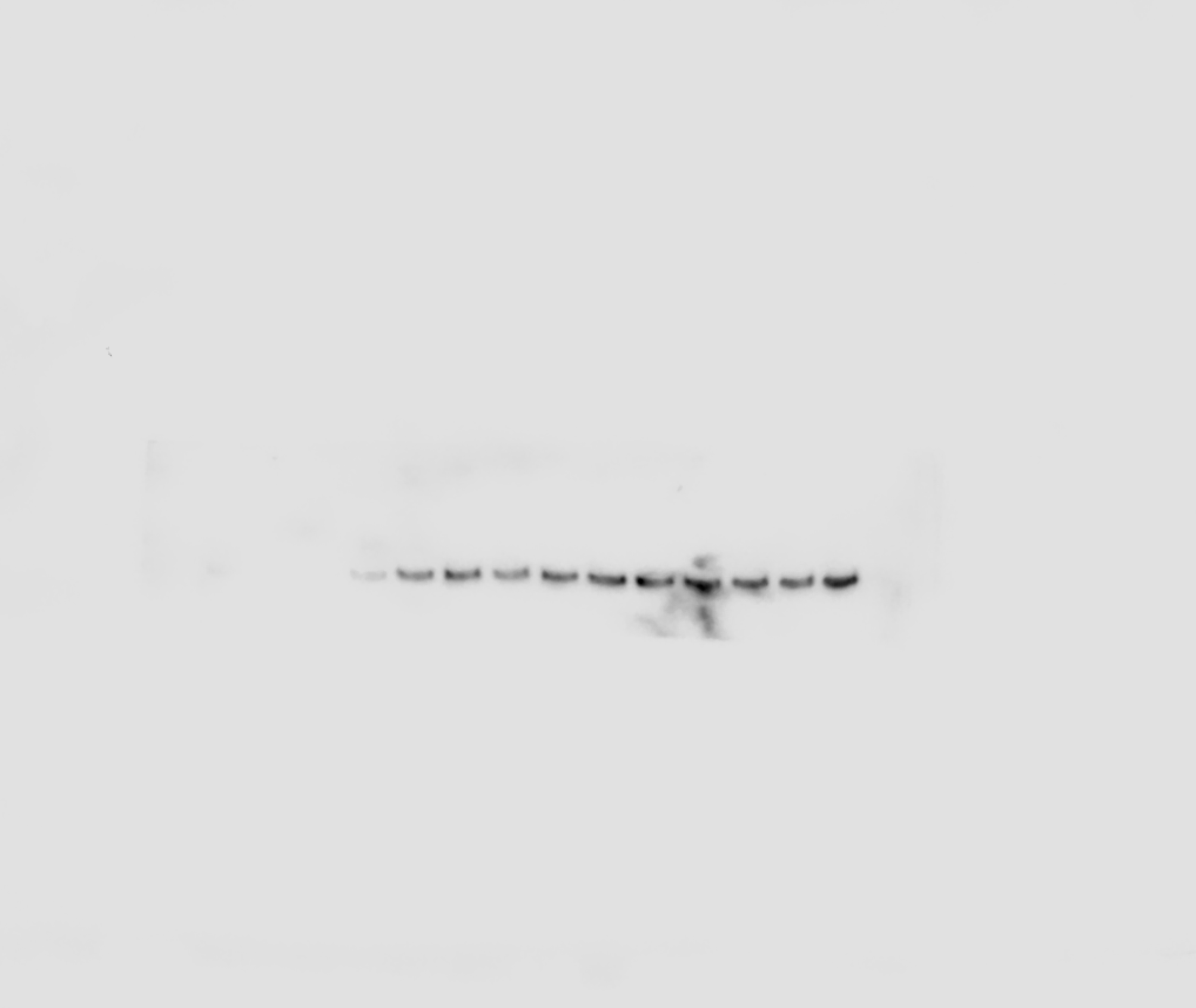

Supplement: Figure 5—source data 1. [file elife-73875-fig5-data1.zip › Figure 5-source data 1/Figure 5 panel B/anti-FliK-C/anti-FliK-C.tif]

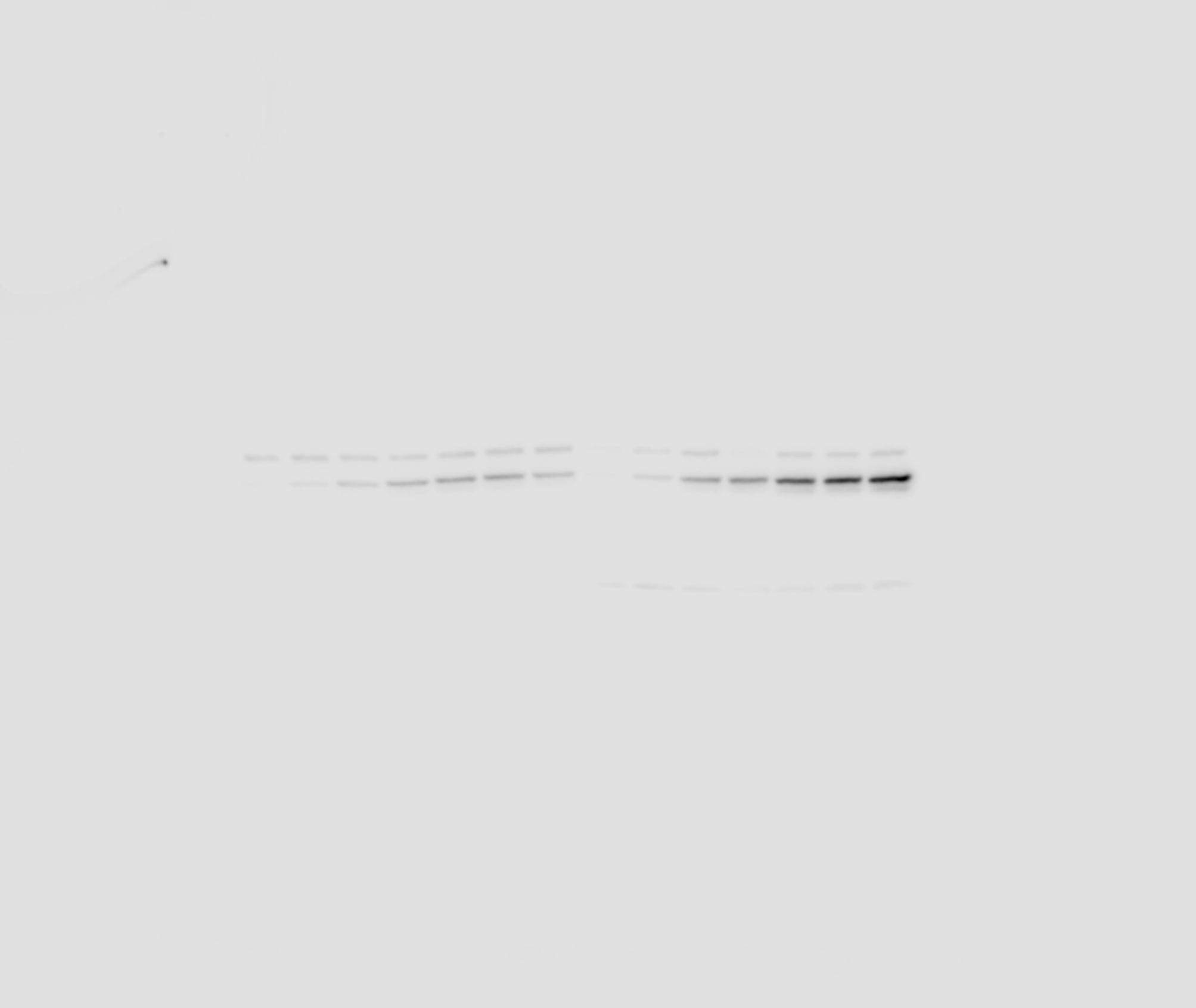

Supplement: Figure 5—source data 1. [file elife-73875-fig5-data1.zip › Figure 5-source data 1/Figure 5 panel B/anti-TipF/anti-TipF.tif]

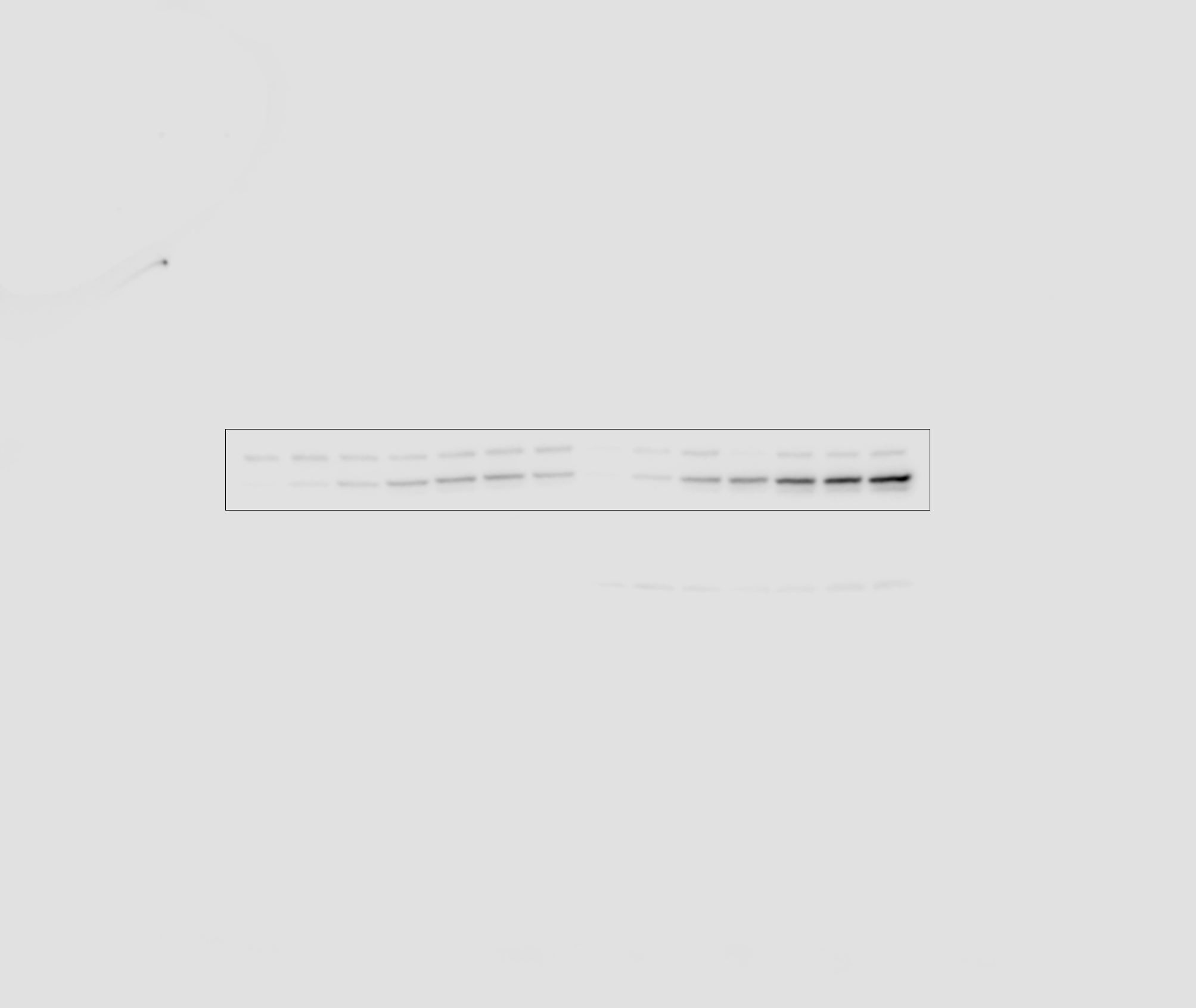

Supplement: Figure 5—source data 1. [file elife-73875-fig5-data1.zip › Figure 5-source data 1/Figure 5 panel B/anti-TipF/anti-TipF - labelled.tif]

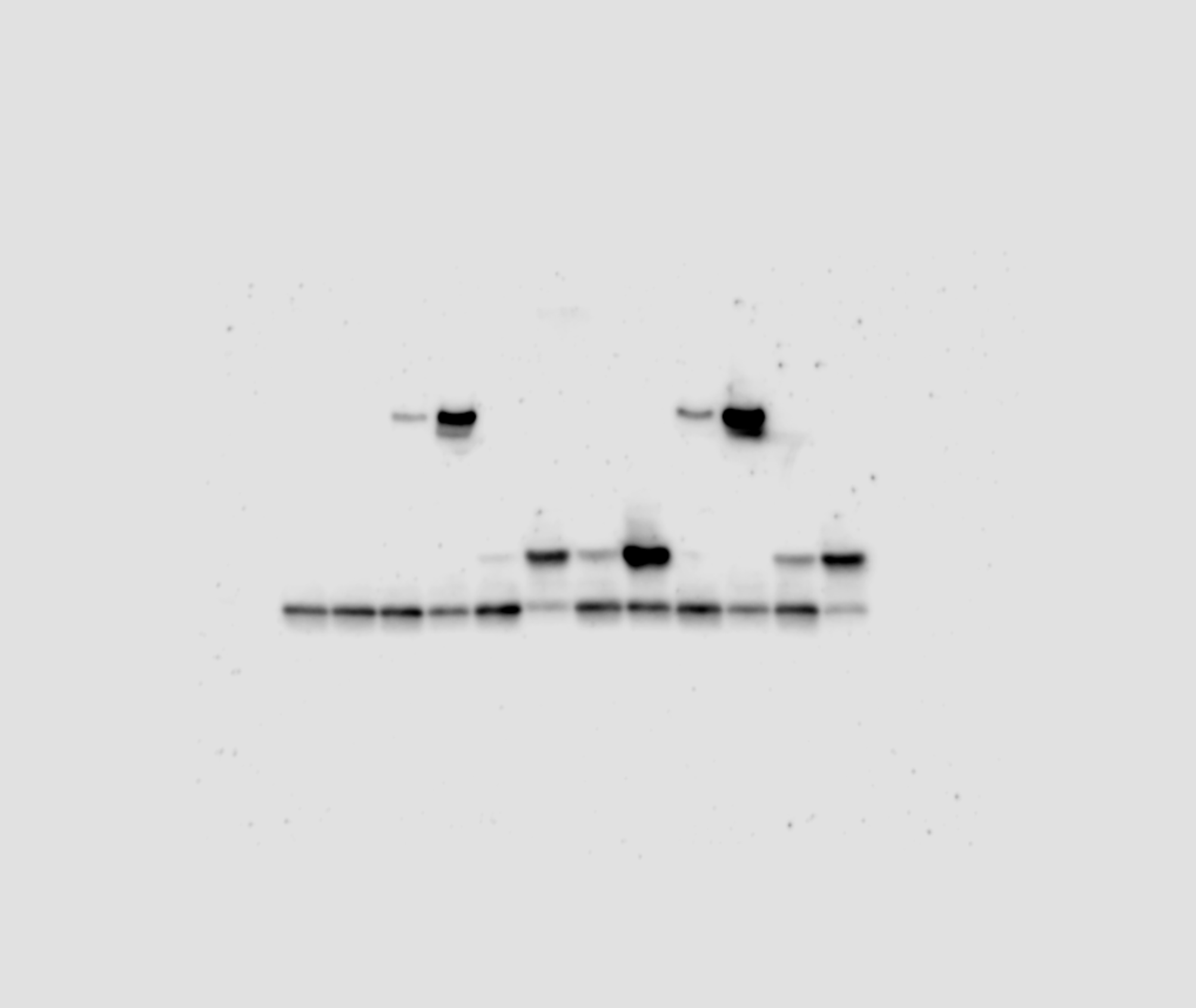

Supplement: Figure 5—source data 1. [file elife-73875-fig5-data1.zip › Figure 5-source data 1/Figure 5 panel G/anti-FLAG/anti-FLAG.tif]

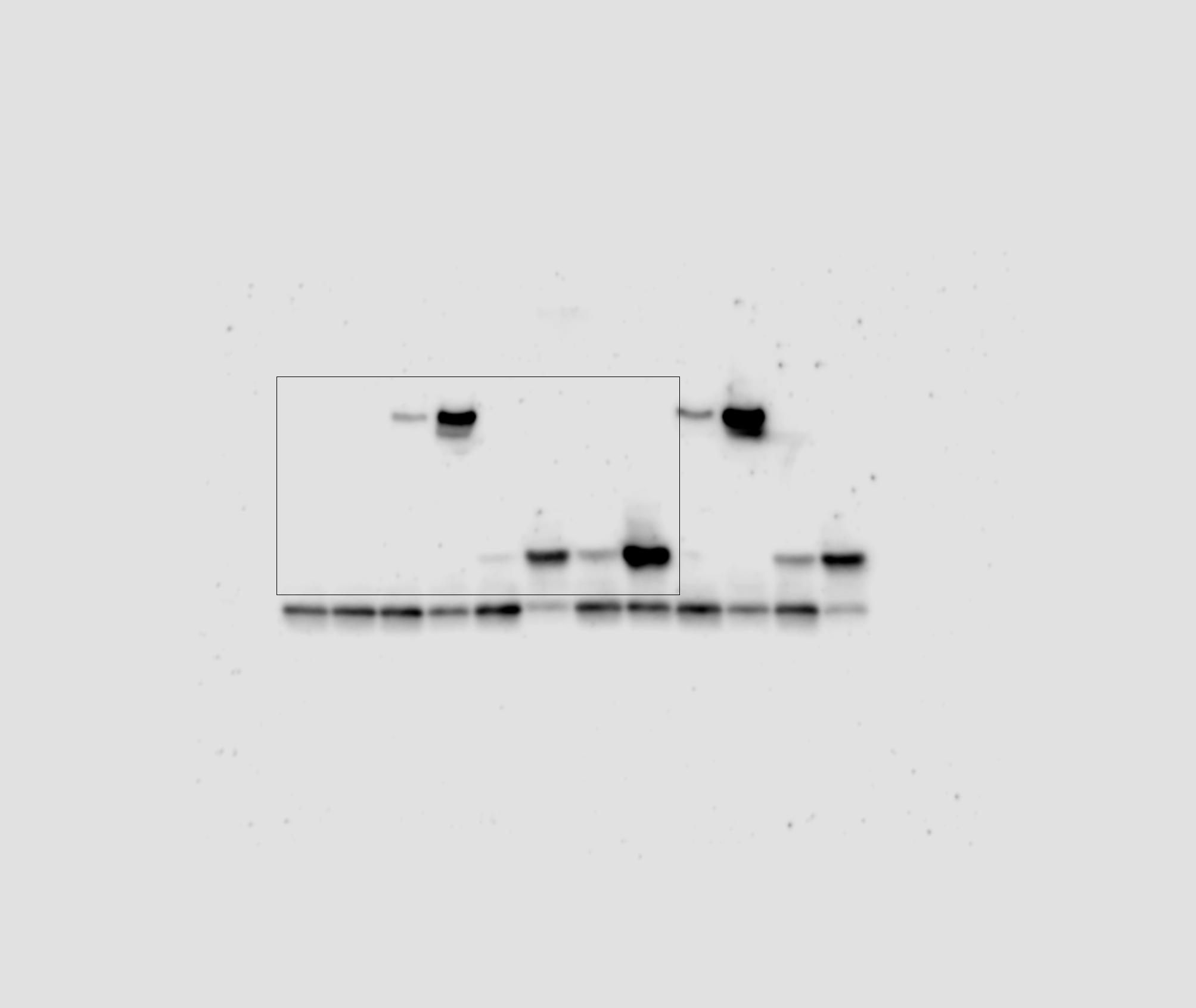

Supplement: Figure 5—source data 1. [file elife-73875-fig5-data1.zip › Figure 5-source data 1/Figure 5 panel G/anti-FLAG/anti-FLAG - labelled.tif]

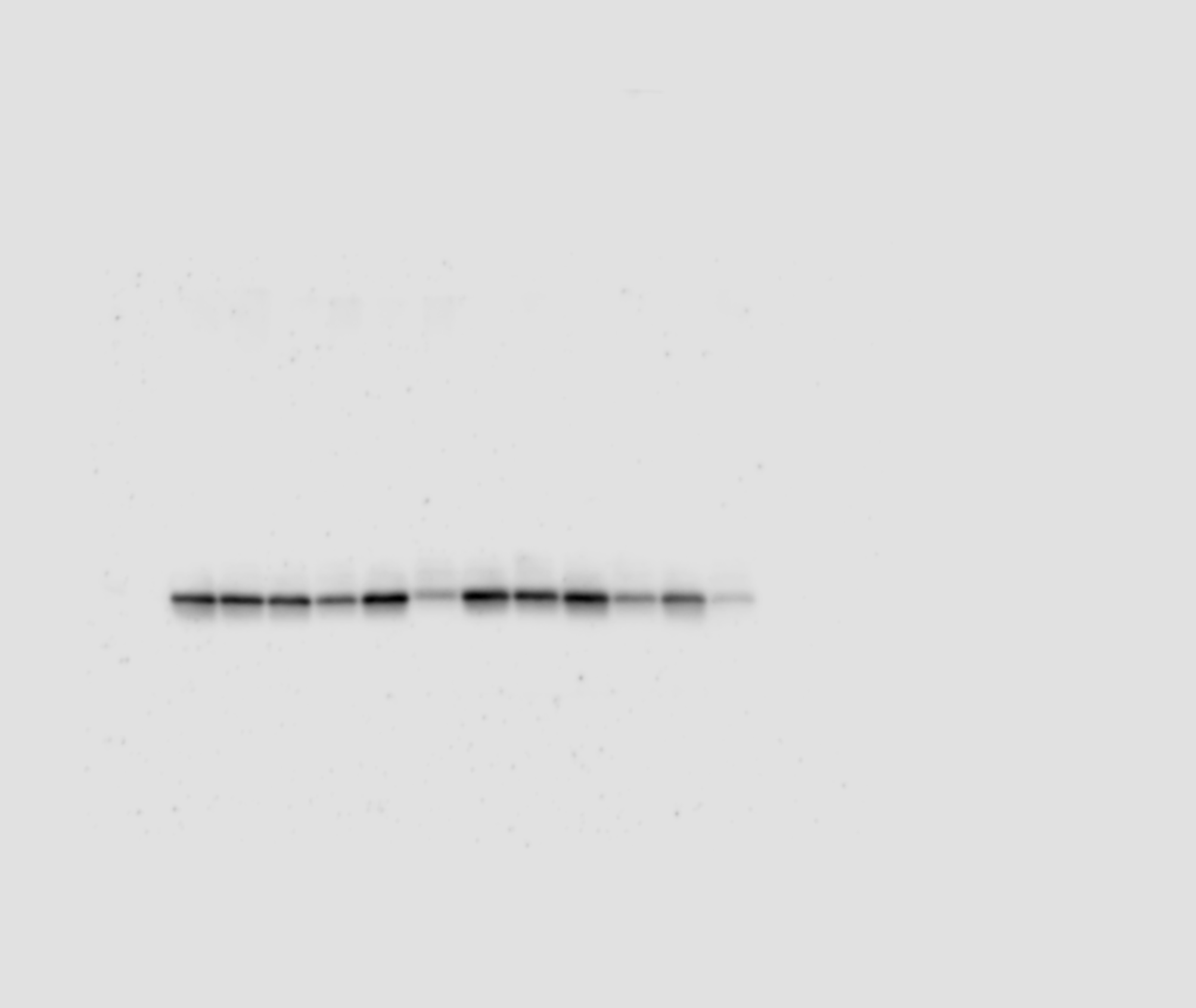

Supplement: Figure 5—source data 1. [file elife-73875-fig5-data1.zip › Figure 5-source data 1/Figure 5 panel G/anti-Flagellins/anti-Flagellins.tif]

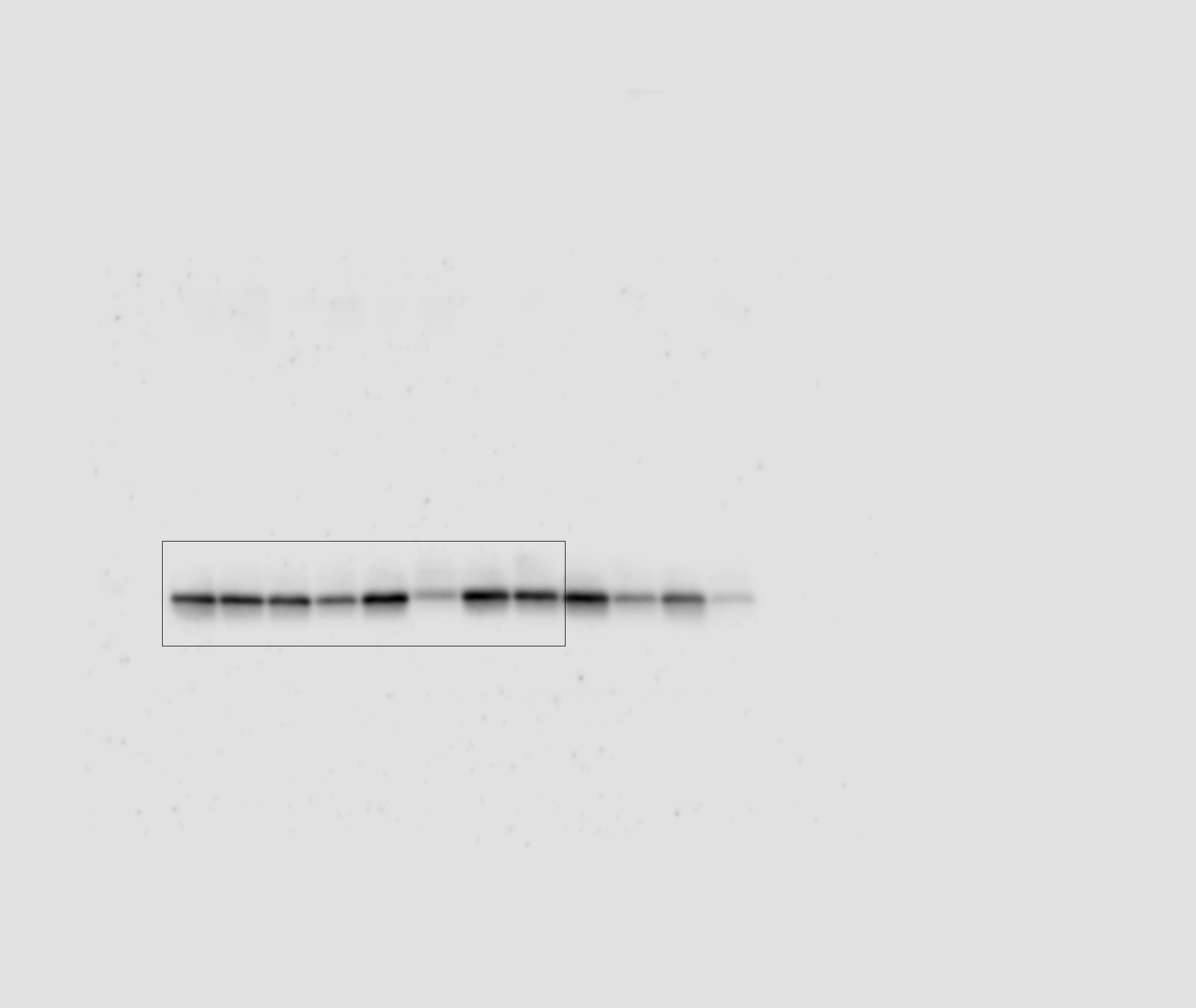

Supplement: Figure 5—source data 1. [file elife-73875-fig5-data1.zip › Figure 5-source data 1/Figure 5 panel G/anti-Flagellins/anti-Flagellins- labelled.tif]

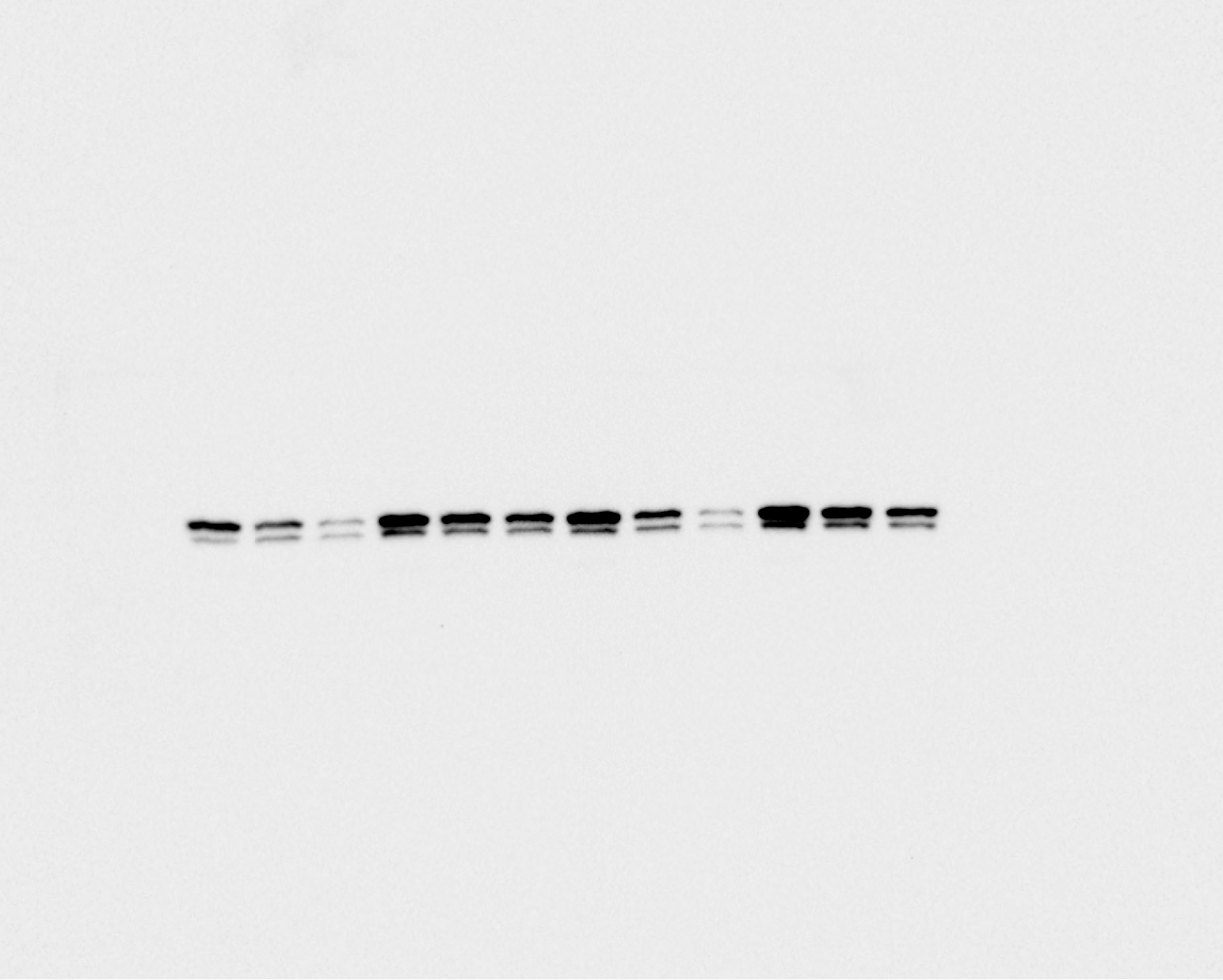

Supplement: Figure 6—figure supplement 1—source data 1. [file elife-73875-fig6-figsupp1-data1.zip › Figure 6-figure supplement 1-source data 1/anti-FLAG/anti-FLAG.tif]

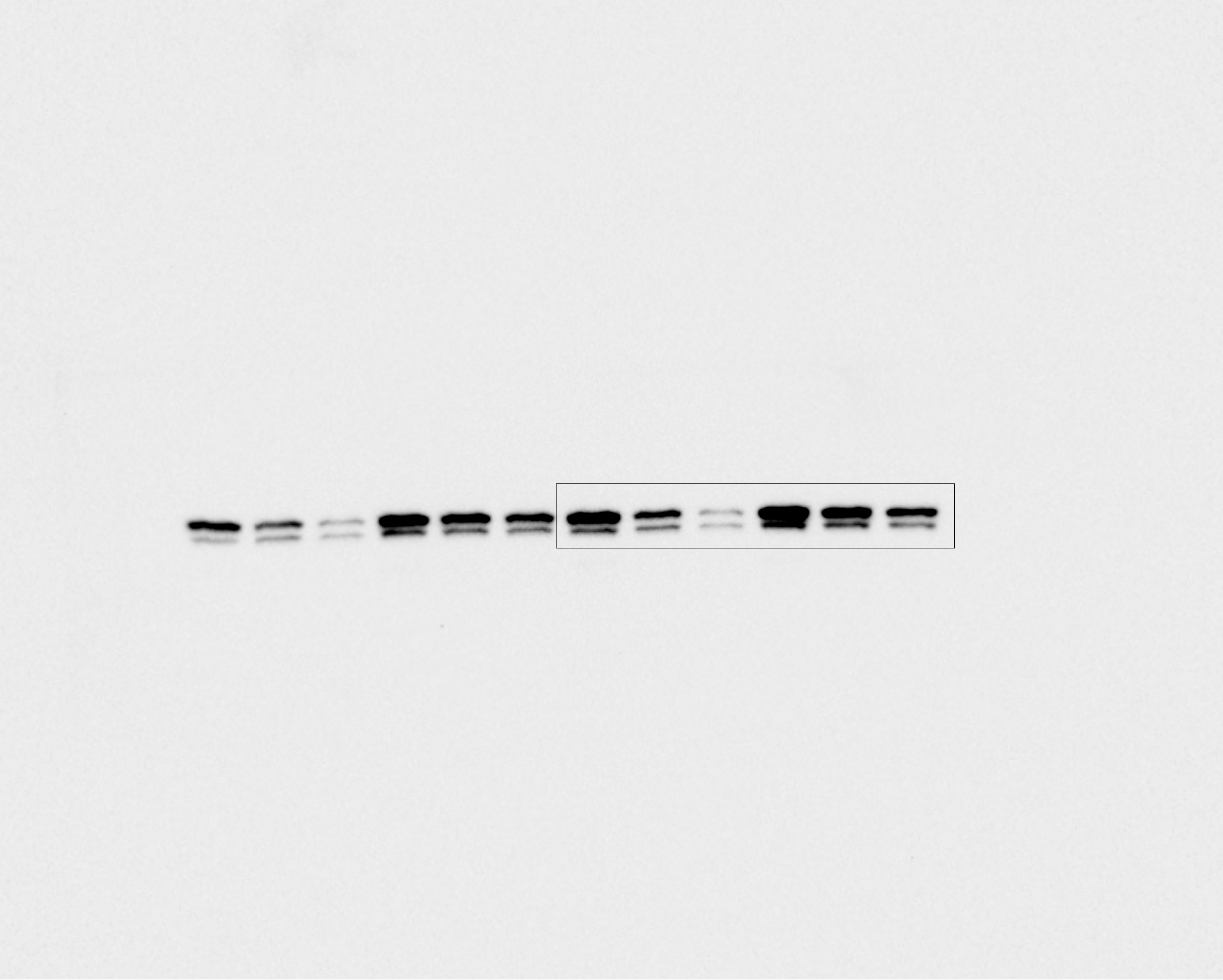

Supplement: Figure 6—figure supplement 1—source data 1. [file elife-73875-fig6-figsupp1-data1.zip › Figure 6-figure supplement 1-source data 1/anti-FLAG/anti-FLAG - labelled.tif]
